# Buried in water, burdened by nature—Resilience carried the Iron Age people through Fimbulvinter

**DOI:** 10.1371/journal.pone.0231787

**Published:** 2020-04-21

**Authors:** Markku Oinonen, Teija Alenius, Laura Arppe, Hervé Bocherens, Heli Etu-Sihvola, Samuli Helama, Heli Huhtamaa, Maria Lahtinen, Kristiina Mannermaa, Päivi Onkamo, Jukka Palo, Antti Sajantila, Kati Salo, Tarja Sundell, Santeri Vanhanen, Anna Wessman

**Affiliations:** 1 Finnish Museum of Natural History, University of Helsinki, Helsinki, Finland; 2 Department of Cultures, Archaeology, University of Helsinki, Helsinki, Finland; 3 Eberhard-Karls Universität Tübingen, Tübingen, Germany; 4 Senckenberg Centre for Human Evolution and Palaeoenvironment (SHEP) at University of Tübingen, Tübingen, Germany; 5 Natural Resources Institute Finland, Rovaniemi, Finland; 6 Heidelberg Centre for the Environment, University of Heidelberg, Heidelberg, Germany; 7 Finnish Food Authority, Helsinki, Finland; 8 Organism and Evolutionary Biology Research Programme, University of Helsinki, Helsinki, Finland; 9 Physiology and Genetics, University of Turku, Turku, Finland; 10 Department of Forensic Medicine, University of Helsinki, Helsinki, Finland; 11 Forensic Genetics Unit, Finnish Institute for Health and Welfare, Helsinki, Finland; 12 Institute of Biotechnology, University of Helsinki, Helsinki, Finland; 13 Statens Historiska Museer, Lund, Sweden; University at Buffalo - The State University of New York, UNITED STATES

## Abstract

Levänluhta is a unique archaeological site with the remains of nearly a hundred Iron Age individuals found from a water burial in Ostrobothnia, Finland. The strongest climatic downturn of the Common Era, resembling the great Fimbulvinter in Norse mythology, hit these people during the 6^th^ century AD. This study establishes chronological, dietary, and livelihood synthesis on this population based on stable carbon and nitrogen isotopic and radiocarbon analyses on human remains, supported by multidisciplinary evidence. Extraordinarily broad stable isotopic distribution is observed, indicating three subgroups with distinct dietary habits spanning four centuries. This emphasizes the versatile livelihoods practiced at this boundary of marine, freshwater, and terrestrial ecosystems. While the impact of the prolonged cold darkness of the 6^th^ century was devastating for European communities relying on cultivation, the broad range of livelihoods provided resilience for the Levänluhta people to overcome the abrupt climatic decline.

## Introduction

Mediterranean historical sources identify a mystery cloud obstructing the Sun at AD 536/537[1]. The year without the Sun is observed in tree rings as a negative growth anomaly throughout the Northern Hemisphere (NH)[2]. An even larger tree-growth decline is observed during the AD 540s, and discussion of the anomaly has evolved from a single mystery cloud to a decade-scale climatic catastrophe as a result of multiple volcanic eruptions of AD 536–550 [3,4]. The anomalous years probably triggered a longer climatic disturbance lasting until AD 570[5], or even beyond, known as the “Late Antique Little Ice Age” (henceforth LALIA)[6]. This cold and dark period—further stressed by the breakout of the Justinian Plague in AD 542—coincided with the rise and fall of empires, human migrations, and political upheavals[4,6].

The volcanic winter of the AD 540s, recently linked to the Ilopango eruption in El Salvador[7], featured a drastic reduction of solar irradiance for several years in AD 541–544 (S1 Appendix). This was observed in Northern Finland[8], reducing the temperature and photosynthetic rate, and thus primary production[9]. The severe effects of distant volcanic eruptions on agricultural communities across the NH have been recently demonstrated[10]—crop losses and famines in 17^th^ century AD Finland have been shown to have resulted from tropical volcanic eruptions (S1 Appendix). As the AD 536–550 climatic catastrophe was the most severe in the last 2500 years[3,11], one can hypothesize that its consequences for communities relying on cultivation were even more catastrophic. Indeed, the abandonment of Iron Age settlements in Scandinavia and Estonia after AD 550 may have been a result of the climatic downturn and cultivational challenges[12,13].

**Fig 1:**
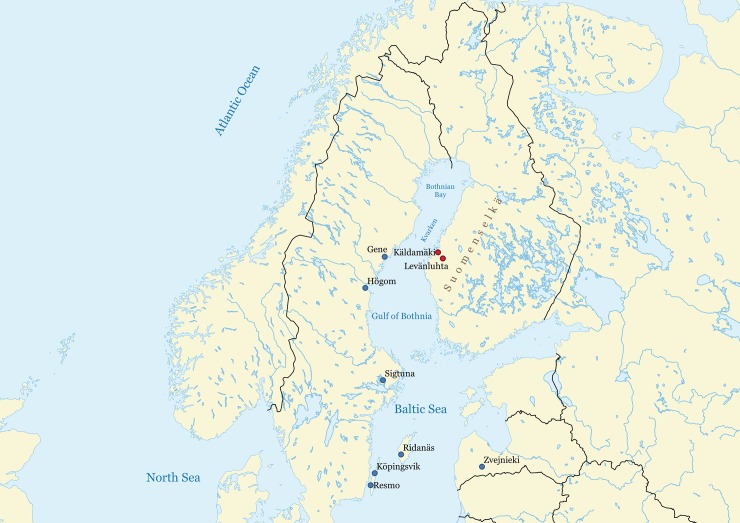
Site locations. Locations of Levänluhta, Käldamäki, and the sites mentioned in the text are shown. Levänluhta, in particular, is located near the Suomenselkä ridgeline, providing access to the vast northern and eastern forests and lake region. The map was created based on Natural Earth data (https://www.naturalearthdata.com/).

This work studies the dietary habits and livelihoods of a population that experienced the AD 536–550 climatic downturn in Southern Ostrobothnia, Finland (Fig 1). Early scholars[14,15] only fragmentarily tell of the people of the North, and these frontiers of ancient civilizations have remained a kind of an unknown otherworld, associated with mystical elements of the seasonal change from long darkness to midnight sun. However, already paralleling the early civilizations of the Mediterranean, the Northerners were involved in cultural connections and trading networks across the Europe[16–18], linking them to the world system. In the Southern Ostrobothnian inland (Fig 1), nearly a hundred Iron Age (ca. AD 300–800) individuals were buried in water in Levänluhta[19–22]. The site, presently consisting of three springs releasing red iron-containing water, has been one of the most intriguing archaeological mysteries within Finland (S2 Appendix). The lives of these people coincided with the greatest global-scale changes of the Common Era: a land uplift of one meter per century (S3 Appendix) and an abrupt period without the Sun. Using dietary stable isotope studies[23–25] combined with chronological analyses[26,27] on human bone collagen samples, it is possible to understand the factors that allowed the Iron Age populations at the northern edge of European civilization to carry themselves through these challenges. A synthesis of this watery burial of worldwide uniqueness is established by tying the existing multidisciplinary knowledge together.

## Materials and methods

### Osteological analyses

The study material consists of left thighbones (femora) of human remains from sites of Levänluhta and Käldamäki in Southern Ostrobothnia, Finland, considered as spring-containing water burials (Fig 1, Fig 1E and 1F in S2 Appendix). All necessary permits were obtained for the described study, which complied with all relevant regulations. Particularly, permission to sample and analyse the bone material was obtained from the Finnish Heritage Agency, Helsinki, Finland, in which the material is stored. Käldamäki is a smaller site, 25 km northwest from Levänluhta, and dating to nearly the same centuries. Left femora were chosen for detailed analyses to maximize unambiguous identifications of individuals whose remains are totally disarticulated. The bones were variably damaged and required thus multifaceted osteological analyses. Bones were identified as left femora by skeletal element and size. Subadult age estimations were based on the size of the bones and the epiphyseal closure[28–31]. Adult age estimations on femora were attempted based on observations on cortical bone thickness relative to medullary cavity[32], remnants of the epiphyseal line[31], enthesophytes, osteophytic bone formation around the edges of the joint surfaces and exostoses in the throcanteric fossa. As the methodology for adult age categorization (young vs mature adult) is not yet standardized, only adults with epiphyseal line visible were determined as young adults. Exostoses and enthesophytes were coded simply as absent or present[33,34]. Standard measurements were made with sliding caliper and osteometric board[29]. Sex estimation based on these measurements was difficult, since at the moment there is no known close reference population and only a few of the individuals from the site have been assigned belonging to certain populations[35]. As a summary (Table A-C in S4 Appendix), 39 femora of Levänluhta (LL) were investigated of which 38 were identified as left and 1 as right. In addition, 4 left femora from Käldamäki (KM) were studied. In addition, 19 animal bone samples were studied for the isotopic baseline (S5 Appendix).

### Radiocarbon and isotopic analyses

Radiocarbon and stable carbon and nitrogen isotope analyses were made on the cortical fraction of bone collagen at the Laboratory of Chronology, Finnish Museum of Natural History (Luomus), University of Helsinki (UH). The bones were sampled using a low-speed rotary drill equipped with a diamond-drill bit. The surface of the specimen was first drilled clean, and the sample powder was subsequently drilled from a cortical bone. In some cases, a fragment was detached instead of drilling. The samples were cleaned using ultrasonification in Milli-Q water, crushed and sieved to include the ≤500 μm fragments. The method for collagen extraction is based on the Longin method[36] and follows the protocol described in Berglund et al.[37] with some modifications: hydrolysis and carbonate removal was conducted in an ice bath using 10% HCl, and the final drying was done at 90–100°C. To check post-mortem alteration, the contents of N and C (wt-%) and the atomic C/N ratio of the collagen were monitored, discarding samples not meeting the commonly accepted quality criteria for well-preserved collagen. The acceptance range of C/N ratios characteristic for unaltered collagen was adopted as 2.9–3.6[38].

For radiocarbon analyses, the pretreated samples were mixed with a stoichiometric excess of CuO and packed into glass ampoules, which were pumped in a vacuum and torch-sealed. The packed samples were combusted at 520°C overnight. The released CO_2_ was collected and purified with liquid-N_2_ and ethanol traps at -196 ºC and -85ºC, respectively. After purifying and measuring the sample δ^13^C value with IRMS (Thermo Finnigan Delta Plus XL; in dual inlet mode) for fractionation correction, the CO_2_ samples were converted to graphite targets in the presence of zinc powder and an iron catalyst[39]. AMS measurements were eventually performed at the Uppsala Tandem Laboratory[40]. In addition, the radiocarbon ages of 5 samples were also measured at ETH Zurich. In these cases, the combined radiocarbon ages—if applicable—were used as a basis for analyses.

For the measurement of δ^13^C and δ^15^N values, approximately 80 μg and 250 μg, respectively, of dried and homogenized collagen powder was weighed into tin cups. The isotopic composition of carbon and nitrogen was measured at Luomus/UH by the EA-IRMS (Elemental Analyzer-IRMS; NC 2500 + Thermo Finnigan Delta Plus Advantage) method. All samples were run in duplicate. The isotopic values, normalized using international, certified isotopic reference materials IAEA-CH3, -CH7, -N1, -N2, USGS-40 and -41, are given in the delta (δ)-notation in parts per mille (‰), relative to the international standards VPDB (carbon) and AIR (nitrogen) following:

$$\delta =\left(R_{sample}/R_{standard}–1\right)\times 1000$$ (1)

where δ is either δ^13^C or δ^15^N and R is ^13^C/^12^C or ^15^N/^14^N for carbon and nitrogen, respectively. Typical reproducibility (1σ), estimated from repeated measurements of in-house reference material and sample replicates is ±0.15‰ and ±0.3‰ for carbon and nitrogen, respectively.

The isotopic values of the extracted collagens were additionally determined by the Unit of Geochemistry at the Geoscience Faculty of the University of Tübingen (UT), Germany, using EA-IRMS (Elemental analyzer-IRMS; NC 2500 + Thermo Quest Delta+XL). In Tübingen, the results were tied to the VPDB and AIR–scales using the certified reference materials USGS24 and IAEA 305A. The average difference between UH and UT data were -0.2 and 0.2 per mille for carbon and nitrogen, respectively. Eventually, the UH and UT isotopic results were averaged for adopted values.

### Dietary modellings

Dietary information based on the measured isotopic values was obtained through FRUITS software[25]. The Iron Age diet was assumed to consist of three food groups: terrestrial resources (TR) (including plants, animals, and dairy products), freshwater animals (FA), and marine animals (MA). Due to non-separable isotopic signatures, a more detailed grouping was not reasonable. The dietary modeling does not separate terrestrial plants from animals or hunter-gatherer subsistence from agriculture/cultivation. The nutrient data of National Food Composition Database of Finland, FINELI (https://fineli.fi/fineli/en/index) and/or of National Nutrient Database, United States Department of Agriculture (https://fdc.nal.usda.gov/) provided the carbon dry-weight compositions of each assumed dietary item. These were averaged (±standard error of the mean) for each food group (Table K in S6 Appendix) and used in dietary modelings. Fractional isotopic values (Table J in S5 Appendix) of the edible macronutrients—protein and energy (carbohydrates + fats)—for each food group were obtained based on measured isotopic data from literature through the public δIANA database[41] established for paleodietary research in the Nordic areas, supplemented with the data of this work. All the isotopic values (plants, dairy products, flesh, and bone; Table G-H in S5 Appendix) were individually converted to macronutrient isotopic values by adopting case-specific offsets (Table I in S5 Appendix) from literature. These were then averaged (±standard error of the mean) for each food group. Finally, FRUITS modelings were conducted separately for each sample k, i.e. isotopic value pair, and as a result, the relative contributions of each food group toward the isotopic signals (θ_i,k_; i = FA or MA) and the food group intakes (α_i,k_) for that individual were obtained (Table L-M in S6 Appendix). It is expected that the assumed isotopic baseline and macronutrient concentrations remained constant through the studied era. No *a priori* assumptions were made in dietary modelings as they were noticed to significantly affect the results.

The dietary isotopic baseline within the Baltic Sea environment depends on the location from which the dietary carbon was obtained. Therefore, modeling was performed by assuming three scenarios concerning the origin of the marine carbon: being solely from the Bothnian Bay (henceforth: *Bothnia*, latitudes 63–66°N), from the Baltic Sea beyond Kvarken (*Baltic*, latitudes 56–63°N), or from both regions (*total*, latitudes 56–66°N). The Baltic region corresponds to an area from Öland (latitude 56°N) to the end of the Bothnian Bay (lat. 66°N). This division is based on the measured carbon stable isotopic data on dissolved inorganic carbon of the Baltic Sea[42]—the isoscape of Bothnian Bay differs from the rest of the Baltic Sea, the transition being geographically located around Kvarken (lat. 63°N).

### Corrections adopted for radiocarbon data

Marine reservoir effect (MRE) corrections depend on the assumed location from which the dietary carbon is routed into the individuals. Thus, *Bothnia*, *Baltic*, and *total* scenarios were also assumed for the MRE corrections. Terrestrial isotopic baselines were assumed to be similar in the Ostrobothnia and Baltic regions and freshwater sources were considered local.

MRE of the Baltic Sea is essentially linked to salinity[43] as this reflects the input of saline oceanic water through Danish Straits. Therefore, MREs were estimated for all the scenarios by evaluating the average salinities of the regions by using HELCOM salinity data (http://ocean.ices.dk/helcom; ~570000 salinity measurements) and the salinity-MRE relations estimated by Lougheed et al.[43]. The measured salinity data were sampled randomly and equidistantly (in terms of latitude), the obtained sampled salinities (14399 samplings for *Baltic*, 5662 samplings for *Bothnia*, and 20000 in total) were converted to MRE values and the obtained MREs were averaged separately for all scenarios. For *Bothnia*, the maximal marine reservoir effect was obtained as *MRE*_*max*, *Bothnia*_ = 76(46) ^14^C years. As the marine flavor increases southward within the Baltic Sea basin due to marine water inflow through the Danish Straits, the salinity increases too, and thus we correspondingly obtained *MRE*_*max*, *Baltic*_ = 207(43) ^14^C years. For the total scenario, *MRE*_*max*, *total*_ = 180(63) was obtained. The uncertainties were estimated as one standard deviation and are given in parentheses. Two measurements for the shallow strait of Kvarken[43] provide an estimate of *MRE*_*max*, *Kvarken*_ = 115(50) ^14^C years, being consistently in between the adopted *MRE*_*max*, *Bothnia*_ and *MRE*_*max*, *Baltic*_.

The maximal freshwater reservoir effect (*FRE*_*max*_) was obtained by measuring the ^14^C contents of modern freshwater fish samples of *Sander lucioperca* (pike-perch) and *Rutilus rutilus* (roach) from the Kyrö river and following the method of Philippsen et al.[44]. Flesh samples of freshwater fish were not chemically pretreated before drying. This selection was made to mimic the ^14^C contents of fish eventually contributing to formation of ^14^C content in the human bone collagen samples. Two modern freshwater fish samples (caught 10/2012) yielded radiocarbon contents of *pMC*_*FA*,*pike-perch*_ = 102.5(3) (Hela-3550) and *pMC*_*FA*,*roach*_ = 102.2(3) (Hela-3552). The corresponding atmospheric ^14^C concentration was estimated as an weighed average of measured[45] and extrapolated atmospheric ^14^C concentrations during the sample formation, corresponding to the living years of fish, here 7 (pike-perch) and 5 (roach) years. At first, annual weight increments of fish were estimated according to a Bayesian length–weight relationship with species-specific parameters[46] (www.fishbase.org). These increments were used as weights in averaging the atmospheric ^14^C concentrations of the summer months of the growth years to obtain atmospheric reference values (*pMC*_*atm*_), for which uncertainties were adopted as 0.5 pMC units. *FRE*_*max*_ values were eventually estimated for each fish *i* from the ratio of the atmospheric and fish ^14^C concentrations as follows:

$${FRE}_{max,i}=8033∙\ln\frac{{pMC}_{atm}}{{pMC}_{FA,i}}$$ (2)

where 8033 is the “Libby” mean lifetime of ^14^C. The uncertainty of *FRE*_*max*,*i*_ was obtained through error propagation as:

$${\Delta FRE}_{max,i}=8033∙\sqrt{{\left(\frac{{\Delta pMC}_{atm}}{{pMC}_{atm}}\right)}^{2}+{\left(\frac{{\Delta pMC}_{FA,i}}{{pMC}_{FA,i}}\right)}^{2}}$$ (3)

The *FRE*_*max*,*i*_ values were averaged to obtain an estimate for the maximum (average) freshwater reservoir effect of *FRE*_*Kyrö*_ = 107(45) ^14^C years, for which the uncertainty has adopted either the standard deviation or the individual Δ*FRE*_*max*,*i*_ as a maximum value.

Dietary modelling by FRUITS was used to estimate the relative contribution of each food group *i* toward the carbon isotopic signals for each sample k (θ_i,k_; *i* = FA or MA). The marine and freshwater reservoir effect corrections for radiocarbon ages were then obtained by scaling down the MRE and FRE, respectively, with the average of these fractions modelled for carbon isotopic ratios:

$${MRE}_{k}=\theta_{MA,C,k}∙{MRE}_{max}$$ (4)

$${FRE}_{k}=\theta_{FA,C,k}∙{FRE}_{max}$$ (5)

Eventually, the corrected radiocarbon ages *RA*_*k*,*corr*_ were obtained from original ages (*RA*_*k*_) as:

$${RA}_{k,corr}={RA}_{k}-{MRE}_{k}-{FRE}_{k}$$ (6)

and the uncertainties were obtained by quadratic summing through error propagation. The corrected ages were calibrated with the Oxcal 4.3. software[26] by using IntCal13 calibration curve[47] to obtain calendar-year probability distributions (*cpd*; Table N in S8 Appendix). As the sample material was collagen from cortical bone, an own age offset of 18 ± 5 years[48] was eventually added to individual *cpd*s to reflect the moment of burial of the individuals, except for the identified children.

### Clustering, time series, and statistical analyses

Hierarchical cluster analyses were performed (S4 Appendix) on the isotopic data to identify its subgroups. A group-average clustering procedure was used for this, as it takes into account all the pairwise distances within the observed clusters to evaluate inter-cluster distances. This was accompanied by a squared Euclidean distance estimate due to its intrinsic power to separate clusters—it provides the highest inter-cluster distances and low within-cluster distance. Comparisons between the isotopic data clusters and subsets were performed by using a two-sample T test by assuming unequal variances (Table F in S4 Appendix). This selection is based on an assumption of normally distributed data and statistical analyses of small sample sets[49]. Simple moving averages (Table N in S8 Appendix) with a 50-year window were used to visualize the temporal dependence of the number of dates, the isotopic ratios, their standard deviations and the intakes of food groups (α_i_). Time series were plotted as a function of the mean values of the *cpd*s of the RE-corrected dates.

Chronological phase analyses were conducted through Bayesian modellings with Oxcal on the reservoir-effect corrected dates (S7 Appendix). For outliers, were adopted through the General outlier model with the basic settings recommended[50]. All the phase boundaries (Boundary-option in Oxcal) provided by the models were shifted, as above, to account for the bone own age. The phase boundaries are given as Highest Posterior Density (HPD, 1 and 2σ) ranges, mean values, and their standard deviations (Table 4). In addition, the kernel density estimation (KDE) approach[27] was used to assess the widths and shapes of the summed calendar-year probability distributions (*scpd)*, their uncertainties and sensitivities toward the origin of carbon, and subsequent RE corrections (Fig M in S7 Appendix). The KDE approach was considered suitable since dependence[27] between the samples is established by the peculiar habit of water burial. In addition to the KDE approach, total (*scpd*_LL_) and cluster-specific (*scpd*_LL1-LL3_) *scpd*s of the RE-corrected dates were used to visualize the development of the intensity of the burial activity. Randomly distributed calendar year dates (30 pcs) were assumed to span over AD 320–780 to form simulated (R_Simulate in Oxcal) ^14^C dates and their sum distribution. This was repeated 30 times to obtain average sum distribution *scpd*_Random_ representing a null hypothesis of constant human activity within the period. The uncertainty of the simulated random sum distribution (Δ*scpd*_Random_) was obtained as a standard deviation (1σ) of the simulated distributions. The eventual *scpd*_LL-Random_ reflecting the periods of time of less/more-than-constant human activity was obtained by subtracting *scpd*_Random_ from the total distribution *scpd*_LL_. The one-sigma limits were deduced as *scpd*_LL-Random_ ± *Δscpd*_Random_. The KDE and background-subtraction approaches provided nearly similar outcomes concerning the temporal development, while cluster-specific approach provided insight on the roles of the observed subgroups.

## Results

### Diverse isotopic distribution

**Fig 2:**
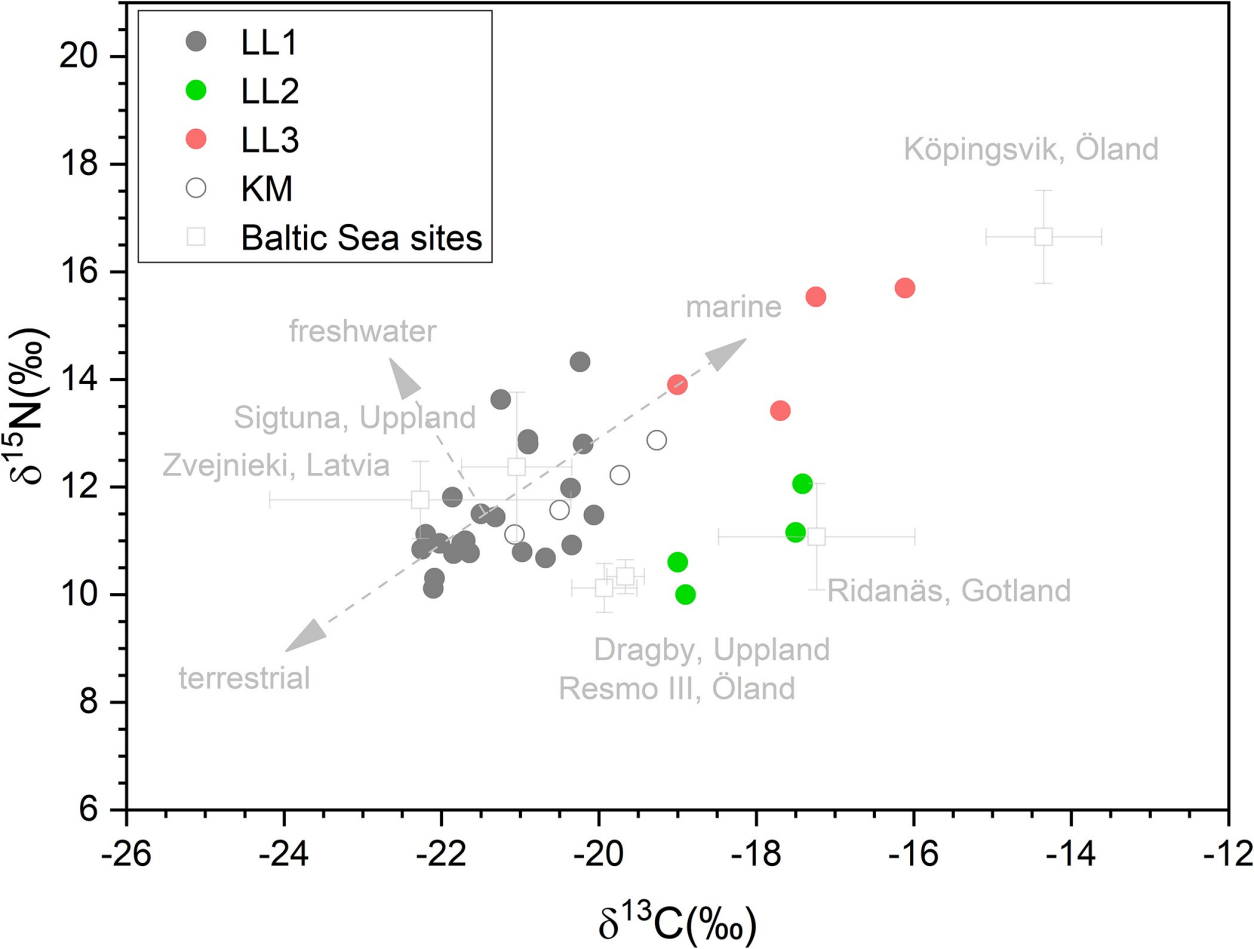
Carbon and nitrogen stable isotopic ratios. The measured stable isotopic ratios (Table 1) of Levänluhta (LL; full circles) and Käldamäki (KM, open circles) are presented among the reference data from the Baltic basin. LL1, 2 and 3 refer to the subgroups revealed by hierarchical clustering analysis. The obtained stable isotopic data are compared to the data sets (see text; Table D in S4 Appendix) of human bone collagen[52–55] shown by light grey squares and corresponding population standard deviations. The general direction of influence of the food groups are schematically given as grey arrows, the origin being the average value of the LL isotopic data.

Extraordinarily broad carbon (C) and nitrogen (N) isotopic ratio distribution is observed in the human bone collagen of the Levänluhta individuals (Table 1, Fig 2, Table D in S4 Appendix). The full δ^13^C and δ^15^N distributions have standard deviations of up to 7 and 5 times, respectively, compared to those of the reference populations around the Baltic Sea. Half (50%) of the individuals are osteologically identified as adults (Table B in S4 Appendix), but commingled skeletal remains allow for sex estimations for only 24% of individuals[20,21] (Text and Table C in S4 Appendix). Nevertheless, a picture emerges of a burial containing children and adults of both sexes, but with clear emphasis on adult females. The broad isotopic distribution involves also the identified female subgroup (Text and Fig H in S4 Appendix), this being consistent with the expected female dominance within the Levänluhta material. Since the isotopic differences between females and males are of the order of 1 permille[51], the broad distribution likely indicates significant differences among individuals in the importance of terrestrial, freshwater, and marine resources in their diets and, consequently, highly versatile subsistence strategies.

**Table 1:** Human bone collagen isotopic data.

Site	Sample #	C-%	N-%	C/N value	δ^13^C(‰), UH	δ^15^N(‰), UH	δ^13^C(‰), UT	δ^15^N(‰), UT	δ^13^C(‰), adopted	δ^15^N(‰), adopted	subgroup
LL	1	39.3	12.8	3.3	-20.2	12.8			-20.2	12.8	1
LL	2	33.9	12.8	3.6	-20.9	12.8			-20.9	12.8	1
LL	3	38.3	11	3.4	-21.7	11.0			-21.7	11.0	1
LL	6	37.1	10.6	3.5	-19.0	10.6			-19.0	10.6	2
LL	10	38.3	12.8	3.6	-20.9	12.8			-20.9	12.8	1
LL	11	39.1	11.5	3.6	-21.5	11.5			-21.5	11.5	1
LL	12	40.0	10	3.6	-18.9	10.0			-18.9	10.0	2
LL	13	39.1	14.0	3.3	NA	NA	-17.4	12.1	-17.4	12.1	2
LL	14	37.5	13.3	3.3	-16.3	15.6	-15.9	15.8	-16.1	15.7	3
LL	15	38.6	14.0	3.2	-16.7	16.3	-17.7	14.8	-17.2	15.5	3
LL	16	41.0	14.6	3.3	-22.4	10.6	-21.8	10.0	-22.1	10.3	1
LL	19	40.1	14.4	3.2	-21.5	11.6	-21.1	11.3	-21.3	11.4	1
LL	20	40.7	14.4	3.3	-20.4	11.5	-19.8	11.4	-20.1	11.5	1
LL	21	41.7	14.8	3.3	-19.2	14.0	-18.8	13.8	-19.0	13.9	3
LL	22	40.8	14.5	3.3	-22.3	10.9	-21.8	11.0	-22.0	11.0	1
LL	23	40.2	14.0	3.4	-22.4	11.1	-22.0	11.1	-22.2	11.1	1
LL	24	40.0	14.1	3.3	-22.3	10.2	-21.9	10.0	-22.1	10.1	1
LL	25	38.8	13.7	3.3	-21.1	13.0	-20.8	12.7	-20.9	12.9	1
LL	26	42.0	15.0	3.3	-21.8	10.9	-21.5	10.7	-21.6	10.8	1
LL	27	42.9	15.3	3.3	-17.8	13.7	-17.6	13.2	-17.7	13.4	3
LL	29	40.0	14.3	3.3	-21.1	11.0	-20.9	10.6	-21.0	10.8	1
LL	30	38.8	13.9	3.2	-22.0	10.8	-21.7	10.7	-21.8	10.8	1
LL	31	38.7	13.8	3.3	-20.4	11.1	-20.3	10.7	-20.3	10.9	1
LL	33	42.7	15.1	3.3	-21.4	13.7	-21.1	13.5	-21.2	13.6	1
LL	34	42.4	14.9	3.3	-20.4	14.5	-20.1	14.2	-20.2	14.3	1
LL	35	41.2	14.6	3.3	-20.9	10.7	-20.5	10.7	-20.7	10.7	1
LL	36	40.6	14.3	3.3	-22.0	11.8	-21.7	11.8	-21.9	11.8	1
LL	37	39.0	13.9	3.3	-21.8	11.1	-21.7	10.8	-21.7	11.0	1
LL	38	39.1	13.8	3.3	-17.4	11.2	-17.6	11.1	-17.5	11.2	2
LL	39	40.6	14.3	3.3	-20.4	12.0	-20.3	11.9	-20.4	12.0	1
KM	40	39.0	13.8	3.3	-21.2	11.1	-21.0	11.1	-21.1	11.1	
KM	41	43.0	14.9	3.4	-19.4	12.7	-19.1	13.0	-19.3	12.9	
KM	42	41.0	14.3	3.3	-20.8	11.5	-20.2	11.7	-20.5	11.6	
KM	43	38.3	13.6	3.3	-19.8	12.1	-19.7	12.3	-19.7	12.2	

Human bone collagen isotopic data measured within this study. LL = Levänluhta, KM = Käldamäki, UH = University of Helsinki, UT = University of Tübingen, subgroup = number of the subgroup to which the individual belongs (LL1-LL3). The following results were rejected (struck through) from the dietary modelling and subsequent analysis based on identification, quality criteria or radiocarbon dating. #4: radiocarbon age too young (139 ± 30 BP); #5: right femur; #7: high C/N value; #8,9: C-% not measured; #17: too small sample; #18: radiocarbon age too old (4124 ± 34 BP); #28: C-%, N-% not measured, #32: risk of being duplicate. The adopted isotopic data were obtained by averaging of the UH and UT results. Altogether, there were 30 and 4 acceptable measurements on left *femora* of Levänluhta and Käldamäki individuals, respectively.

Hierarchical cluster analysis reveals three subgroups (LL1-LL3) with distinguishable isotopic signatures (Fig 2, Text and Table E in S4 Appendix). Under assumption of normally distributed data, this heterogeneity can be statistically verified and elementally decomposed (Table F in S4 Appendix). The subgroup LL1 contains most of the individuals (22/30) and forms the base population within the Levänluhta site. Its isotopic distribution essentially resembles that of medieval Sigtuna[52] and, somewhat, the hunter-gatherers of Zvejnieki[54] with smallest squared-Euclidean(SE) distances. The subgroup LL2 has the smallest SE distance to the Viking Age population of Ridanäs/Gotland, having a mixed terrestrial and marine diet[53]. Its separation from LL1 is solely due to the significantly different carbon isotopic ratios (t(4) = -6.675, p = 0.003) with separation of about 3‰. The subgroup LL3 is separated from LL1 (δ^13^C: t(3) = -6.077, p = 0.009 and δ^15^N: t(4) = -5.367, p = 0.006) and LL2 (δ^15^N: (t(6) = -5.111, p = 0.002) with the largest observed SE distances within the Levänluhta site. The individuals from the other water burial of Käldamäki isotopically resemble those of LL1 and Sigtuna but possess slightly higher δ^13^C values.

**Fig 3:**
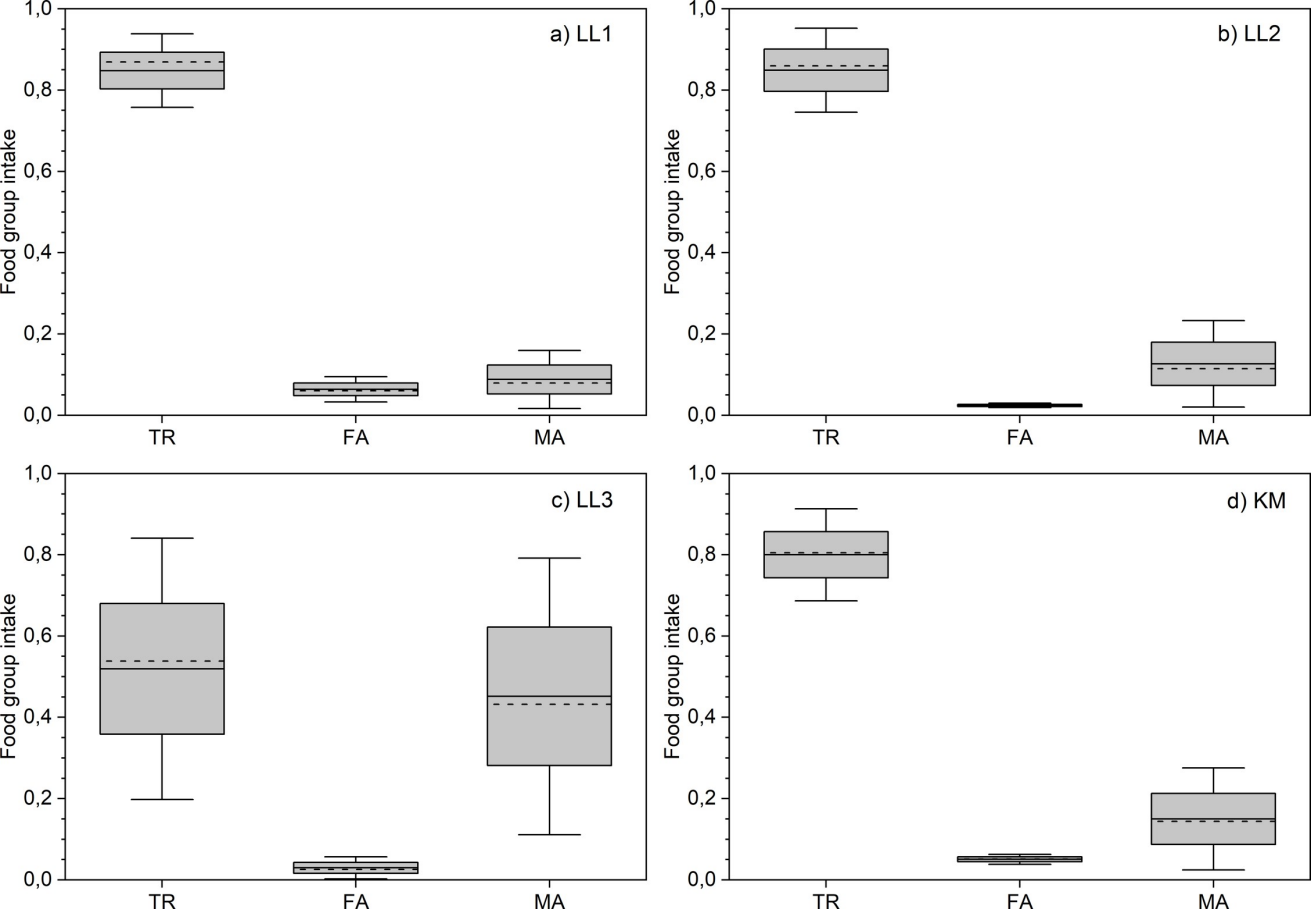
Subgroup-specific food group intake distributions. Solid lines represent the mean values of the subgroup-specific food group intake distributions and the dashed lines represent the median values obtained by the FRUITS dietary reconstructions by assuming the *Bothnia* scenario. The boxes and whiskers illustrate one and two standard deviations of the distributions. The illustrated data is coherent with the Table 2.

Conversion of isotopic data into food group intakes[25] provides insight into the above and emphasizes the versatility of the livelihoods utilized (Fig 3, Table 2, Tables L-M in S6 Appendix). Terrestrial resources provided the major portion (80–85%) of dietary input for most of the subgroups, except for LL3 that shows very large average marine intake of 36–49%. Overall, the freshwater component of less than 8% reflects a modest dietary supplement from freshwater sources, probably reflecting the scarcity of resources (Kyrö river) compared to the lake region in eastern Fennoscandia (Fig 1). Particularly, the modeled (*Bothnia*) diet of LL1 is 85% terrestrial with a limited use of marine (9%) and freshwater (6%) food. Usage of all the available dietary resources is consistent with the interpretation of the LL1 individuals as belonging to a local base population.

**Table 2:** Mean subgroup-specific food group intakes.

Subgroup	N	Area	Terrestrialα_TR_	Freshwaterα_FA_	Marine α_MA_	Protein β_Protein_	Energy β_Energy_
LL1	22	*Bothnia*	0.85	0.06	0.09	0.47	0.53
		*Baltic*	0.86	0.07	0.07	0.47	0.53
		*Total*	0.85	0.07	0.08	0.47	0.53
LL2	4	*Bothnia*	0.85	0.02	0.13	0.44	0.56
		*Baltic*	0.82	0.02	0.16	0.46	0.54
		*Total*	0.83	0.02	0.15	0.45	0.55
LL3	4	*Bothnia*	0.48	0.03	0.49	0.57	0.43
		*Baltic*	0.58	0.06	0.36	0.53	0.47
		*Total*	0.54	0.04	0.42	0.55	0.45
KM	4	*Bothnia*	0.80	0.05	0.15	0.48	0.52
		*Baltic*	0.81	0.06	0.13	0.48	0.52
		*Total*	0.80	0.05	0.14	0.48	0.52

The mean subgroup-specific food group intakes (α_k_, k = TR, FA, MA) have been obtained from dietary modeling together with the estimated protein and energy fractions (β_f_, f = Protein, Energy) of food. The values assume either Bothnian Bay (63–66°N i.e. *Bothnia*), Baltic Sea from Öland to Kvarken (56–63°N i.e. *Baltic*), or both (56–66°N i.e. *total*) as the area of origin for the marine dietary input. LL = Levänluhta, KM = Käldamäki, N = number of individuals within the subgroup (N_all_ = 34, N_LL_ = 30).

The people of the small subgroups, LL2, KM, and particularly LL3, consumed larger amounts (13–49%) of marine food. The larger marine fraction (15%) of the KM subgroup appears logical due to the closer vicinity of Käldamäki to the seashore (Fig 1, Fig F in S2 Appendix). The use of freshwater food in KM is non-negligible (5%), emphasizing the importance of the freshwater component even in close vicinity to the marine shoreline. The LL2 individuals, instead, represent a significant exception compared to LL1 and KM: they essentially did not use freshwater resources at all. The diet of LL2 was a mixture of marine (13–16%) and terrestrial (82–85%) food, reflected by the elevated average δ^13^C value of -18.5‰ and leading to the observed small SE distance to the population of Ridanäs, Gotland.

Irrespective of the assumed source area for the marine dietary input (*Bothnia*, *Baltic* or *total*; Table 2, Tables L-M in S6 Appendix), the modeled portions of either freshwater, marine or terrestrial diets remain essentially unchanged, except for the half-marine LL3 individuals. The shallow strait of Kvarken (Fig 1) divides the Gulf of Bothnia into the Bothnian Sea and more northern and brackish Bothnian Bay. Aquatic fauna of Bothnian Bay contains carbon originating from organic matter of riverine runoff waters that are typically dominated by dissolved or particulate carbon with low isotopic ratios[56]. Thus, the carbon isotopic ratio of dissolved inorganic carbon in the Bothnian Bay differs from that of the Bothnian Sea and Baltic proper[42]. As a result, a large difference in isotopic baseline values is observed (Table J in S5 Appendix) between the MA_Bothnia_ (δ^13^C_MA,Bothnia, protein_ = -20.0‰) and the MA_Baltic_ (δ^13^C_MA,Baltic, protein_ = -15.9‰) food group scenarios, affecting the model output accordingly for LL3.

The observed strong terrestrial dietary component may be partly due to agricultural traditions (S2 Appendix). In Ostrobothnia, the gradually extending shoreline meadows provided fodder for farmyard animals, whereas higher elevations were already used for cultivation from the Late Bronze Age[17] to the pre-Roman Iron Age[57]. Field cultivation in Ostrobothnia is proven at around AD 600[58] paralleling the observation of permanent and manured cultivation at 64°N latitude in Northern Sweden from around AD 480 onward[59], also located 30 km inland. In the vicinity of Levänluhta, the first sporadic occurrences of *Hordeum* (barley) and *Secale* (rye) pollen are observed from around AD 690 onward[22]. The recent study of Levänluhta and Käldamäki burials[22] proves contemporaneous animal husbandry within the area, as bones of domesticated animals were found. Thus, there are inarguable signs of agriculture within the area.

The large terrestrial portion could also reflect hunting and gathering. Indeed, the estimated high protein usage (Table 2) generally resemble those of northern hunter-gatherer diets[60], strongly based on animal protein. Hunting and subsequent fur trading have traditionally been important sources of prosperity in prehistoric Northern Europe[61,62]. The Suomenselkä ridgeline (Fig 1) is a roughly 400 km long moraine formation that acts as the watershed from which all the Ostrobothnian rivers, including the Kyrö river, originate. It separates Ostrobothnia from the eastern lake region within Eastern Fennoscandia. Along this ridgeline, massive trapping-pit systems have been found[17], evidencing the importance of past hunting. In Ostrobothnia, large numbers of arrowheads deriving from the Migration period (AD 400–550) provide additional evidence for hunting[16], contemporaneous to the Levänluhta era. The proximity of the Levänluhta site to the southern end of the Suomenselkä ridgeline provided people access to vast eastern and northern hunting grounds. In Levänluhta, we see inland people, particularly the LL1 subgroup, pursuing strongly terrestrial livelihoods with high protein consumption for centuries. These people were likely instrumental in hunting and subsequent fur trading activities, thus contributing in the evolution of the Ostrobothnian prosperity during the Iron Age.

Additionally, seal hunting (S1 Appendix) was already an important source of livelihood for both sides of the Gulf of Bothnia from the Mesolithic[18,63,64]. The worldwide importance of seal hunting circa AD 300 is visible through the ancient Roman price list Edict of Diocletian[63,65,66]: skins of seals were the most expensive ones. Seal hunting has been important even closer to the modern era. In the 16^th^ century, typically 55–75% of peasants in Ostrobothnia participated in massive seal hunting expeditions lasting several months[63]. Based on these thousands of years of traditions, seal hunting likely provided a significant component of the marine dietary influence observed, naturally accompanied by fishing.

### Levänluhta timeline

**Fig 4:**
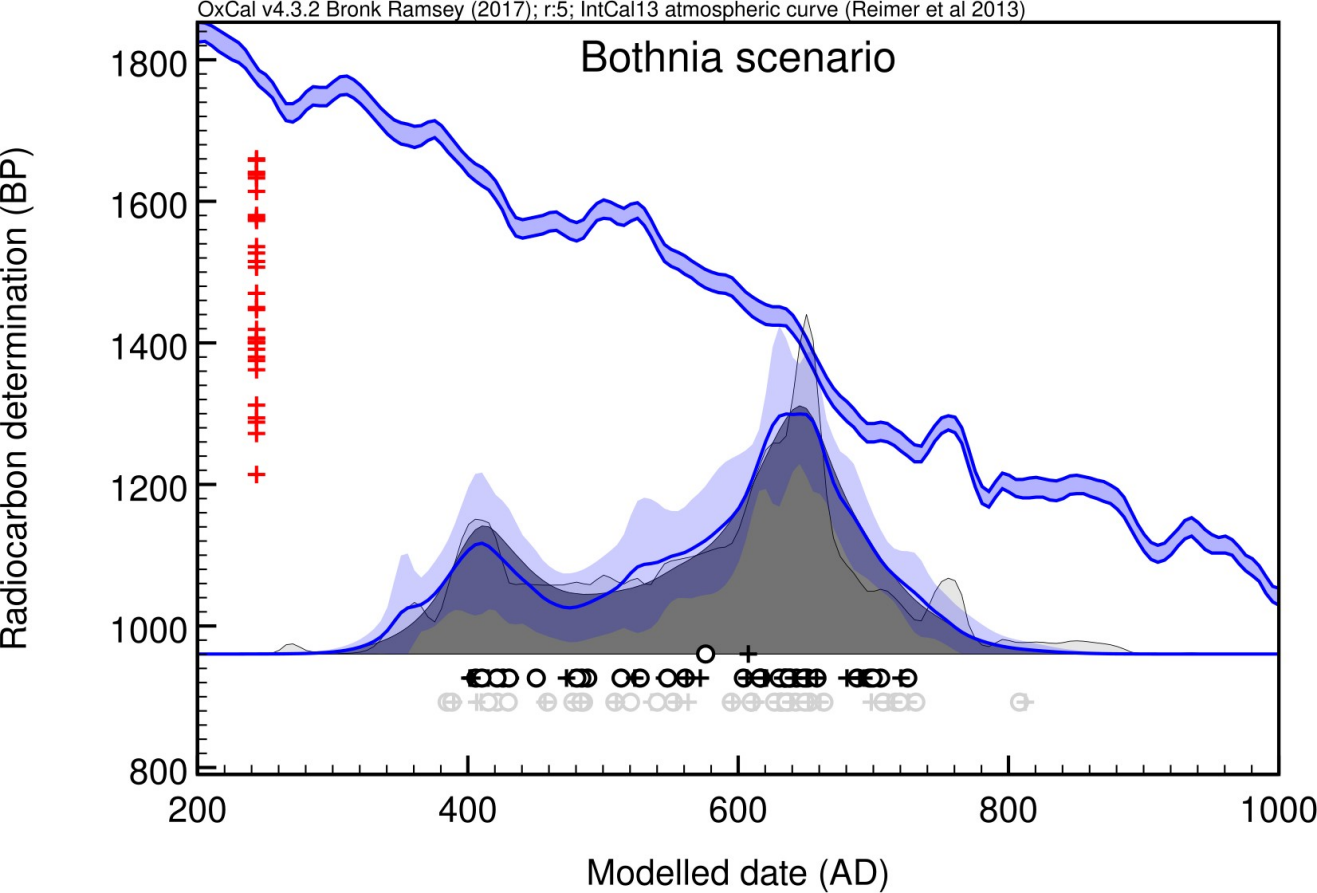
Kernel density analysis of the summed calendar-year probability distribution. Indicates burial density based on reservoir-effect corrected Levänluhta dates of 30 individuals (Table 3) obtained through kernel density estimate (KDE) analysis[27] and by assuming Bothnian Bay as the source of marine carbon. Red crosses: individual radiocarbon dates; open dark grey circles: medians of the posterior calendar-year probability distributions; open light grey circles: medians of the individual *cpd*s; dark grey distribution: summed posterior calendar-year probability distribution obtained by KDE analysis; blue line and blue overlying band: mean ± 1σ confidence limit of the produced KDE distribution. This indicates the significance of the observed features.

Quantitative understanding of dietary habits allows for estimating the influences of both marine and freshwater reservoir effects within the measured radiocarbon dates. Archaeologically, the Levänluhta water burial has been estimated to span from AD 300 to AD 800[19,22]. Radiocarbon analyses and reservoir-effect corrections based on isotopic evidence (Table 3), and subsequent Bayesian chronological modelings (Table 4, S7 Appendix) prove this long human timeline ranging through centuries. Particularly, the kernel density estimation (KDE)–based summed calendar-year probability distribution (*scpd*) continues from AD 300 to AD 800 and displays peaked burial activities at about AD 400 and AD 650 (Fig 4). According to the confidence limit estimates, the latter peak particularly stands out from the distribution.

**Table 3:** Radiocarbon ages and their corrections.

Site	Sample #, k	Lab code (Hela-xxxx, ETH-xxxxx)	RA (BP)	σ	RA_adopted_ (BP)	σ_adopted_	MRE_k_ (^14^C yr)	σ_MRE_	FRE_k_ (^14^C yr)	σ_FRE_	RA_corr_ (BP)	σ_corr_
LL	1	2128	1339	30	1339	30	17	12	10	8	1312	33
LL	2	2243	1473	30	1473	30	13	10	13	10	1447	33
LL	3	2244	1675	30	1675	30	6	6	9	7	1660	31
LL	6	2251	1376	30	1376	30	10	8	4	4	1362	31
LL	10	2262	1433	30	1433	30	13	10	13	10	1407	33
LL	11	2263	1633	30	1633	30	8	7	11	8	1614	32
LL	12	2264	1547	30	1547	30	7	7	4	4	1536	31
LL	13	3268	1423	30	1423	30	19	14	3	4	1401	33
LL	14	3269	1630	31	1630	31	57	36	3	3	1570	47
LL	15	3270	1626	31	1626	31	48	30	4	4	1574	43
LL	16	3271	1539	31	1539	31	5	5	7	6	1527	32
LL	19	3274	1664	33	1599	66	8	7	10	8	1580	66
LL	19	57297	1533	26								
LL	20	3275	1232	30	1232	30	11	9	7	6	1214	32
LL	21	3276	1441	31	1441	31	27	18	7	7	1406	37
LL	22	3277	1309	31	1287	31	6	6	10	7	1272	32
LL	22	57298	1265	26								
LL	23	3278	1654	29	1654	29	6	5	10	8	1638	31
LL	24	3279	1481	28	1481	28	5	5	7	6	1470	29
LL	25	3280	1402	28	1402	28	13	10	14	10	1375	31
LL	26	3281	2288	29	1414	28	6	6	8	7	1400	29
LL	26	55271	1414	28								
LL	27	3282	1691	28	1691	28	29	19	4	4	1658	34
LL	29	3284	1532	28	1523	28	7	7	8	7	1507	30
LL	29	57299	1513	26								
LL	30	3285	1405	29	1405	29	6	6	9	7	1391	30
LL	31	3286	1530	27	1530	27	9	8	6	6	1515	29
LL	33	3288	1466	27	1466	27	6	5	10	8	1450	29
LL	34	3289	1439	28	1439	28	9	8	11	8	1419	30
LL	35	3290	1648	27	1648	27	8	7	7	6	1633	29
LL	36	3291	1314	27	1314	27	8	7	12	9	1294	29
LL	37	3292	1656	28	1656	28	6	6	9	7	1641	29
LL	38	3293	1304	27	1304	27	12	10	4	4	1288	29
LL	39	3294	1401	27	1401	27	13	10	9	7	1380	30
KM	40	3295	1470	27	1470	27	8	7	8	7	1453	29
KM	41	3296	1581	27	1581	27	21	15	7	6	1553	31
KM	42	3297	1596	27	1596	27	11	9	8	7	1577	29
KM	43	3298	1663	28	1663	28	17	12	7	6	1640	31

Radiocarbon ages (RA) measured within this study. LL = Levänluhta, KM = Käldamäki, Hela-xxxx = four-digit code for the University of Helsinki ^14^C dates, ETH-xxxxx = five-digit code for ETH Zürich ^14^C dates, σ’s = standard deviations of ^14^C dates, MRE = marine reservoir effect correction, FRE = freshwater reservoir effect correction, σ_yy_’s = uncertainties (of MRE, FRE or corrected age). The following results were rejected from the subsequent analysis based on quality criteria or radiocarbon dating. #4: radiocarbon age too young (139 ± 30 BP, the color of bone was also whiter); #5: right femur; #7: high C/N value; #8,9: C-% not measured; #17: too small sample; #18: radiocarbon age too old (4124 ± 34 BP); #28: C-%, N-% not measured, #32: risk of being duplicate. The following samples were measured also through University of Tübingen & ETH Zürich, since verification was needed based on technical indications: #19, 22, 26, 29, 32. In the cases when adopted ^14^C age was determined as an average of these measurements (#19, 22, 29, 32), adopted uncertainties were deduced as either statistical uncertainties of the individual measurements or as│RA_Hela_−RA_ETH_│/2, whichever was highest. The Bothnian Bay scenario as the origin of marine carbon was assumed for all the samples. Altogether, there were 30 and 4 acceptable measurements of Levänluhta and Käldamäki individuals, respectively.

**Table 4:** Results of the Bayesian chronological models.

Event	68% HPD, early	68% HPD, late	95% HPD, early	95% HPD, late	mean	σ	median
LL start, Bothnia	385	425	345	465	400	25	405
LL start, Baltic	405	530	390	540	475	45	490
LL start, total	405	530	385	540	470	45	485
LL start, archaeology*							300
LL end, Bothnia	715	775	700	810	750	30	750
LL end, Baltic	720	775	705	815	755	30	750
LL end, total	715	770	700	810	750	30	750
LL end, archaeology*							800

Quantitative results of the Bayesian chronological models for the Levänluhta burial given as calendar year estimates. HPD = highest posterior density, σ = standard deviation of calendar-year probability distribution provided by the calibration. The boundaries include the cortical bone own age correction of 18 ± 5 years[48] and thus the values reflect the moments when the burial activity has started/ended.

*Archaeological datings, assumed to correspond to the median ages, were adopted from the literatur [19,22].

The increase of *scpd* during the AD 300s reflects the start of the Levänluhta burial activity during the Late Roman Iron Age. The subsequent peak can be linked to the Southern Ostrobothnian culture flourishing, particularly during the Migration period of AD 400–550 when the area became the richest within the Eastern Fennoscandia[19]. Bayesian modeling yields the start boundary (median) of the burial activity as AD 405. The latter peak coincides with the earliest interpretations of the site dating (AD 600–650)[19] based on large numbers of Merovingian artifacts found. This emphasizes the increasing importance of the Levänluhta, particularly during the Merovingian period. Toward AD 800 the burial activity declines and the end boundary (median) is modeled as AD 750. The decline in the burial activity within circa AD 700–800 is coherent with the general decline[19] of the inhabitation within Ostrobothnia. This eventual loss has been explained as a consequence[18] of the collapse of the North Sea trading network in around AD 800[67].

### Unforeseen climatic downturn hit the population

Folktales have been told throughout the Northern Hemisphere on extended cold periods. These include the three-year Fimbulvinter in Scandinavia[12], the three-year loss of Sun in Buryatia[68] and the theft of the Sun and Moon in Finnic regions[69] (Table O in S9 Appendix). The geographical spread of the tales of the lost Sun is coherent with the hemispheric-scale tree-growth decline during the mid-6^th^ century AD[2] and the recently proven extensive loss of light of several years during the AD 540s[8] (S1 Appendix). If the cold period of Fimbulvinter has its counterpart in reality, the years of AD 541–544 would provide its explanation in terms of length, magnitude, and even its mid-millennial timing.

Less intensive volcanic analogies of the 17^th^ century AD caused significant crop losses, acute food shortages, and human struggle, which eventually yielded severely increased mortality in Eastern Fennoscandia and particularly in Ostrobothnia[10] (S1 Appendix). If the terrestrial isotopic signal observed in Levänluhta is strong due to cultivation, one would expect multitude of potential effects in the data due to the climatic downturn. Post-anomalous decline in the terrestrial food group intake would probably appear due to crop losses. Further, the subgroup-specific burial intensity estimates by *scpd*s would probably be affected subsequent to the downturn. Long-lasting starvation and malnutrition could, in principle, be also seen in isotopic ratios as an increase of δ^15^N values in human tissues due to catabolism and a slight decrease of δ^13^C values due to the recycling of body fat with distinctly low carbon isotopic ratios [70,71] although slow turnover rate of cortical bone could possibly surpass these effects. On the other hand, a strong role of cultivation practices could have elevated the δ^15^N values through manuring[72–74] exercised already at high latitudes[59]. Cattle manure could have been used to enhance crop yields thus elevating the plant δ^15^N values that were transferred into bone collagen through diet.

**Fig 5:**
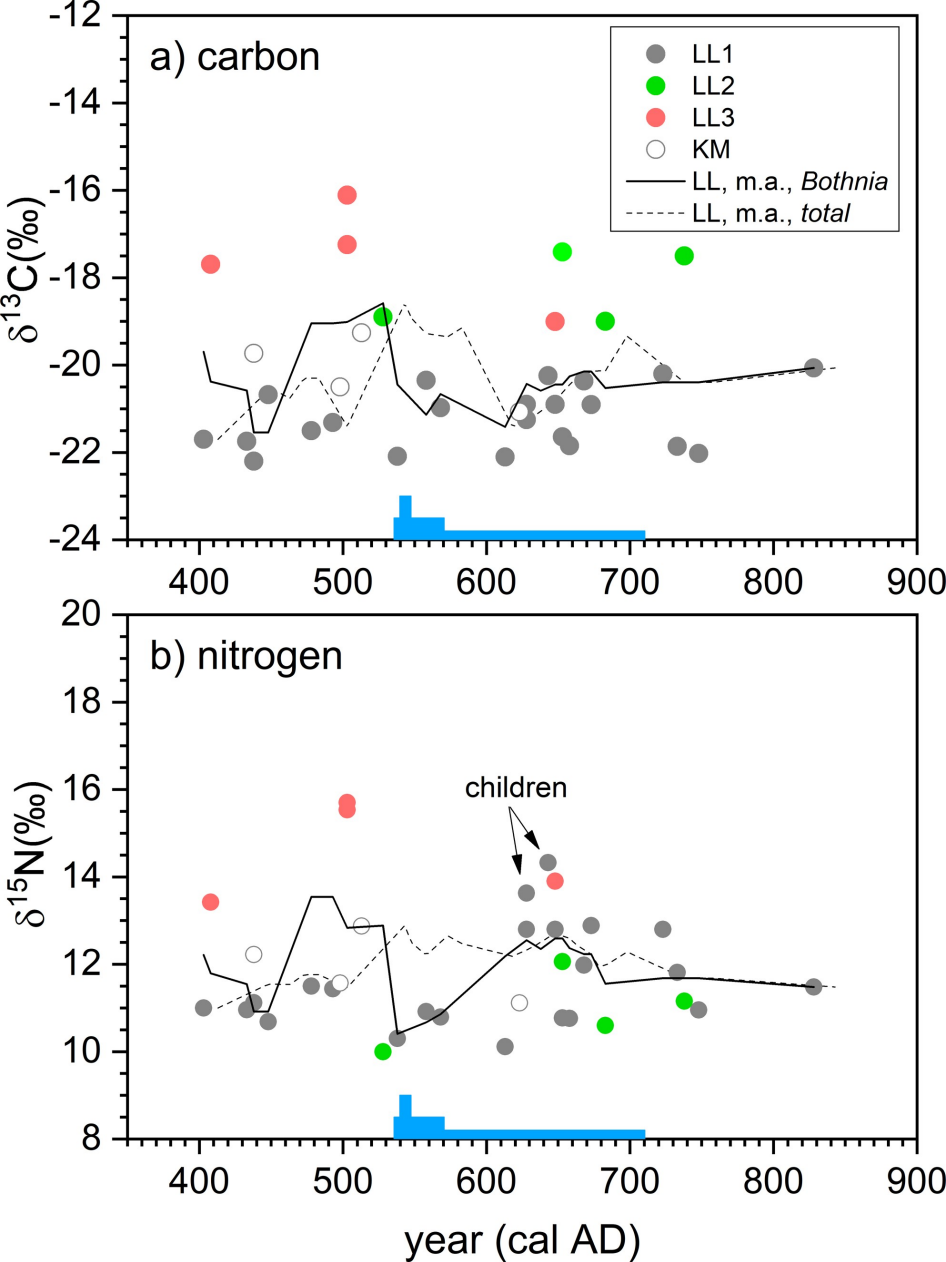
Time dependence of the subgroup-specific isotopic data. The time estimates have been obtained as mean values of the individual calendar-year probability distributions based on *Bothnia* (63–66°N) scenario. The two highest δ^15^N values of LL1 are of children and are indicated by arrows. The solid line is a moving average (m.a.) with a 50-year time window. As a sensitivity analysis, values (dashed lines) are given by also assuming the majority of the marine carbon originated from the whole Baltic basin (56–66°N, *total*). Chronologically, the round symbols correspond to the mean values of the calendar-year probability distributions of individual RE-corrected radiocarbon dates, taking into account 18 ± 5 yr bone own age[48]. The blue bars schematically illustrate the influence of the Late Antique Little Ice Age (LALIA) triggered by the volcanic anomaly of AD 536–550[6].

**Fig 6:**
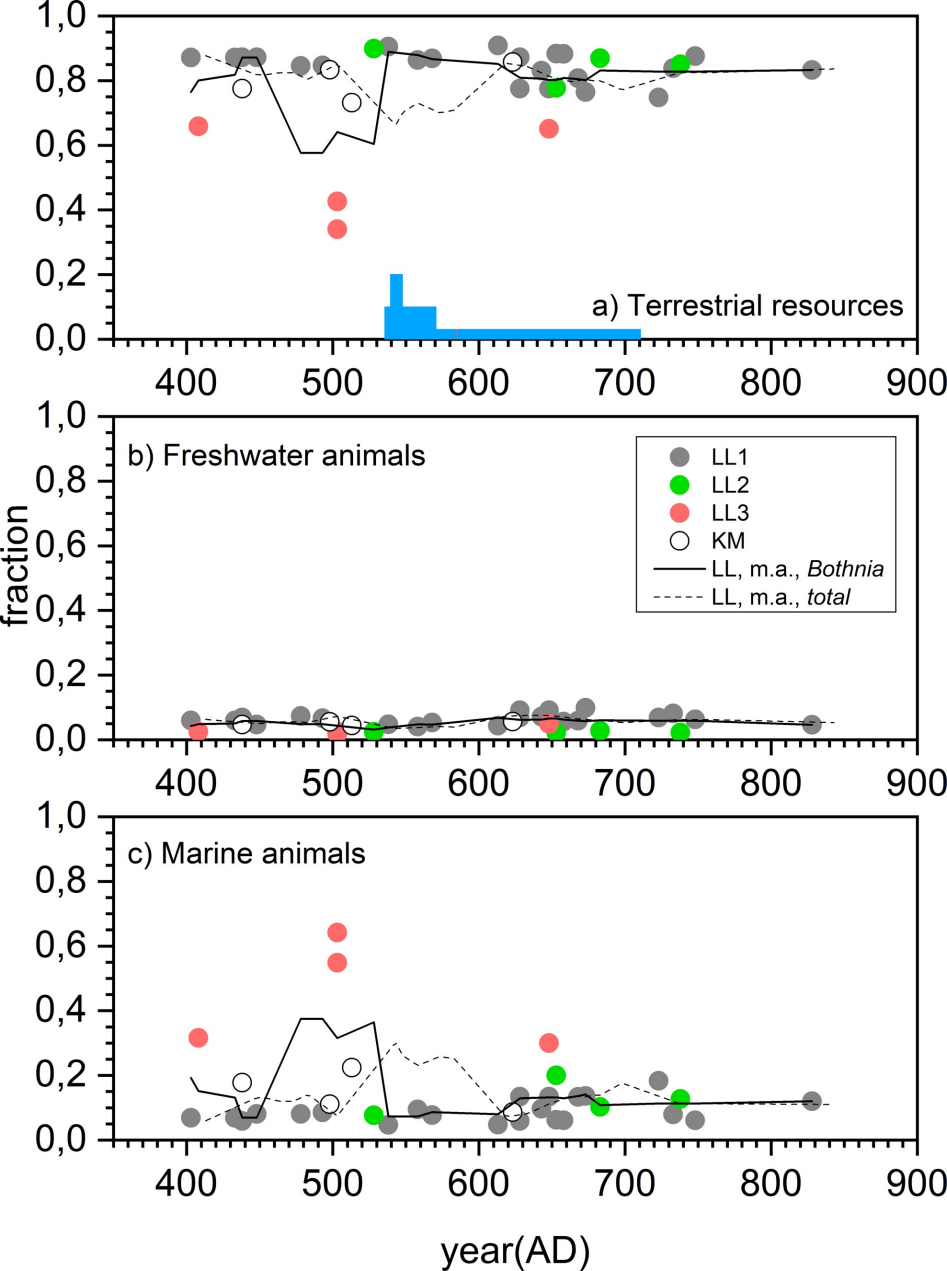
Time dependence of the food group fractions. The values have been obtained based on *Bothnia* (63–66°N) scenario. The solid line is a moving average (m.a.) with a 50-year time window. a) Terrestrial resources. b) Freshwater animals. c) Marine animals. As a sensitivity analysis, values (dashed lines) are given by also assuming the majority of the marine carbon originated from the whole Baltic basin (56–66°N, *total*). Chronologically, the round symbols correspond to the mean values of the calendar-year probability distributions of individual RE-corrected radiocarbon dates, taking into account 18 ± 5 yr bone own age[48]. The blue bars schematically illustrate the influence of the Late Antique Little Ice Age (LALIA) triggered by the volcanic anomaly of AD 536–550[6].

**Fig 7:**
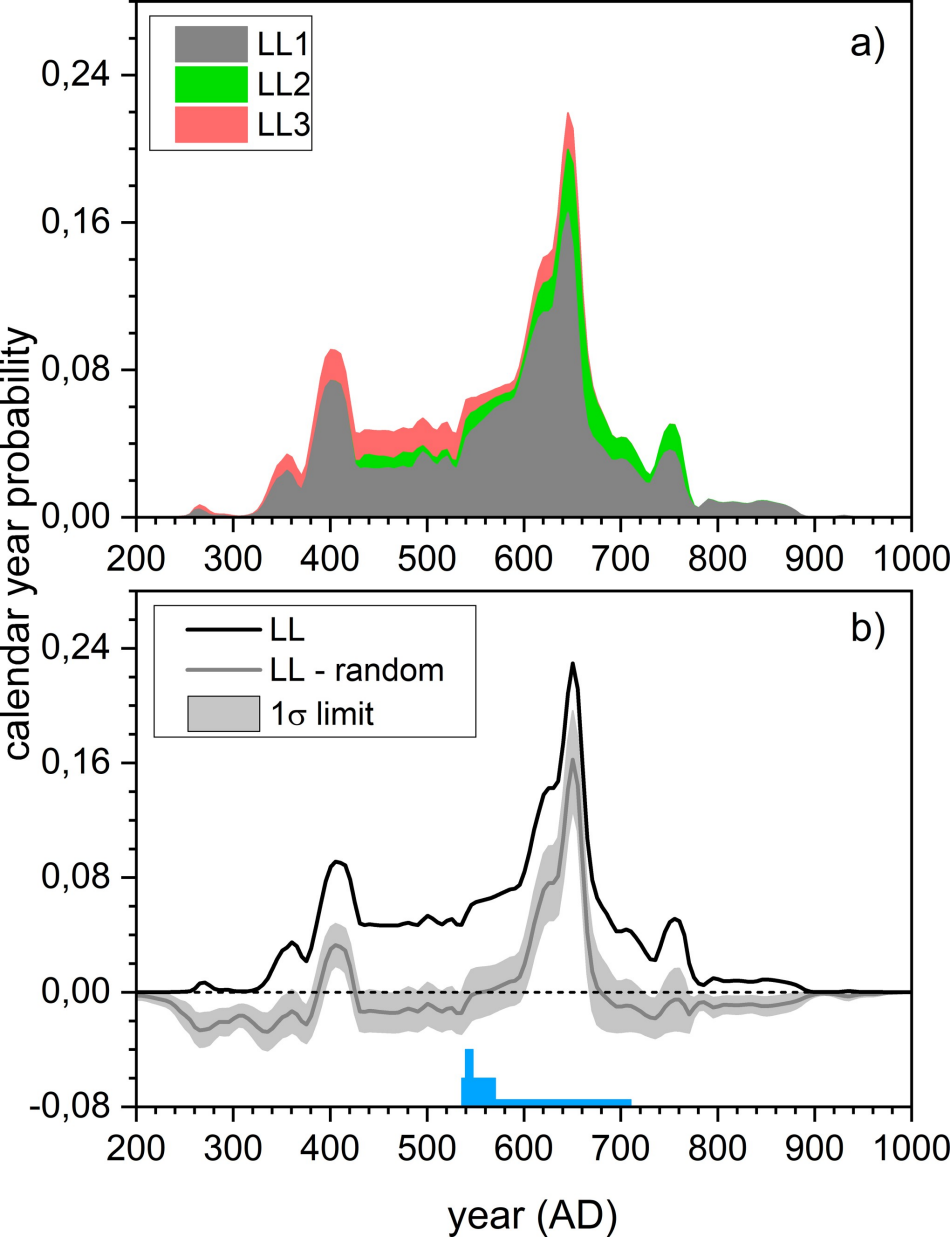
Summed calendar-year probability distributions. a) Divided according to subgroup LL1-3 and b) subtracted by the *scpd*s of randomly distributed ^14^C dates. The distributions indicate that a) the LL1 subgroup is responsible for most of the burial activity and b) the most intense period for the burial activity is AD 600–660, standing out from overall randomly distributed average. The blue bars schematically illustrate the influence of the Late Antique Little Ice Age (LALIA)[6], triggered by the climatic anomaly of AD 536–550.

There is no statistically significant increase in the δ^15^N data after the downturn (AD 536–570) (Fig 5, Table F in S4 Appendix). Probably the climatic downturn did not induce malnutrition long enough to be seen within the long bones with a slow turnover rate. In addition, there is no significant post-anomalous decrease in the terrestrial diet either (Fig 6A, Table L-M in S6 Appendix) that could potentially be attributed to crop losses. Low-TR individuals had already appeared before the anomaly and were characterized by large consumption of marine food. Furthermore, one does not see an immediate change in post-anomalous burial intensity (Fig 7B, Fig Na in S8 Appendix). Instead, the most significant *scpd* increase peaks a century later—too late to be interpreted as an increased mortality due to harvest failures caused by the anomaly. During the peak, two individuals with elevated δ^15^N values (Fig 5) can be explained (Text in S4 Appendix) as children still bearing breastfeeding influence[51,75]. The remaining LL1 individuals within the peak do not have statistically significant increase in their δ^15^N values with respect to the pre-anomalous era (Table F in S4 Appendix). So, regardless of the multitude of high-latitude indications of cultivation in the area[57–59,76,77], it seemed to remain isotopically a fairly invisible livelihood. This is consistent with the recent observation of only sporadic signs of barley and rye pollen from around AD 690 and continuous cultivation only from around AD 1350 onward close to Levänluhta[22]. In addition, it agrees with the proposed significant use of animal protein within diets. It seems that the base population continued to supplement their animal husbandry with hunting and fishing under the pressure of the decadal climatic downturn, and they were likely to have originally relied less on cultivation.

An interesting feature coinciding the climatic anomaly and the subsequent LALIA is the decreased role of LL2 and LL3 subgroups during the strongest LALIA (Figs 5 and 6, Text in S8 Appendix). As the mean values of *cpd*s of LL2 and LL3 do not fall within AD 540–650 the scattering of the isotopic ratios decrease drastically (Fig Nb in S8 Appendix) during AD 540–600 i.e. essentially during the strongest LALIA. This reflects the continuation of burials of only LL1 individuals during the period and, particularly, disappearance of burials of strongly marine LL3 individuals. However, as the individual *cpd*s are typically centennial-wide, the point-estimates of means may provide an incomplete view. Nevertheless, this feature is worth to consider further.

Seal hunting essentially relies on the extent of the ice margin[63]. For example, the cooling of the climate from AD 1570 to AD 1610 decreased the Ostrobothnian seal prey drastically since the edge of the firm ice moved southward, away from the strait of Kvarken[63,78]. The pre-anomalous climatic conditions in Ostrobothnia were essentially the same in the 16^th^ century AD as in the 6^th^ (Text in S1 Appendix). Therefore, analogous to the 16^th^-century cold period and shortage of seal prey, the ice edge receded southward due to decades of LALIA coldness, which probably resulted in poorer opportunities for seal hunting near the Kvarken. This would be consistent with the disappearance of strongly marine (LL3) individuals from the Levänluhta material and the subsequently reduced scatter within the isotopic values when sources of subsistence were reduced. However, within the LL1 base population, non-negligible marine fraction still remained. This emphasizes the role of the marine resources as a minor dietary supplement even through the climatic downturn. After the LALIA, the climate partly recovered and the ice edge inevitably returned northward, closer to the old sealing grounds, and probably increasing again the diversity of livelihoods and thus scattering. Throughout the Levänluhta era, the land uplift gradually evolved and, altogether, the shoreline receded away by about 10–15 km during four centuries (S3 Appendix). The post-glacial rebound created additional pressure for the Levänluhta people to reduce opportunities for marine subsistence. This may be reflected in the general trend of decreasing marine influence over the centuries.

## Discussion

### Resilience founded on versatile livelihoods

The standard deviations of their isotopic values (Fig Nb in S8 Appendix) quantify the observed livelihood heterogeneity—the spread of isotopic values is largest during the pre-anomalous period suggesting various and temporally overlapping livelihoods. This versatility is visible also within individual subgroups–nearly all subpopulations seemed to utilize all the available dietary resources, except LL2. The temporal overlap is further proven by *scpd*s of the three subgroups LL1-LL3 since they all possessed a certain amount of calendar-year probability (Fig 7) during the Migration period, indicating their contemporaneity with the pre-anomalous period. The diversity of livelihoods and flexibility to take advantage of changing conditions are the essence of resilience[79] that supported, for instance, persistence of the Canadian indigenous populations[80] and the Iron Age cultures of northern Sweden[81]. Sources of resilience were probably similar among the Levänluhta people: living on the boundaries of marine, freshwater, and terrestrial ecosystems enabled the diverse livelihoods that allowed them to adapt their dietary routines under the climatic downturn carrying them over the Fimbulvinter. Such resilience was likely sustained in Ostrobothnia as diverse livelihoods have been also suggested for the Bronze Age[17] and by the isotopic evidence of human remains for the historical Little Ice Age[82].

Indigenous Sámi have demonstrated versatile ways of living during the Iron Age, likely comparable to the Levänluhta population. Notably, in Northern Sweden their livelihoods resembled those of their Germanic neighbors in the south, including cultivation, animal husbandry and seal hunting[81]. Such punctuated sedentism, utilizing both the marine shoreline and terrestrial inland resources, provided them a long-term strategy for survival and maintained their sources of subsistence[83]. Indeed, starting from around AD 600, archaeological stray finds appear increasingly within the Northern Ostrobothnian river islands[84], indicating stronger shore–inland connections through these natural passageways. For the Levänluhta population, such an economy would have been mediated by the Kyrö river, supported by ridgelines[17]. Nearly equal division between the marine and terrestrial resources of LL3 individuals and the significant marine component of LL2 and KM (Table 2) indicate utilization of both marine littoral and terrestrial inland resources. Similarly, the peaked number of burials around AD 600–660 could also be linked to such increased shore–inland connections, bringing in a moderate marine influence to the LL1 base population, regardless of the shoreline displacement.

### Evolving of versatile community

Significant changes occurred all around the Baltic Sea during the anomalous period of the 6^th^ century. At the western side of the Gulf of Bothnia, the large settlements of Gene and Högom were abandoned around AD 550–600[85], when climatic hardships and the collapse of Roman trading routes possibly intermingled in a disastrous way[19]. The cold event is so visible in palynological and archaeological data in Estonia that the moment has been seen as a chronological boundary for a whole new archaeological era[13]. Cultures along the Northern Ostrobothnian shores, a couple of hundred kilometers north from the Levänluhta site, that relied on sealing and fishing, also seemed to have vanished circa AD 600 indicating that the collapse of the long-distance trading networks of Rome reached the northern areas[18]. In a broader context, the Justinian Plague spread within Europe from AD 542 onward, killing one-third of its population[86]. While the world seemed to fall apart around Eastern Fennoscandia after AD 536, the Merovingian culture flourished from around AD 550 onward in the old historical southwestern provinces of Finland proper, Satakunta, and Tavastia[61]. The notable increase of burials at Levänluhta during the Merovingian period may not be an independent phenomenon. Low population density, still successful trade, versatile livelihoods—not largely dependent on cultivation—and rich natural resources beyond the Suomenselkä ridgeline may have been attractive. Thus, instead of increased natality and/or mortality of the local population, another explanation for the AD 600–660 increase could be an inflow of new people practicing livelihoods similar to the base population.

Wide-ranging contacts with neighboring regions are seen throughout the Ostrobothnian Iron Age[61]—artifacts of Roman, Scandinavian, Baltic, and Eastern and Western European characteristics have been found[19]. Thus, the Ostrobothnian Iron Age culture was an integral part of the Northern European-wide trading network. The weak links to freshwater sources and the mixed diet of terrestrial and marine food of the LL2 individuals—resembling that of Viking Age Gotlanders—are coherent with a scenario in which these people assimilated their bone collagen isotopic signals within the Baltic proper instead of Ostrobothnia. Burials dated mainly to AD 550–700 are coherent with the contemporaneous archaeological finds of Levänluhta that indicate connections all around the Baltic Sea and especially to the Vendel period Scandinavia[19,22]. Indeed, the LL2 individuals might have been involved in forming contacts between these regions.

Two studies have recently shed light on the genetic and geographical background of the Levänluhta people[35,87]. The genome of the majority (4/5 i.e. 80%) of the studied Levänluhta individuals resembles that of the modern Sámi and one of modern Scandinavians/Lithuanians (Table P in S9 Appendix). This majority corresponds to the LL1 base population in magnitude (22/30 i.e. 73%; Table 2), suggesting a potential linkage between these populations. The existing Sr isotopic data on four individuals show also spread, indicating diverse geographical origins, including local, eastern Fennoscandian, or Scandinavian backgrounds[87]. A recent study of the oldest place names in the region does not support Iron Age toponyms of Finnish, Scandinavian, or Baltic origin[88]. Instead, several Sámi-based place names are recognized and the oldest river names—such as Kyrö—may point toward language reconstruction of Proto-Finno–Permian although contribution of Paleo-European languages cannot be excluded[88]. Thus, archaeologically visible versatility is reflected by genetics and linguistics, and this is consistent with the observed broad and fragmented isotopic distributions. Consequently, this establishes a testable hypothesis of a multiethnic and multiorigin community to be assessed in the future by studying life histories of individuals with additional ancient-DNA and oxygen and strontium isotope analyses.

### What is the Levänluhta water burial?

The Levänluhta water burial was first interpreted as a place for human sacrifice[89] and this interpretation gathered support throughout the 20^th^ century[90,91]. The human sacrifice would then be frequent (one every fourth year), spanning through several centuries and involving a heterogeneous community in terms of diet and cultural features. Such a massive and long-term sacrificial tradition would be truly unique worldwide. Further, the observed long period of usage excludes the possibility of the deceased being solely a result of an abrupt event of famine, disease, or battle. The peripherally located bog or lake with a hidden nature has motivated a new theory of the individuals being socially, ideologically, or ethnically deviant people within their society[22]. This is possible since socially or ideologically distinct communities might have practiced versatile livelihoods and thus resulted in broad isotopic distributions.

The site being a burial for ordinary people, or slaves, was discussed in the 1950s[16]. Yet, valuable artifacts related to the elite (Text in S2 Appendix) do not support the slave hypothesis. The number of grave goods (22 pcs), however, mismatches the number of buried individuals (98 pcs). The low number of grave goods can be explained in several ways. Not all deceased (probably children) were buried with grave goods, the goods were made of organic material that could have disintegrated in water, and early archaeological excavations may not have been able to sieve all the soil material excavated. The lack of significant portion of artifacts may also be connected to an archaeologically invisible period of AD 250–800 in Northern Finland, linked to fur trade and the ethnogenesis of the modern Sámi[92].

A concept of *sáiva* is linked to sacred places in the Sámi mythology, one manifestation being a spring-containing freshwater lake[93,94]. These lakes have been considered as a realm of human and animal spirits. The Levänluhta site was likely originally in a spring-containing lake[19]. In the Levänluhta and Käldamäki sites, the essential elements of *sáiva* are present: they are spring-containing freshwater underworlds of people with mainly Sámi-related genetic heritage (Levänluhta) and of people and domesticated animals (Käldamäki). Therefore, the *sáiva* tradition may have its roots in beliefs similar to those we witness in the Levänluhta and Käldamäki burials. If so, the past appearance of the site as a spring-containing lake could even explain why the Levänluhta location was chosen to bury the deceased. Over the centuries, this early manifestation has been hidden by the post-glacial land uplift that have gradually turned it into a bog, and later people have converted it to a field. Presently, three springs with upwelling reddish water carry remembrance of the past burial site.

### Conclusions

A chronological, dietary and livelihood synthesis is established based on radiocarbon and stable isotopic studies of human bone remains on the Iron Age population excavated from the unique spring burial of Levänluhta. Extraordinarily broad isotopic distribution is observed with standard deviations of multiple times compared to the reference populations within the Baltic Sea. Clustering analysis reveals three subgroups, most of them relying essentially on terrestrial, strongly animal, resources with significant contribution of marine and lesser freshwater food. This emphasizes the versatile livelihoods practiced at the boundary of marine, freshwater, and terrestrial ecosystems. Although the strongest marine signal disappears around the climatic downturn of the 6^th^ century AD, this versatility provided resilience to overcome the scourges of nature during and after the Fimbulvinter. Further, the study establishes testable hypotheses to assess potential role of multiethnicity within the broad isotopic distribution of the studied human remains. Eventually, the Levänluhta site is displayed as being a water burial of a versatile and resilient community of a population probably in the midst of an ethnogenesis toward the modern Sámi.

## Supporting information

S1 Appendix Climatic archives and effects. (PDF)

S2 Appendix Archaeological contexts of Levänluhta and Käldamäki. (PDF)

S3 Appendix Levänluhta and post-glacial land uplift. (PDF)

S4 Appendix Bone material and isotopic data. (PDF)

S5 Appendix Isotopic baseline. (PDF)

S6 Appendix Dietary modelling. (PDF)

S7 Appendix Timeline through radiocarbon dates. (PDF)

S8 Appendix Time series data. (PDF)

S9 Appendix Multidisciplinary evidence. (PDF)
