# Supplementary material for: Buried in water, burdened by nature—Resilience carried the Iron Age people through Fimbulvinter
Source: PLoS One. 2020 Apr 21;15(4):e0231787. doi: 10.1371/journal.pone.0231787 (PMC7173937; doi:10.1371/journal.pone.0231787)
Supplement: S1 Appendix — (PDF) [file pone.0231787.s001.pdf]

Supporting Information **S1 Appendix. Climatic archives and effects** for

Buried in water, burdened by nature – Resilience carried the Iron Age people through Fimbulvinter

Corresponding author: Markku Oinonen

Contributors: Samuli Helama, Heli Huhtamaa, Markku Oinonen

S1 Appendix contains: Text, Figure A-D

Text

Palaeoclimatic records

Probable climatic effects on agriculture over prehistoric times can, to large extent, be examined using proxy data. Collecting such data from regions where a single factor constitutes the climatic signal in that record helps illustrating and even reconstructing climatic variability beyond the era of instrumental measurements[1]. Importantly, tree-ring data provide high-resolution insights into past climate variability without dating uncertainties[2,3]. Moreover, different types of dendroclimatic records (i.e. climate-sensitive tree-ring width, density and isotope chronologies) may be compared with instrumental or modern observational data and the information gained from such analyses applied back in time to infer past climate and climate-related variations[4,5]. These benefits notwithstanding, the use of tree-ring data over the first millennium AD is generally limited due to low number of correspondingly long chronologies[6,7]. Fortunately, there are different types of Fennoscandian tree-ring chronologies spanning the first millennium AD which may be used to set the archaeological events observed at the Levänluhta site in context of climatic information. Topically, the value of these chronologies to indicate past climate-related agricultural calamities has previously been successfully demonstrated[8–12].

First, multitude of tree-ring width studies have demonstrated the negative consequences of the volcanic forcing, particularly of AD 536 – 550 as tree-growth decline throughout the Northern Hemisphere[15–21]. **Second**, there has been a long-lasting interest in the production of the maximum latewood density (MXD) records from the northern parts of the region (for literature review, see Matskovsky and Helama 2014[13]) and the composite MXD chronologies based both on subfossil and living tree (*Pinus sylvestris* L.) materials now cover the past two millennia[21,22]. This dendroclimatic data is highly sensitive to variations in summer (June through August; JJA) temperature and the MXD data has been used to reconstruct annual-to-millennial JJA temperature variability in the same region[13,17,21–25]. Here we use the MXD-based reconstruction[13] (Fig A) that benefits from large data collection, combination of tree-ring analysis methods including the regional curve standardization (RCS)[24,26] and its correction implementations[27,28]. It is available online as the NOAA palaeoclimate data contribution at https://www.ncdc.noaa.gov/paleo-search/study/17264. This MXD proxy

explains more than 60% of the observed JJA temperature variability[13]. **Third**, the studies of the AD 536 - 550 climatic anomaly have extended to isotopic characterizations[29] and the recent development to construct tree-ring stable isotope chronologies for the Fennoscandia[30–32] has now resulted in annually resolved data of $^{13}C/^{12}C$ ratio ($\delta^{13}C$) that overlaps the mid-sixth century AD anomalies i.e. the period most topical to the underlying research questions of this study. Sensitivity of Arctic tree growth towards amount of sunlight has been well demonstrated and, particularly, photosynthetically active radiation (PAR) is considered as possibly the most important forcing factor for stable carbon isotopic ratios ($\delta^{13}C$) of northern conifers[33–36]. Compared over the instrumental period, our recent $\delta^{13}C$ data show strong climatic signals related to incoming solar radiation[36,37], agreeing with the previous findings from adjacent areas[35].

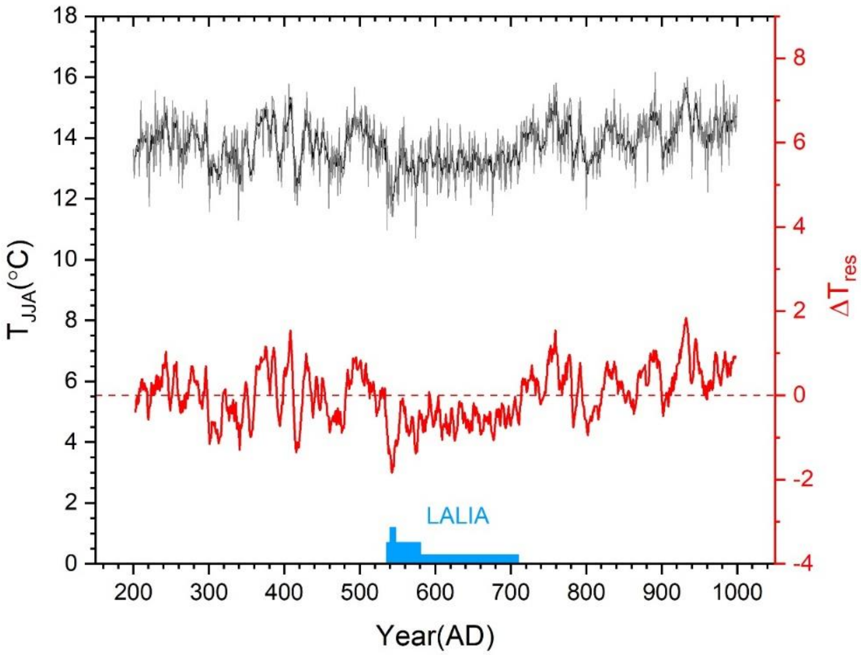

Fig A. Temperature reconstruction based on maximum latewood density (MXD) data[13] from northern Finland. Up: Annual MXD signal (grey) and its 5yr average (black). Down: Residual defined as $\Delta T_{JJA} = T_{JJA} - T_{JJA,\ ave}$. Long negative temperature anomaly spanning from AD 536 to ca. AD 710 resembles that of the Late Antique Little Ice Age (LALIA) proposed by Büntgen et al[14]. The length and the influence of this cold period are shown schematically with blue bars. This schematic representation has been used throughout the paper.

As a consequence, the $\delta^{13}C$ data were recently used to reconstruct past variations in June-July irradiance between AD 519 and 610[37] (Fig B). Particularly, during AD 541-543 the light irradiance reduced from ~190 W/m^2 to ~135 W/m^2 corresponding to nearly 30% loss. Also this reconstruction is based on tree-ring $\delta^{13}C$ data produced using the RCS methods and explains more than 50% of the observed irradiance variability. Although the variations in summertime temperature and irradiance may be found interrelated[38], it

was shown that the respective climatic signals are the most prominent drivers of the MXD and $\delta^{13}C$ data in this region. That is, the MXD and $\delta^{13}C$ data did not correlate significantly with instrumentally observed irradiance and temperature (JJA) records when these data were made statistically independent to each other. Instead, the MXD and $\delta^{13}C$ proxies could be correlated significantly only with temperature and irradiance, respectively (see Figs S7 and S8 in Helama et al. 2018[37]). The irradiance reconstruction we use in this study is available online as the supplementary material of Helama et al. (2018)[37] downloadable at https://www.nature.com/articles/s41598-018-19760-w and the underlying isotope data as the NOAA contribution at https://www.ncdc.noaa.gov/paleo/study/23374.

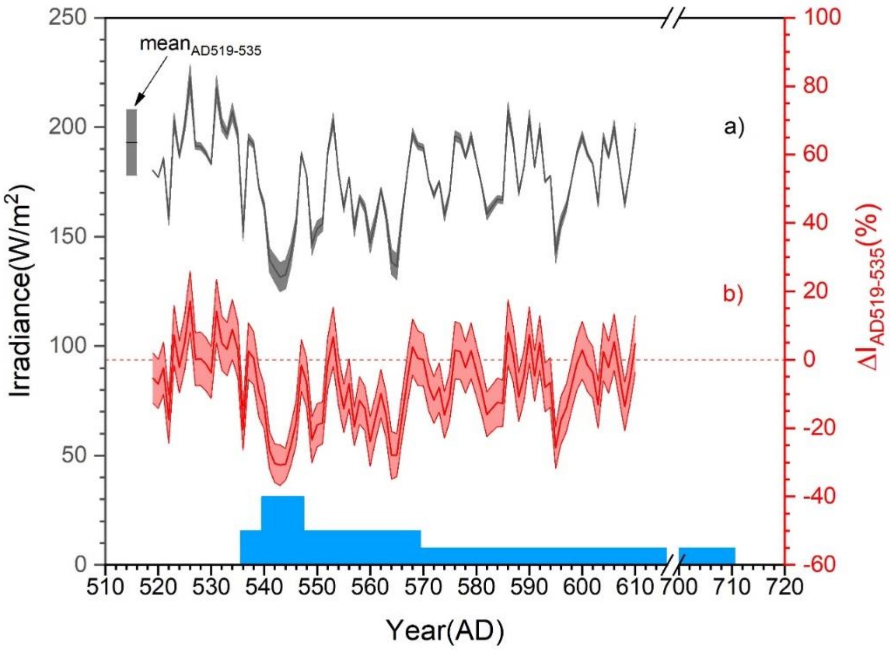

Fig B. Reconstructed solar irradiance based on annual $\delta^{13}C$ measurements on pine (*Pinus sylvestris*) from northern Finland[37]. a) Solar irradiance (black line) and its estimated uncertaint (grey), and the mean irradiance of AD 519 - 535. b) Difference (red line) between the reconstructed solar irradiance and the mean irradiance of AD 519 - 535. The uncertainties (light red) have been estimated through Monte-Carlo simulation based on a).

Consequences of climatic downturns

According to Monteith[39] the efficiency of converting solar energy to photosynthetic dry mass can be estimated as a product of multiple factors, including atmospheric transmission of sunlight. Consequently, production of dry mass by photosynthesis, i.e. primary production, is inversely proportional to the absorption of sunlight by the atmospheric particles (clouds, dust, aerosols) and directly proportional to the amount of sunlight eventually available for photosynthesis (photosynthetically active radiation

PAR). Recently, loss of sunlight induced by the mid-6th century AD volcanic winter was quantified through stable carbon isotopic ratio measurements of Arctic Scotch pine[37]. The anomalic dark and cold period lasted nearly a decade and triggered a longer cold period of the Late Antique Little Ice Age (LALIA) the strongest influence lasting until ca. AD 570[6] and milder until AD 710 (Fig A). In fact, most of the abrupt negative wood growth anomalies during the last 2000 years have been due to volcanic winters[40].

Both the MXD and $\delta^{13}C$ proxies are closely related to agricultural potential in the study region. Comparing the MXD data[13,41] with rye (*Secale cereale*) and barley (*Hordeum vulgare*) yields[10], their similar positive (negative) responses to warm (cold) growing season temperatures were demonstrated over a period of non-industrialised cultivation over most of Finland (AD 1861-1913), and particularly for southern Ostrobothnia. Significantly, low temperature and significant crop loss of AD 1868 was accompanied with a low MXD signal[10] and the climatic decline led to a well-known famine and death of ca. 150 000 people in Finland. Moreover, this strong relationship enabled an MXD-based reconstruction of climate-mediated yield ratio (pertaining to harvested grain in relation to sown) in central and northern Finland over the late Holocene period[11]. Unfortunately, this reconstruction does not overlap the entire first millennium AD but was built over the timeline from AD 760 to 2000, this being due to reduced number of MXD series from middle/south boreal tree-ring archives[41] especially over the pre-AD 1000 period. More recently, however, this reconstruction was explored for climatic, agricultural and societal responses resulting from abrupt negative temperature departures in western Finland over the 17th century AD following the explosive volcanic eruptions[12]. Additional indications of agricultural success over the earlier times can be derived from tree-ring isotopes. Given that the $\delta^{13}C$ data is strongly indicate of irradiance[35–37] that controls the terrestrial photosynthetic production and crop yields[42], the $\delta^{13}C$ variations must be directly related to potential of the contemporaries to utilise the agricultural plant products.. Collectively, the analyses performed demonstrate the value of MXD proxies to quantify the variations in climate-mediated yield ratio over the historical period and the potential of using the long MXD records to infer the first millennium yield ratios on annual resolution. Moreover, the negative $\delta^{13}C$/irradiance anomalies are explained to mean limited supply of harvested grain. These results reinforce the view of historical cultivation being highly sensitive to climate anomalies.

Alternative method to assess the possible agricultural impacts of the 6th century eruptions is to compare the impacts of latter volcanic eruptions on yields. However, it may not be reasonable to explore relationships between eruptions and crop yields with data from the era of industrial agriculture if aiming to explore the impacts on pre-industrial or prehistorical era, as agricultural practices have changed considerable during the recent past[8,43]. Prior the era of modern crop yield statistics and industrial agriculture, grain tithes – approximately one-tenth of the yield paid as a tax – are commonly used as proxy data to explore pre-industrial harvest fluctuations[44–46]. Yet, the practices to collect the grain tithes varied over different regions and times. For the Southern Ostrobothnia, the grain tithes were based on the annual rye and barley harvest output over the seventeenth

century[12]. Three large volcanic eruptions, Huaynaputina in AD 1600, Parker in AD 1641 and the unknown eruption in AD 1695, took place over this century[40].

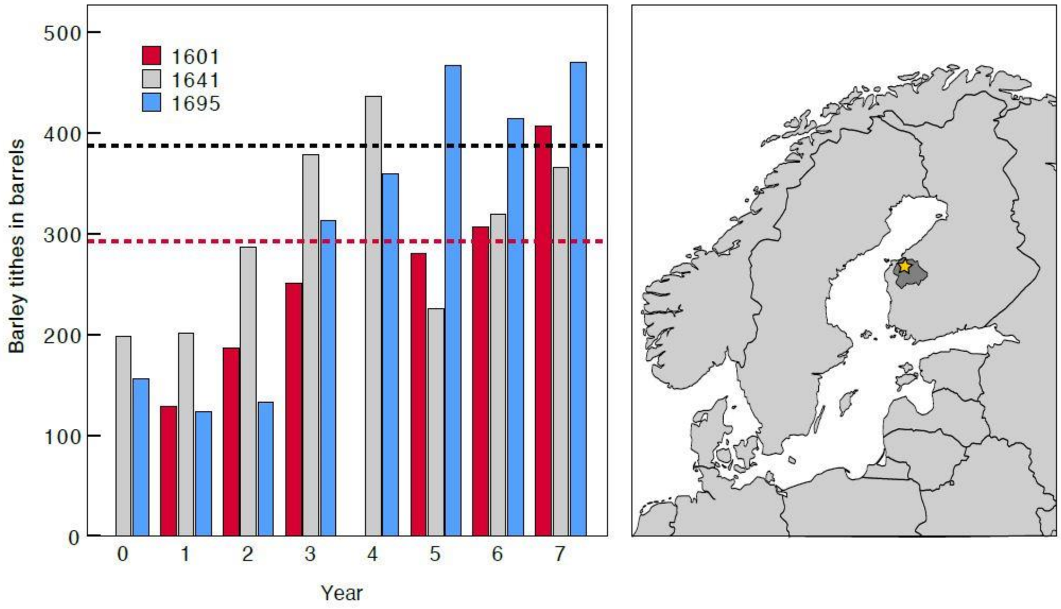

Fig C. Collected barley tithes (in barrels, c. 146.5 litres) on the year of peak volcanic forcing (year zero) and seven succeeding years[11]. The dashed lines (red for AD 1601 and black for AD 1641 and AD 1695) indicate the mean barley tithes on three data years preceding the volcanic events. In year AD 1601 tithes were not collected at all due to total harvest failure. The location of the Levänluhta has been marked with a star. Map was made with Natural Earth data (https://www.naturalearthdata.com/) and created by using QGIS 3.4. (https://www.qgis.org/).

The tithe data suggest that the harvest was half of the century mean on the year of the peak global volcanic forcing (AD 1601, AD 1641 and AD 1695, respectively[12]). Moreover, following the year of first crop failure, the harvest remained notably below the mean over one or two subsequent years. The agricultural impact of the 17th century eruptions appears to be especially profound with barley (Fig C). These harvest failures had, in turn, considerable impact on the wealth and wellbeing of the 17th century agricultural population, as their subsistence was largely dependent on harvest success. Administrative written records indicate that sudden impoverishment followed each volcanic event[12]. Furthermore, the human resilience to cope with adverse climate and resulting harvest failures was low in the 17th century due to man-made factors – such as political instability, population growth, increased social inequality and poor trade networks[12,47,48]. Consequently, the crises that were triggered by the AD 1601 and AD 1695 anomalies accelerated to catastrophic famines and demographic crisis (Fig D). Overall, the comparative approach suggests that these 17th century eruptions, which were likely lesser volcanic events than the AD 536 and AD 540 eruptions, and related anomalies in temperatures and irradiance caused sharp decline in grain yields and considerable socio-economic consequences in the Southern Ostrobothnia[12].

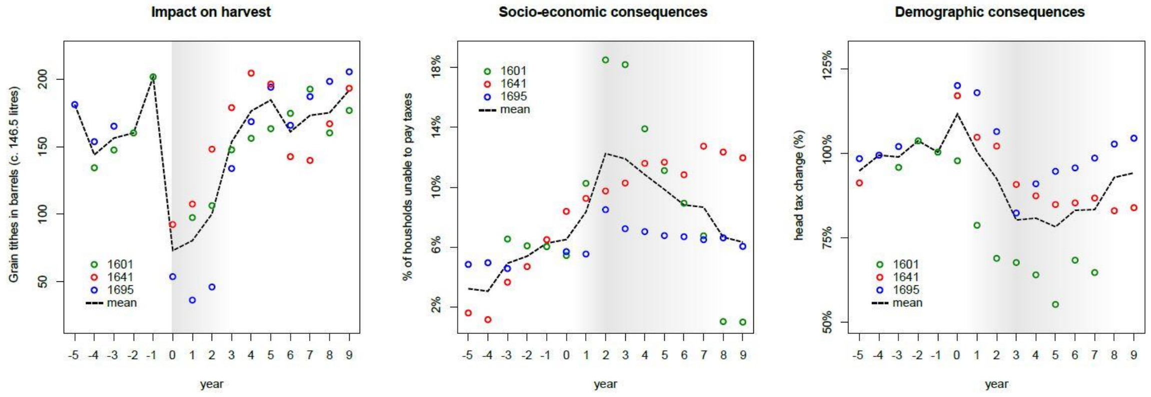

Fig D. Impact of the 17th century volcanic eruptions on harvest, their socio-economic and demographic consequences. The data is based on Huhtamaa and Helama (2017)[12].

Importance of seal hunting at ca. AD 300 is demonstrated by the ancient Roman price list Edict of Diocletian[49–51]. Seal skin was, together with leopard skin, the most valued skin quality and its value corresponded to 25 times the daily income of an artisan. Moreover, seal oil has been traditionally used within Europe in oil lamps for lighting. Similarly than cultivation, seal hunting within the Baltic Sea[50] have also faced climatic consequences[52]. During the 16th and 17th century AD seal hunting was practiced – particularly in Ostrobothnia – by seal hunters and local peasants through long-distance expeditions lasting several months. Historical records reveal the large preys obtained: during the peak year in AD 1558 the exported amount of seal oil from Finland was 307 000 litres[50]. At the end of the 16th century AD the climate cooled when the Little Ice Age started to set in. The coldness extended the annual ice cover of the Gulf of Bothnia and the breeding grounds of grey seal (*Halichoerus grypus*) moved southwards. Consequently, the seal preys of Ostrobothnia declined drastically after ca. AD 1570[50,52]. It is reasonable to think that the most significant climatic disturbance during the last 2000 years would have caused even larger effect.

Written and archaeological evidence suggests that the human consequences of the AD 536–550 event – extended to the Late Antique Little Ice Age (LALIA)[14] - were diverse and widespread, stretching from the British Isles to the Far East and from North Africa to Scandinavia[53]. Contemporary written records from the Mediterranean region document a mystery cloud dimming the sun in AD 536 for more than a year. The same sources record persistent cold and drought and one to two years of bad harvests being associated with the anomaly[54]. Sources from British Isles likewise mention crop failure and dearth in AD 536–539[15]. Furthermore, Chinese sources notion signs of dimming of the sky, unusual cold events and crop failure in AD 536–537[53]. In Northern Europe, particularly in Scandinavia and Estonia, agriculture suffered and settlements were abandoned after the cold years of AD 540s[55,56]. In many locations of these regions, the harvest shortfalls caused food shortage – even famine – during the following years. Moreover, the outbreak of the Justinian plague in AD 542 contributed to the hardship following the climatic deterioration and crop failures in Europe at the turn of the AD

530/540s[53,57]. The spread of the pandemic may have been accelerated due to the impacts of decreasing temperatures and irradiance on food availability and human immune system[14,37,58,59].

There are well-documented negative climatic impacts on cultivation and seal hunting during the 16th and 17th centuries AD within Ostrobothnia induced by volcanic eruptions and by the onset of the Little Ice Age. As the period of AD 536-550 has been the coldest decade observed during the last two millennia[40] and was extended to longer climatic disturbance of LALIA[14], it is reasonable to assume that this climatic downturn had similar, if even worse, negative influence in Ostrobothnia, particularly through losses of cultivated crops and receding seal breeding grounds. This assumption is quantitatively supported by nearly similar averages of reconstructed temperatures for pre-anomalic periods AD 500-535 ($T_{JJA,\ ave\ 500\text{-}535}$ = 13.8(9) °C) and AD 1530-1570 ($T_{JJA,\ ave\ 1530\text{-}1570}$ = 13.8(8) °C) based on Matskovsky and Helama (2014)[13]. The decadal light intensity loss inevitably reduced the primary production accordingly and eventually this would have led to severe consequences for societies dependent on cultivation – particularly at the northern latitudes where plant growth correlates strongly with light intensity and where long volcanic winter has multitude of chances to reduce the summer-night temperatures even below zero inducing frost damage on crops.

References

1. Bradley RS. Paleoclimatology: Reconstructing Climates of the Quaternary. Academic Press; 1999.
2. Fritts HC. Tree rings and climate. Academic Press; 1976.
3. Schweingruber FH. Tree Rings. Basics and Applications of Dendrochronology. Dordrecht: Springer Netherlands; 1988. doi:10.1007/978-94-009-1273-1
4. Briffa KR. Annual climate variability in the Holocene: interpreting the message of ancient trees. Quat Sci Rev. 2000;19: 87–105. doi:10.1016/S0277-3791(99)00056-6
5. McCarroll D, Loader NJ. Stable isotopes in tree rings. Quat Sci Rev. 2004;23: 771–801. doi:10.1016/J.QUASCIREV.2003.06.017
6. Helama S, Jones PD, Briffa KR. Limited Late Antique cooling. Nat Geosci. 2017;10: 242–243. doi:10.1038/ngeo2926
7. Riechelmann DFC, Gouw-Bouman MTIJ. A review of climate reconstructions from terrestrial climate archives covering the first millennium AD in northwestern Europe. Quat Res. 2018; 1–21. doi:10.1017/qua.2018.84
8. Holopainen J, Helama S. Little Ice Age Farming in Finland: Preindustrial Agriculture on the Edge of the Grim Reaper's Scythe. Hum Ecol. 2009;37: 213–225. doi:10.1007/s10745-009-9225-6
9. Helama S, Holopainen J, Macias-Fauria M, Timonen M, Mielikäinen K. A chronology of climatic downturns through the mid- and late-Holocene: tracing the distant effects of explosive eruptions from palaeoclimatic and historical evidence in northern Europe. Polar Res. 2013;32: 15866. doi:10.3402/polar.v32i0.15866
10. Huhtamaa H, Helama S, Holopainen J, Rethorn C, Rohr C. Crop yield responses to temperature fluctuations in 19th century Finland: provincial variation in relation to

climate and tree-rings. Boreal Environ Res. 2015;20: 707–723. Available: http://www.borenv.net/BER/pdfs/ber20/ber20-707.pdf
11. Huhtamaa H, Helama S. Reconstructing crop yield variability in Finland: Long-term perspective of the cultivation history on the agricultural periphery since ad 760. The Holocene. 2017;27: 3–11. doi:10.1177/0959683616646188
12. Huhtamaa H, Helama S. Distant impact: tropical volcanic eruptions and climate-driven agricultural crises in seventeenth-century Ostrobothnia, Finland. J Hist Geogr. 2017;57: 40–51. doi:10.1016/J.JHG.2017.05.011
13. Matskovsky VV, Helama S. Testing long-term summer temperature reconstruction based on maximum density chronologies obtained by reanalysis of tree-ring data sets from northernmost Sweden and Finland. Clim Past. 2014;10: 1473–1487. doi:10.5194/cp-10-1473-2014
14. Büntgen U, Myglan VS, Ljungqvist FC, McCormick M, Di Cosmo N, Sigl M, et al. Cooling and societal change during the Late Antique Little Ice Age from 536 to around 660 AD. Nat Geosci. 2016;9: 231–236. doi:10.1038/ngeo2652
15. Baillie MGL. Dendrochronology raises questions about the nature of the AD 536 dust-veil event. The Holocene. 1994;4: 212–217. doi:10.1177/095968369400400211
16. Salzer MW, Hughes MK. Bristlecone pine tree rings and volcanic eruptions over the last 5000 yr. Quat Res. 2007;67: 57–68. doi:10.1016/J.YQRES.2006.07.004
17. Grudd H. Torneträsk tree-ring width and density ad 500–2004: a test of climatic sensitivity and a new 1500-year reconstruction of north Fennoscandian summers. Clim Dyn. 2008;31: 843–857. doi:10.1007/s00382-007-0358-2
18. Buntgen U, Tegel W, Nicolussi K, McCormick M, Frank D, Trouet V, et al. 2500 Years of European Climate Variability and Human Susceptibility. Science (80-). 2011;331: 578–582. doi:10.1126/science.1197175
19. D'Arrigo R, Jacoby G, Frank D, Pederson N, Cook E, Buckley B, et al. 1738 years of Mongolian temperature variability inferred from a tree-ring width chronology of Siberian pine. Geophys Res Lett. 2001;28: 543–546. doi:10.1029/2000GL011845
20. Esper J, Düthorn E, Krusic PJ, Timonen M, Büntgen U. Northern European summer temperature variations over the Common Era from integrated tree-ring density records. J Quat Sci. 2014;29: 487–494. doi:10.1002/jqs.2726
21. Esper J, Frank DC, Timonen M, Zorita E, Wilson RJS, Luterbacher J, et al. Orbital forcing of tree-ring data. Nat Clim Chang. 2012;2: 862–866. doi:10.1038/nclimate1589
22. Melvin TM, Grudd H, Briffa KR. Potential bias in 'updating' tree-ring chronologies using regional curve standardisation: Re-processing 1500 years of Torneträsk density and ring-width data. The Holocene. 2013;23: 364–373. doi:10.1177/0959683612460791
23. Briffa KR, Bartholin TS, Eckstein D, Jones PD, Karlén W, Schweingruber FH, et al. A 1,400-year tree-ring record of summer temperatures in Fennoscandia. Nature. 1990;346: 434–439. doi:10.1038/346434a0
24. Briffa KR, Jones PD, Bartholin TS, Eckstein D, Schweingruber FH, Karlén W, et al. Fennoscandian summers from ad 500: temperature changes on short and long timescales. Clim Dyn. 1992;7: 111–119. doi:10.1007/BF00211153
25. Matskovsky V, Helama S. Direct transformation of tree-ring measurements into

palaeoclimate reconstructions in three-dimensional space. The Holocene. 2016;26: 439–449. doi:10.1177/0959683615609748
26. Helama S, Melvin TM, Briffa KR. Regional curve standardization: State of the art. The Holocene. 2017;27: 172–177. doi:10.1177/0959683616652709
27. Melvin TM, Briffa KR. A “signal-free” approach to dendroclimatic standardisation. Dendrochronologia. 2008;26: 71–86. doi:10.1016/J.DENDRO.2007.12.001
28. Matskovsky VV. Estimation of Biases in RCS Chronologies of Tree Rings. J Sib Fed Univ Biol. 2011;4: 389–404.
29. Churakova Sidorova O V., Bryukhanova M V., Saurer M, Boettger T, Naurzbaev MM, Myglan VS, et al. A cluster of stratospheric volcanic eruptions in the AD 530s recorded in Siberian tree rings. Glob Planet Change. 2014. doi:10.1016/j.gloplacha.2014.08.015
30. Helama S, Arppe L, Timonen M, Mielikäinen K, Oinonen M. Age-related trends in subfossil tree-ring $\delta^{13}C$ data. Chem Geol. 2015;416. doi:10.1016/j.chemgeo.2015.10.019
31. Helama S, Arppe L, Hyvönen J, Mielikäinen K, Oinonen M. Isoscapes of plant δ13C in the northern forests: Addressing the question of inter-tree and -site variability in Pinus sylvestris L. tree rings from Finnish Lapland. Geochem J. 2018;52: 287–298. doi:10.2343/geochemj.2.0507
32. Helama S, Arppe L, Timonen M, Mielikäinen K, Oinonen M. A 7.5 ka chronology of stable carbon isotopes from tree rings with implications for their use in palaeo-cloud reconstruction. Glob Planet Change. 2018;170: 20–33. doi:10.1016/J.GLOPLACHA.2018.08.002
33. McCarroll D, Jalkanen R, Hicks S, Tuovinen M, Gagen M, Pawellek F, et al. Multiproxy dendroclimatology: a pilot study in northern Finland. The Holocene. 2003;13: 829–838. doi:10.1191/0959683603hl668rp
34. Young GHF, McCarroll D, Loader NJ, Kirchhefer AJ. A 500-year record of summer near-ground solar radiation from tree-ring stable carbon isotopes. The Holocene. 2010;20: 315–324. doi:10.1177/0959683609351902
35. Loader NJ, Young GHF, Grudd H, McCarroll D. Stable carbon isotopes from Torneträsk, northern Sweden provide a millennial length reconstruction of summer sunshine and its relationship to Arctic circulation. Quat Sci Rev. 2013;62: 97–113. doi:10.1016/J.QUASCIREV.2012.11.014
36. Helama S, Arppe L, Uusitalo J, Mäkelä HM, Oinonen M, Mielikäinen K. Coexisting responses in tree-ring $\delta^{13}C$ to high-latitude climate variability under elevated CO<inf>2</inf>: A critical examination of climatic effects and systematic discrimination rate changes. Agric For Meteorol. 2016;226–227. doi:10.1016/j.agrformet.2016.06.005
37. Helama S, Arppe L, Uusitalo J, Holopainen J, Mäkelä HM, Mäkinen H, et al. Volcanic dust veils from sixth century tree-ring isotopes linked to reduced irradiance, primary production and human health. Sci Rep. 2018;8: 1339. doi:10.1038/D1598-018-19760-w
38. Helama S. Climate and Scots pine tree-rings in Utsjoki-Kevo district (North- East Finnish Lapland) during the 20th century, with special emphasis on mid-summer connections. Reports from Kevo Subarctic Research Station 24. 2011.

39. Monteith JL. Solar Radiation and Productivity in Tropical Ecosystems. J Appl Ecol. 1972;9: 747. doi:10.2307/2401901
40. Sigl M, Winstrup M, McConnell JR, Welten KC, Plunkett G, Ludlow F, et al. Timing and climate forcing of volcanic eruptions for the past 2,500 years. Nature. 2015;523: 543–549. doi:10.1038/nature14565
41. Helama S, Vartiainen M, Holopainen J, Mäkelä H, Kolström T, Meriläinen J. A palaeotemperature record for the Finnish Lakeland based on microdensitometric variations in tree rings. Geochronometria. 2014;41: 265–277. doi:10.2478/s13386-013-0163-0
42. Proctor J, Hsiang S, Burney J, Burke M, Schlenker W. Estimating global agricultural effects of geoengineering using volcanic eruptions. Nature. 2018;560: 480–483. doi:10.1038/D1586-018-0417-3
43. Hietala-Koivu R. Landscape and modernizing agriculture: a case study of three areas in Finland in 1954–1998. Agric Ecosyst Environ. 2002;91: 273–281. doi:10.1016/S0167-8809(01)00222-5
44. Kain R. Tithe as an Index of Pre-Industrial Agricultural Production. Agric Hist Rev. 1979;27: 73–81. doi:10.2307/40274034
45. Vamplew W. Tithes and Agriculture: Some Comments on Commutation. Econ Hist Rev. 1981;34: 115–119. doi:10.1111/j.1468-0289.1981.tb02009.x
46. Le Roy Ladurie E, Goy J. Tithe and agrarian history from the fourteenth to the nineteenth centuries : an essay in comparative history. Cambridge University Press; 2008. Available: https://www.cambridge.org/vi/academic/subjects/history/european-history-after-1450/tithe-and-agrarian-history-fourteenth-nineteenth-century-essay-comparative-history?format=PB
47. Jutikkala E. The great finnish famine in 1696–97. Scand Econ Hist Rev. 1955;3: 48–63. doi:10.1080/03585522.1955.10411468
48. Huhtamaa H. Combining Written and Tree-Ring Evidence to Trace Past Food Crises: A Case Study from Finland. Famines During the ‘Little Ice Age’ (1300-1800). Cham: Springer International Publishing; 2018. pp. 43–66. doi:10.1007/978-3-319-54337-6_3
49. Frank T. An economic survey of ancient Rome, Vol. V. New York; 1940.
50. Ylimaunu J. Itämeren hylkeenpyyntikulttuurit ja ihminen-hylje-suhde [Baltic seal hunting cultures and human-seal relationship]. Helsinki: Suomalaisen Kirjallisuuden Seura; 2000.
51. Kropff A. New English translation of the Price Edict of Diocletianus. 2016. Available: http://www.academia.edu/23644199/New_English_translation_of_the_Price_Edict_of_Diocletianus
52. Kvist R. Klimathistoriska aspekter på sälfångst i Österbotten 1551-1610. In: Sander A, Hallinder P, Jansson S, Viklund S-H, editors. Bottnisk kontakt IV: maritimhistoriska konferens, Skellefteå Museum. Skellefteå: Skellefteå Museum; 1989. pp. 86–95.
53. Newfield TP. The Climate Downturn of 536–50. The Palgrave Handbook of Climate History. London: Palgrave Macmillan UK; 2018. pp. 447–493. doi:10.1057/978-1-137-43020-5_32

54. Arjava A. The Mystery Cloud of 536 CE in the Mediterranean Sources. Dumbart Oaks Pap. 2005;59: 73–94.
55. Gräslund B, Price N. Twilight of the gods? The 'dust veil event' of AD 536 in critical perspective. Antiquity. 2012;86: 428–443. doi:10.1017/s0003598x00062852
56. Tvauri A. The Impact of the Climate Catastrophe of 536-537 AD in Estonia and Neighbouring Areas. Est J Archaeol. 2013;18: 30–56. Available: https://www.questia.com/library/journal/1G1-372251465/the-impact-of-the-climate-catastrophe-of-536-537-ad
57. Kostick, C., Ludlow F. The Dating of Volcanic Events and their Impacts upon European Climate and Society, 400–800 CE. Eur J Post-Classical Archaeol. 2015;5: 7–30.
58. McCormick M, Büntgen U, Cane MA, Cook ER, Harper K, Huybers P, et al. Climate Change during and after the Roman Empire: Reconstructing the Past from Scientific and Historical Evidence. J Interdiscip Hist. 2012;43: 169–220. doi:10.1162/JINH_a_00379
59. McMichael AJ. Insights from past millennia into climatic impacts on human health and survival. Proc Natl Acad Sci U S A. 2012;109: 4730–7. doi:10.1073/pnas.1120177109
