# Supplementary material for: Buried in water, burdened by nature—Resilience carried the Iron Age people through Fimbulvinter
Source: PLoS One. 2020 Apr 21;15(4):e0231787. doi: 10.1371/journal.pone.0231787 (PMC7173937; doi:10.1371/journal.pone.0231787)
Supplement: S2 Appendix — (PDF) [file pone.0231787.s002.pdf]

Supporting Information **S2 Appendix. Archaeological contexts of Levänluhta and Käldamäki** for

Buried in water, burdened by nature – Resilience carried the Iron Age people through Fimbulvinter

Corresponding author: Markku Oinonen

Contributors: Kristiina Mannermaa, Markku Oinonen, Santeri Vanhanen, Anna Wessman

S2 Appendix contains: Text, Figures E-F

Text

Eastern Fennoscandia has been populated solely by hunter-gatherer (HG) cultures throughout the Holocene, until the maritime Pitted Ware culture reached Åland archipelago with cultivated barley and wheat ca. 3000 BC[1], and soon afterwards the Corded Ware culture appeared with signs of agropastoralism ca. 2800 BC[2]. The so-called Kiukainen culture emerged ca. 2200 BC inheriting features of the former HG cultures and animal husbandry[3]. Merging of the hunter-gatherer and agricultural livelihoods have thus long traditions within Eastern Fennoscandia – people practice these livelihoods even today to some extent in the Finnish countryside by supplementing agricultural income and commercial diet by minor-scale fishing and hunting.

Natural passageways for people to move have been the waterways (rivers and lakes) and ridges. These routes have mediated the evolvement of communities particularly at the lowlands of Ostrobothnia[4]. Importance of rivers, their estuaries, islands and confluences as mediating Iron Age trade and cultural influences has been particularly emphasized recently within the Northern Ostrobothnia[5,6].

The Southern Ostrobothnian archaeology provides particularly strong evidence already from Bronze Age. At the eastern shores of Gulf of Bothnia, in Ostrobothnia, large amount of cooking pits evidence for extensive seal oil production[7] supporting the trade to flourish already during the Bronze Age[8]. This strongly maritime lifestyle declined during the Iron Age ca. 500 BC onwards[8]. Agropastoral lifestyle started to gain more attraction[4] and population spread to inland along the banks of the largest rivers[9] at the same time the rest of the world was pushed into migratory turmoils. The success of the Ostrobothnian culture culminated during the Migration period (ca. AD 400 – 550) when it became one of the richest within the Eastern Fennoscandia[10]. Prosperity continued into the so-called Merovingian period (ca. AD 550 – 800) but by the beginning of the 9th century the settlement seems slowly to fade away[10–12].

Levänluhta (see Fig 1 in the manuscript, Fig E) located 1 km from Kyrö river is one of the most intriguing Iron Age sites in Finland containing uncremated human remains archaeologically dated to AD 300–800[10]. The excavated finds at Levänluhta consist of unburnt commingled human remains from 98 individuals buried along with artefacts and

animal bones[13]. During the Iron Age this site was a probably a pond or a small lake. Presently, it is a combination of three springs in the middle of the Ostrobothnian fields. The site can be considered either as a long-term collective burial or a cemetery. Since there is water impressively involved within the site, a term of “water burial” is used throughout the paper following the previous literature[14]. It has been archaeologically investigated periodically since the 1800s until the 1980s[10,14]. These excavations have yielded 22 artefact finds, including precious copper alloy brooches, arm rings and other dress implements but also a Provincial Roman copper cauldron[10]. Osteological studies have confirmed that mainly for women and children were buried into Levänluhta[13,15] which is quite uncommon in Finland.

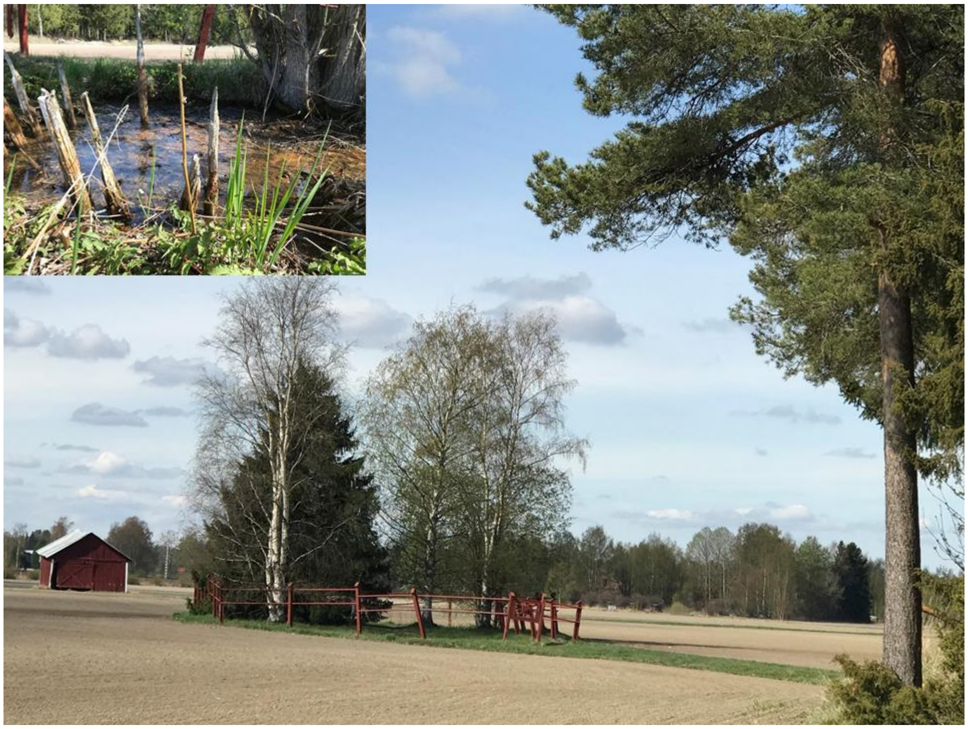

Fig E. Levänluhta site in the middle of a cultivated field in the Southern Ostrobothnian landscape. The insert shows the present appearance of the largest of the three springs within the site. Photos by and with permission of Anna Wessman (2019).

Recent extensive archaeological fieldwork has not revealed any traces of contemporary settlement sites close to the site. The absence of settlements suggests that during its time of use Levänluhta was marginal, clearly outside the central habitation, situated perhaps in a liminal and sacred space, far from the settlement sites[14]. Past interpretations regarding Levänluhta have ranged from it being a place for human sacrifice[12,16–18] to a mass grave for slaves or those who had perished from famine, plague or war[9,11,15,19,20]. Later interpretation has stated that the site is a water burial for women and children who have been selected and buried further away, perhaps due to social or ideological differences[14].

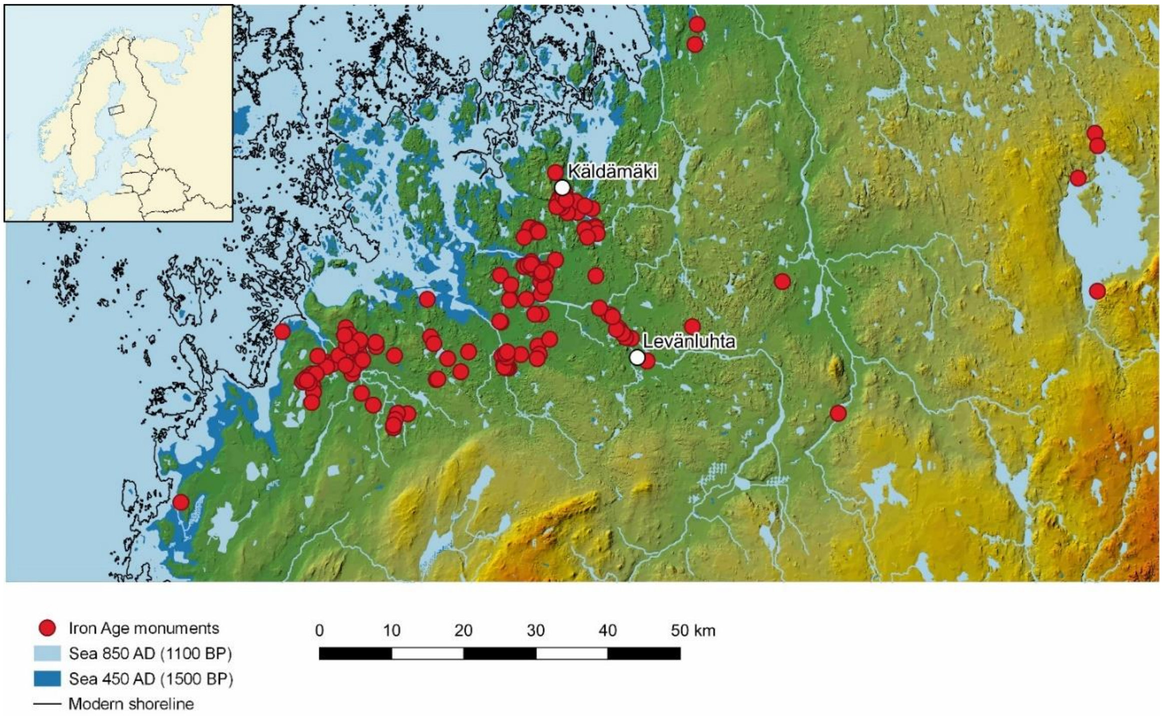

Fig F. Location of the Levänluhta and Käldamäki sites with respect to the seashore at AD 450, AD 850 and present. Shore-level displacement was modelled according to Johan Daniels and Tore Påsse[21]. Locations for Iron Age sites are based on the registry of ancient monuments by the Finnish Heritage Agency, retrieved from: https://www.museovirasto.fi/fi/palvelut-ja-ohjeet/tietojarjestelmat/kulttuuriympariston-tietojarjestelmat/kulttuuriympaeristoen-paikkatietoaineistot. Elevation model and modern shoreline contains data reprinted from the National Land Survey of Finland Topographic Database 05/2019 distributed under CC BY 4.0 licence, with permission of National Land Survey (see https://www.maanmittauslaitos.fi/en/opendata-licence-cc40). Orientation map in the top left corner was made with Natural Earth data (https://www.naturalearthdata.com/).

Another similar, although smaller, site of Käldamäki is located 25 km northwest from Levänluhta in the parish of Vöyri (see Fig 1 in the manuscript, Fig F). The dating of Käldamäki is very similar to Levänluhta but the amount of deceased is only six compared to 98 in Levänluhta[10]. Two cattle and sheep bones dating to Iron Age have been accompanying the dead in Käldamäki[14] but the finds are rare, consisting of a wooden axe shaft, a piece of bronze rod and a fish trap. However it is not certain that these finds belong to the water burial[10].

References

1. Vanhanen S, Gustafsson S, Ranheden H, Björck N, Kemell M, Heyd V. Maritime Hunter-Gatherers Adopt Cultivation at the Farming Extreme of Northern Europe 5000 Years Ago. Sci Rep. 2019;9: 4756. doi:10.1038/s41598-019-41293-z
2. Cramp LJE, Evershed RP, Lavento M, Halinen P, Mannermaa K, Oinonen M, et

al. Neolithic dairy farming at the extreme of agriculture in northern Europe. Proc R Soc B Biol Sci. 2014;281: 20140819–20140819. doi:10.1098/rspb.2014.0819
3. Bläuer A, Kantanen J. Transition from hunting to animal husbandry in Southern, Western and Eastern Finland: new dated osteological evidence. J Archaeol Sci. 2013;40: 1646–1666. doi:10.1016/J.JAS.2012.10.033
4. Holmblad P. Coastal Communities on the Move - House and Polity Interaction in Southern Ostrobothnia 1500 BC – AD 1. Umeå University. 2010. Available: http://www.diva-portal.org/smash/get/diva2:349824/FULLTEXT01.pdf
5. Hakamäki V, Kuusela J-M. Examining the topography and social context of metal age artefact finds in northern Finland. Fennoscandia Archaeol. 2013;XXX: 95–106.
6. Hakamäki V. Seeing behind stray finds - Understanding the late Iron Age settlement of northern Ostrobothnia and Kainuu, Finland. University of Oulu. 2018.
7. Ylimaunu J. Itämeren hylkeenpyyntikulttuurit ja ihminen-hylje-suhde [Baltic seal hunting cultures and human-seal relationship]. Helsinki: Suomalaisen Kirjallisuuden Seura; 2000.
8. Kuusela J-M. Political economy of Bronze- and Iron Age societies in the eastern coast of the Bothnian Bay ca. 1500 BC - 1300 AD. University of Oulu. 2013.
9. Meinander CF. Etelä-Pohjanmaan esihistoria [Prehistory of Southern Ostrobothnia]. Etelä-Pohjanmaan historia I. Helsinki: Etelä-Pohjanmaan historiatoimikunta; 1950.
10. Wessman A. Levänluhta – a place of punishment, sacrifice or just a common cemetery? Fennoscandia Archaeol. 2009;XXVI: 47–71.
11. Seger T. The plague of Justinian and other scourges. Fornvännen. 1982;77: 184–198.
12. Meinander CF. Forntiden i Svenska Österbotten. Svenska Österbottens Historia I. Vasa: Svenska Österbottens landskapsförbund; 1977. pp. 42–43.
13. Formisto T. An osteological analysis of human and animal bones from Levänluhta. University of Stockholm. 1993. Available: https://books.google.fi/books/about/An_Osteological_Analysis_of_Human_and_An.html?id=0WkaNQAACAAJ&redir_esc=y
14. Wessman A, Alenius T, Holmqvist E, Mannermaa K, Perttola W, Sundell T, et al. Hidden and Remote: New Perspectives on the People in the Levänluhta Water Burial, Western Finland (c. ad 300–800). Eur J Archaeol. 2018; 1–24. doi:10.1017/eaa.2017.84
15. Niskanen M. Stature of the Merovingian-period inhabitants from levänluhta, Finland. Fennoscandia Archaeol. 2006;XXIII: 24–36.
16. Hackman A. Ein Opferfund der Völker- wanderungszeit in Finland. In: Haeggström I, editor. Opuscula Archaeologica Oscari Montelio: septuagenario dicata MCMXIII. Holmiae; 1913. pp. 299–316.
17. Kivikoski E. Suomen historia I: Suomen esihistoria [History of Finland I: Prehistory]. Helsinki: WSOY; 1961.
18. Lehtosalo-Hilander P-L. Uhrit ja uskomukset In Keski- ja myöhäisrautakausi [Sacrifices and beliefs In Middle and Late Iron Age]. Suomen Historia I [History of Finland I]. 1984. pp. 303–309.

19. Meinander CF. Förutsättningar för den förhistoriska bebyggelsen i södra Österbotten. Nord Tidskr. 1946; 70–101.
20. Leppäaho J. Kalevala vertailevan muinaistieteen valaisemana [Kalevala in the light of comparative paleosciences]. In: Heporauta F, Haavio M, editors. Kalevala kansallinen aarre. 1949. pp. 49–81.
21. Påsse T, Andersson L. Shore-level displacement in Fennoscandia calculated from empirical data. GFF. 2005;127: 253–268. doi:10.1080/11035890501274253
