# Supplementary material for: Buried in water, burdened by nature—Resilience carried the Iron Age people through Fimbulvinter
Source: PLoS One. 2020 Apr 21;15(4):e0231787. doi: 10.1371/journal.pone.0231787 (PMC7173937; doi:10.1371/journal.pone.0231787)
Supplement: S3 Appendix — (PDF) [file pone.0231787.s003.pdf]

Supporting Information **S3 Appendix. Levänluhta and post-glacial land uplift** for

Buried in water, burdened by nature – Resilience carried the Iron Age people through Fimbulvinter

Corresponding author: Markku Oinonen

Contributors: Markku Oinonen, Santeri Vanhanen

S3 Appendix contains: Text, Figure G

Text

Post-glacial rebound has been slowly altering the landscape within the northern Hemisphere now for a dozen millennia. Close to the center of gravity of the former Fennoscandian ice sheet in Ostrobothnia, Finland, the speed of elevation has been roughly a meter per century, still being 8 mm/year[1]. Shorelines have been receding into distance, archipelagos formed and new land has been born – "in the very front of the people's eyes"[2].

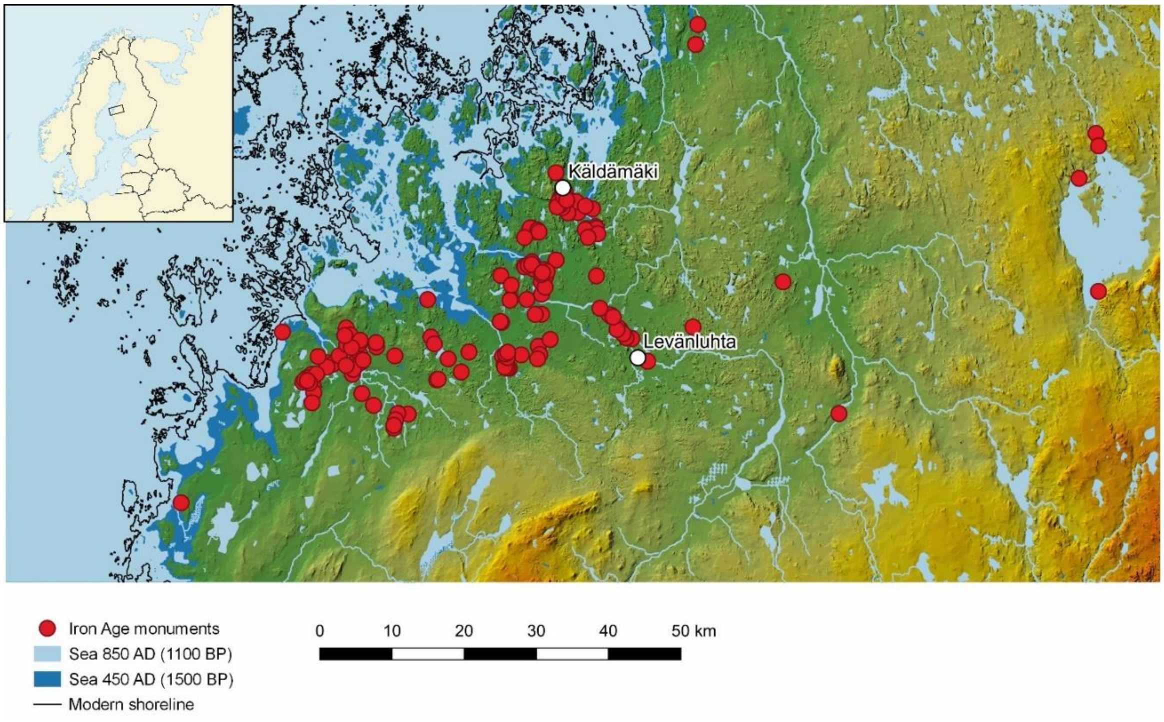

Fig G. Location of the Levänluhta and Käldamäki water burials with respect to the seashore at AD 450, AD 850 and present. Shore-level displacement was modelled according to Johan Daniels and Tore Påsse[3]. Locations for Iron Age sites are based on the registry of ancient monuments by the Finnish Heritage Agency, retrieved from: https://www.museovirasto.fi/fi/palvelut-ja-ohjeet/tietojarjestelmat/kulttuuriymparistonn-

tietojarjestelmat/kulttuuriympaeristoen-paikkatietoaineistot. Elevation model and modern shoreline contains data reprinted from the National Land Survey of Finland Topographic Database 05/2019 distributed under CC BY 4.0 licence, with permission of National Land Survey (see https://www.maanmittauslaitos.fi/en/opendata-licence-cc40). Orientation map in the top left corner was made with Natural Earth data (https://www.naturalearthdata.com/).

During the Bronze Age (1500 – 500 BC) the characteristic feature of the Ostrobothnia was the massive seal oil production that was practiced, particularly, in Laihia region on the shores of vast estuary of Laihia and Kyrö rivers. This activity took advantage of both ice edge and archipelago, the latter formed gradually due to land uplift (Fig G). The marine shoreline was then located near the Levänluhta burial site, which was not used yet, however. The usage started during the 4th century AD (Fig 3 in the manuscript) and – during the first enhanced burial activity at ca. AD 400 - 450 - the site was already located ca. 20 km from the shoreline of the Gulf of Bothnia in Ostrobothnian inland (Fig G). Over the four centuries, the shoreline was receding away 10 - 15 km at the river delta of the Kyrö river, particularly. Thus, the site was never bound to marine shoreline during its use and was an inland site throughout.

References

1. Eronen M, Ristaniemi O. Late quaternary crustal deformation and coastal changes in Finland. Quat Int. 1992;15–16: 175–184. doi:10.1016/1040-6182(92)90045-4
2. Meinander CF. Etelä-Pohjanmaan esihistoria [Prehistory of Southern Ostrobothnia]. Etelä-Pohjanmaan historia I. Helsinki: Etelä-Pohjanmaan historiatoimikunta; 1950.
3. Påsse T, Andersson L. Shore-level displacement in Fennoscandia calculated from empirical data. GFF. 2005;127: 253–268. doi:10.1080/11035890501274253
