# Supplementary material for: Buried in water, burdened by nature—Resilience carried the Iron Age people through Fimbulvinter
Source: PLoS One. 2020 Apr 21;15(4):e0231787. doi: 10.1371/journal.pone.0231787 (PMC7173937; doi:10.1371/journal.pone.0231787)
Supplement: S4 Appendix — (PDF) [file pone.0231787.s004.pdf]

Supporting Information **S4 Appendix. Bone material and isotopic data** for

Buried in water, burdened by nature – Resilience carried the Iron Age people through Fimbulvinter

Corresponding author: Markku Oinonen

Contributors: Laura Arppe, Hervé Bocherens, Heli Etu-Sihvola, Markku Oinonen, Kati Salo

S4 Appendix contains: Text, Figure H-J, Table A-F

Text

There have been two major osteological studies on the Levänluhta bone material. Altogether, the Levänluhta bone material contains ca. 70 kg of human bone material totally disarticulated according to Formisto[1]. The human bone material is from 98 individuals, of which 37% were determined to be below 18 years old (children/juveniles) and 63% above 18 years old (adults). Out of adults, sex ratio was determined as 1/3 being males and 2/3 females. Later on, Markku Niskanen[2] re-determined sexes of 12 adults from male to female and 2 from female to male. Based on these studies, ca. 40% are children/juveniles, ca. 10% adult males and ca. 50% adult females.

Femora forms a large part (ca. 12% in mass) of the bone material probably because they have resisted erosion due to the sturdy bone walls of compact bone[1]. Altogether, material contained 62 pcs of left *femora* out of which 38 pcs (see below) were individually identified. These were chosen under study due to their best representability (ca. 39% of individuals) and thus highest statistics. Previously, 79% of the left *femora* has been considered belonging to adults by Formisto[1].

In this work, new osteological measurements (see Methods) were performed on all the identified left *femora* from Levänluhta and Käldamäki sites (Table A-C). Age estimation of children is easy based on the size of the bones and epiphyseal surfaces. However diaphyseal fragments, when reaching adult size (as juveniles) cannot be age estimated even as even belonging to adult age group, since diaphyseal size is reached already as juvenile and there are no standard procedures for adult age estimations based on the *femur*. Therefore, the ones with visible epiphyseal line were estimated as young adults and the rest that had proximal or distal epiphysis attached to the diaphysis as adults.

Original selection of 39 left *femora* was cross-checked during the project with the result that one of the *femora* (#5) was re-identified as right *femur*. Thus, to avoid analysing the same individual twice, this was left out from the eventual analyses. Slight uncertainty remains also for two femora (#30, 32) that may belong to the same individual as the fragment shape and size allows it. Chronologically and isotopically they are nearly similar. Only #30 has been included in the eventual chronological models and time series data to avoid risk for duplicate measurement of the same individual. The measurements prove 50% (19/38) of the bones belonging to adults in addition to 3 children and 1 juvenile. Of the adults six have been determined as young adults based on the epiphyseal line visibility. Most of the undetermined are either adults or juveniles that have reached adult size. Due to formation and slow regeneration[3] the radiocarbon signal of cortical bone has been deduced to typically reflect the central years of lifespan of a 40-yr old individual[4]. Therefore, we expect the isotopic ratios also to reflect the early adulthood of the measured adults and essentially the whole lifespan of children/juveniles. However, for terrestrial mammals, the isotopic ratios of *femora* cortical bone have been also observed recently to reflect adolescence[5].

Only 9 thighbones have been complete enough for sex estimations in the preceding studies[1,2]. The new measurements performed in this work agree with Formisto by identifying the bone #4 as male. In addition, the bones #15, #18 are suggested being from female and #14, #32 from male. Four adult females have acceptable stable isotopic values measured (#'s 1-3,15). Being a subset of overall broad isotopic distribution (Fig H), they do not stand out from the complementary data (δ^{13}C: t(4)= -0.319, p=0.766; δ^{15}N: t(4)= -1.484, p=0.212; Table F). This all is in agreement with the proposed dominance of females in the data[1,2] and/or small separation of sex-specific isotopic values, as observed - for instance - by Fuller et al[6] for British Iron Age individuals. As the sex-specific differences within isotopic values are typically less than one permille[6], it is concluded that they do not explain the observed grouping and wide scattering in the data. Instead, potential dominance of single sex may even emphasize the dietary differences behind the widely scattered data as the possible sex-induced differences of ca. 1 permille level would be largely avoided.

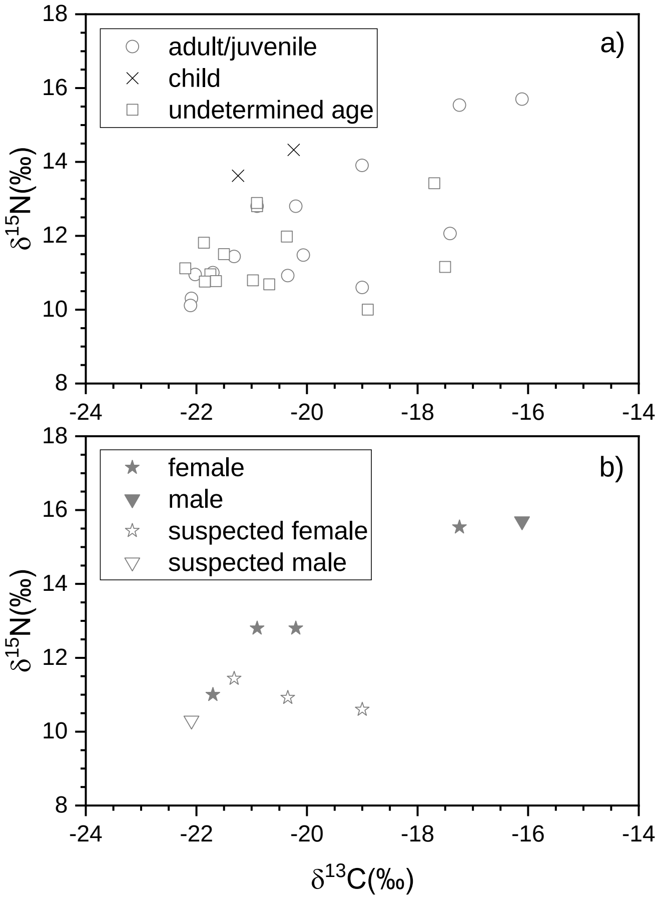

Fig H. a) Age-of-deaths and b) identified sexes (Tables A-C) among the isotopic data (Table 1 in the manuscript) of the studied individuals.

Three individuals have been identified as children (Table B). Two children have acceptable isotopic values: (#33/child 0.5-2.5 yrs, #34/ infant 0-1 yrs). These, having slightly elevated isotopic ratios, stand out from the assumed base population centered at δ^{13}C ~ -21.3 ‰, δ^{15}N ~ 11.4 ‰ (Figs H-J, Table D). This may indicate effects of breastfeeding[6,7]. If taking into account the average effects

of breastfeeding for carbon and nitrogen isotopic values (1 and 2-3‰[7] for C and N, respectively) these individuals would fall within the isotopic distribution of the base population.

The way the identified children are treated affects the quantitative clustering analysis and the forthcoming interpretation of the results. If performing hierarchical cluster analysis on the data without taking into account the potential breastfeeding effect on the children, the data are clustered into four subgroups (Fig I). Instead, by taking the effect into account, one obtains three subgroups (Fig J) as the data points of #1,2,10,25,33,34 are grouped into the larger base population. Following an idea to take all the available information account, we adopt the latter approach and conclude that the data is clustered into three subgroups (LL1,2,3). However, we also note that data points of #1,2,10,21,25 may carry additional information on a distinguished group of population.

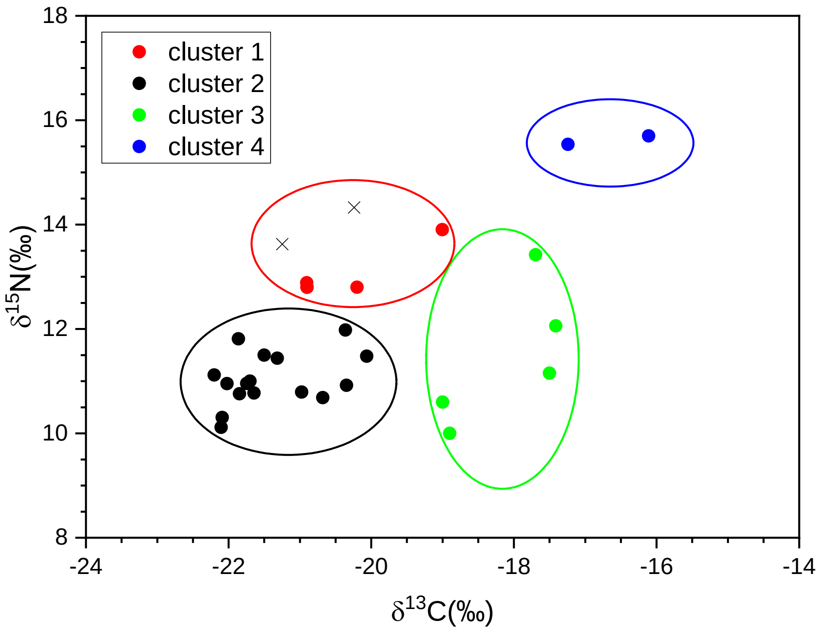

Fig I. Hierarchical cluster analysis on the data by neglecting the potential breastfeeding effect on children.

Reference populations (Fig 2 in the manuscript, Table D) within the Baltic Sea provides an overview of the expected range of isotopic values. Terrestrial influence has been considered being strongest in the Neolithic hunter-gatherer community of Dragby[8] in Uppland, Sweden and within the Bronze Age context of Resmo[9], Öland, Sweden. Terrestrial resources dominate also the diet of medieval Sigtuna individuals[10] with possibly a minor contribution of aquatic resources. Zvejnieki[8] in Latvia stands out as a context with a pronounced use of freshwater resources. In Öland, the Köpingsvik's Neolithic economy has been clearly based on marine resources[9] whereas the Viking Age/Medieval population in Ridanäs, Gotland, Sweden relied on both marine food and agriculture[11].

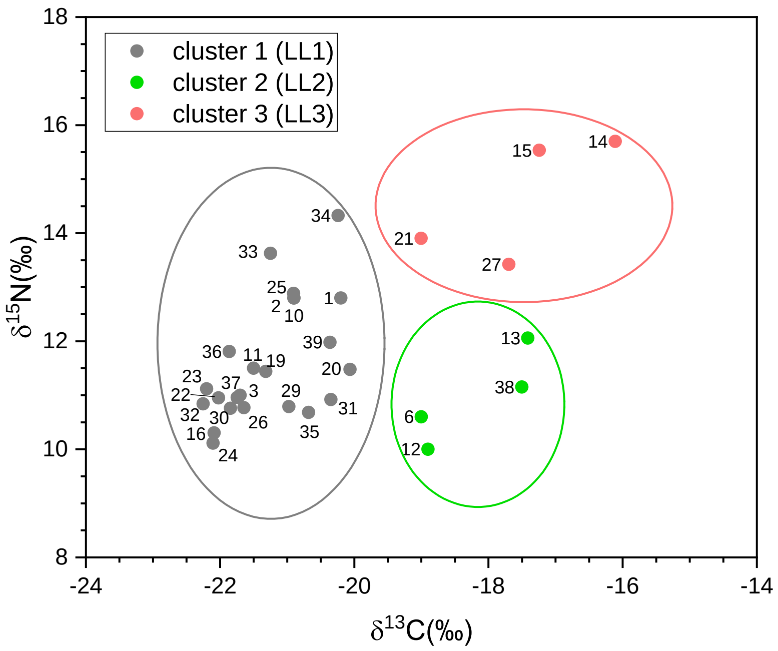

Fig J. Hierarchical cluster analysis on the data by taking into acount the potential breastfeeding effect on children. The data points #33 and #34 are related to individuals identified as children.

Table A. Sample data for the human bones (femora) analysed within this study. FHA = Finnish Heritage Agency, DA = Department of Anatomy, University of Helsinki. The fragmentary research history of the Levänluhta site results in fragmentary coding.

Site	Sample #, k	Code (FHA)	Code (DA)	Code (Hela-xxxx / ETH-xxxxx)	Bone identification	Weight(g)	Notes
LL	1	6367:13	2:13:13 S	2128	excl. frag.	185.5	
LL	2	6367:13	2:13:5 S	2243	excl. frag.	208.4	
LL	3	6367:13	2:13:4 S	2244	excl. frag.	172.8	
LL	4	6367:13	2:13:6 S	2245	excl. frag.	287.3	
LL	5	6367:13	2:13:28 D	2246	diaphysis excl. frag.	118.6	identified as right femur
LL	6	6367:13	2:13:14 S	2251	prox. 2/3 excl. trochanter frag.	168.8	
LL	7	21814:886, 908	908	2252	prox 2/3 excl trochanter frag.	164.5	pieces glued together
LL	8	21926:350, 352, 360, 365, 475, 521, 571	571	2253	prox. diaphysis 2/3 frag.	97.5	pieces glued together
LL	9	6367:13	2:13:15 S	2261	proximal 1/3 frag.	117.5	
LL	10	6367:13	2:13:19 D	2262	prox. diafysis 2/3 frag.	73	
LL	11	6367:13	2:13:36 S	2263	prox. diaphysis frag.	61.8	
LL	12	21926:1502, 1516	1502	2264	prox. diaphysis frag.	56.6	pieces glued together
LL	13		2:13:1 S	3268	excl. trochanter major, medial condyle frag.	233.8	
LL	14		2:13:2 S	3269	excl. frag.	246.1	
LL	15		2:13:7 S	3270	prox. 3/4 frag.	185	
LL	16		2:13:8 S	3271	prox. 3/4 frag. excl. trochanter and caput frag.	202.8	
LL	17		2:13:9 S	3272	prox. 2/3 excl. trochanter frag.	218.3	
LL	18		2:13:10 S	3273	prox. 2/3 excl. trochanter major frag.	152.3	

Site	Sample #, k	Code (FHA)	Code (DA)	Code (Hela-xxxx / ETH-xxxxx)	Bone identification	Weight(g)	Notes
LL	19		2:13:11 S	3274, 57297	prox. 2/3 excl. trochanter major frag.	189.9	
LL	20		2:13:12 S	3275	prox. 2/3 excl caput	168.1	
LL	21		2:13:13 S	3276	diaphysis excl. dist. frag.	144.6	
LL	22		2:13:16 S	3277, 57298	prox. 2/3 frag. excl. caput and trochanter frag.	131.5	
LL	23		2:13:17 S	3278	diaphysis excl. frag.	168	
LL	24		2:13:19 S	3279	prox. 3/4 frag. excl. caput et trochanter	157.6	
LL	25		2:13:21 S	3280	diaphysis frag.	134.1	
LL	26		2:13:24 S	3281, 55271	diaphysis excl. frag	175.6	
LL	27		2:13:25 S	3282	diaphysis excl. dist frag.	138.9	
**LL**	28		2:13:26 S	3283	diaphysis excl. frag.	47.6	
LL	29		2:13:30 S	3284, 57299	diaphysis excl. prox. et dist frag.	136.2	
LL	30		2:13:31 S	3285	dist. diaphysis frag.	77	counterpart of 32?
LL	31		2:13:32 S	3286	prox. 1/3 frag.	101.5	
LL	32		2:13:34 S	3287, 55272	head and neck frag.	25.2	counterpart of 30?
LL	33		2:13:27 S	3288	diaphysis excl. frag.	7.4	
LL	34		2:13:18 S	3289	diaphysis prox. 2/3 frag. excl. frag.	3.6	
LL	35	21926:622, 21926:615, 21926:1452, 22403:8		3290			pieces glued together
LL	36	21814:94		3291	diaphysis and neck frag.	144.1	
LL	37	?????:438		3292	prox. diaphysis 1/2 frag.	89	
LL	38	21814:590, 21814:595		3293	diaphysis prox. 2/3 frag. excl. frag.	96.3	pieces glued together

Site	Sample #, k	Code (FHA)	Code (DA)	Code (Hela-xxxx / ETH-xxxxx)	Bone identification	Weight(g)	Notes
LL	39	21926:1201		3294	diaphysis	172	
KM	40			3295			
KM	41			3296			
KM	42			3297			
KM	43			3298			

Table B. Osteological data on the human bones for age-of-death estimations. Formisto[1] provides the age-at-death distribution of left thighbones as follows: infant(0-1 yr) 1 pc, infans I(0-7 yrs) 9 pcs, infans II (5-14 yrs) 1 pc, infans II/juvenilis (5-24 yrs) 1 pc, juvenilis (10-24 yrs) 1 pc, adult (18 – 79+ yrs) 49 pcs, but do not provide complete codes for identification. Our adopted age-at-death estimations are based on the measurements performed in this work (most LL bones) and on the work of Formisto (KM bones). Explanations for the column titles are provided below the table.

Site	Sample #, k	Age at death[1]	Exostoses in the trochanteric fossa	Enthesophytes	Osteophytes head	Osteophytes distal	Age at death (this work)	Age at death (adopted)
LL	1	adult	1	1	2	2	adult	adult
LL	2	adult	0	0	1	0	adult	adult
LL	3	adult	1	1	-	3	adult	adult
LL	4	adult	0	0	1	1	adult	adult
LL	5	adult	-	0	-	-		
LL	6	adult	0	0	1	-	adult	adult
LL	7	adult	0	0	1	-	adult	adult
LL	8		-	0	-	-		
LL	9	adult	0	0	1	-	young adult	adult
LL	10		-	0	-	-		
LL	11	adult	-	-	-	-		
LL	12		-	1	-	-		
LL	13		-	1	2	1	adult	adult
LL	14		0	0	1	1	adult	adult
LL	15		0	0	1	-	young adult	adult
LL	16		-	0	2	-	adult	adult
LL	17		0	0	1	-	young adult	adult
LL	18		0	1	2	-	adult	adult
LL	19		-	0	1	-	young adult	adult
LL	20		0	0	-	-	adult	adult
LL	21		-	0	-	-	juvenile, trochanter metaphysis	juvenile
LL	22		0	0	-	-	adult	adult

Site	Sample #, k	Age at death[1]	Exostoses in the trochanteric fossa	Enthesophytes	Osteophytes head	Osteophytes distal	Age at death (this work)	Age at death (adopted)
LL	23		-	0	-	-		
LL	24		-	0	-	-	adult	adult
LL	25		-	-	-	-		
LL	26		-	0	-	-		
LL	27		-	0	-	-		
LL	28	child	-	-	-	-	ca.4.5-5.5y	child
LL	29		-	0	-	-		
LL	30		-	0	-	-		
LL	31		0	0	1	-	young adult	adult
LL	32		-	-	1	-	young adult	adult
LL	33	child 0.5-2.5y	-	0	-	-	0.5-2.5 y	child 0.5-2.5y
LL	34	infans I	-	0	-	-	infans I	infans I
LL	35							
LL	36		-	0	-	-		
LL	37		-	0	-	-		
LL	38		-	0	-	-		
LL	39		-	0	-	-		
KM	40	adult						adult
KM	41							
KM	42	adult						adult
KM	43	adult						adult

Exostoses in the trochanteric fossa = osteofytic bone formation (usually in the form of bony spicules) in the trochanteric fossa, that have been also thought as nonmetric variants[12]; 0: no, 1: yes
Enthesophytes = additional bone growth on the muscle attachment sites[13]; 0: no, 1: yes
Osteophytes head = 1: no or slight osteofytic bone formation, 2: osteophytes and porosity, 3: eburnation on the proximal articular surface of the femur

Osteophytes distal = 1: no or slight osteophytes, 2: osteophytes and porosity, 3: eburnation on the distal articular surface of the femur(after Schulz[14]). Usually grade 3 or grade 2 and 3 are counted as osteoporosis

Table C. Osteological data on the human bones for sex estimations. The data are given in millimeters. Our adopted sex estimations are based on the epicondyle breadth and head maximum measurements performed in this work (most LL bones) and on the work of Formisto[1] (KM bones). Explanations[15] for the column titles are provided below the table.

Site	Sample #, k	Sex [1]	Sex [2]	Length max.	Length nat.	Epicond. br.	Head max.	AP subtro-chanter	ML subtro-chanter	AP mid-shaft	ML mid-shaft	mid-shaft circumf.	Sex (this work)
LL	1	F	F	384	381	67.6	38.5	19.3	29.1	22.7	23.1	71	F
LL	2	F	F	363	358	64.6	38.5	22.3	30.8	22.4	24	72	F
LL	3	F	F	388	385	64.5	38	20.6	29.8	23.3	25.5	80	F
LL	4	M	F	423	421	69	45	25.6	30.5	29.2	25.6	82	M
LL	5	F	F	-	-	-	-	22.7	32.5	25	25.8	82	
LL	6	F	F	-	-	-	40.6	22	31.6	24	27.4	78	F?
LL	7	F	F	-	-	-	41.4	23.6	30.1	-	-	-	F?
LL	8			-	-	-	-	-	-	25.3	26.5	79	
LL	9	F	F	-	-	-	38.5	22.1	29.7	-	-	-	F
LL	10			-	-	-	-	21	27.9	24.5	22.2	78	
LL	11	F	F	-	-	-	-	23.3	30.1	-	-	-	
LL	12			-	-	-	-	28.6	35.4	-	-	-	
LL	13			378	377	-	42.2	25.8	34.1	26	25.3	82	?
LL	14			415	413	73.2	43.2	24.6	32	24	25.3	79	M
LL	15			-	-	-	38.5	21.6	28.6	22	23	67	F
LL	16			-	-	-	42.8	23.3	35.5	24.4	28.6	84	M?
LL	17			-	-	-	41.9	23.9	33.9	25.4	29.1	83	?
LL	18			-	-	-	39.6	18.9	29.2	21.7	22.8	73	F
LL	19			-	-	-	40	21.6	31.3	23	25.8	79	F?
LL	20			-	-	-	-	22.4	29.8	23	25.5	76	
LL	21			-	-	-	-	18.1	27.4	22.7	21.1	69	
LL	22			-	-	-	-	19.7	30	20.3	23.2	70	
LL	23			-	-	-	-	24.2	35.9	26.2	30.1	88	
LL	24			-	-	-	-	24.5	31.2	22.9	24	74	

Site	Sample #, k	Sex [1]	Sex [2]	Length max.	Length nat.	Epicond. br.	Head max.	AP subtro-chanter	ML subtro-chanter	AP mid-shaft	ML mid-shaft	mid-shaft circumf.	Sex (this work)
LL	25			-	-	-	-	-	-	24	27.1	88	
LL	26			-	-	-	-	23.8	32.4	26.8	27.9	85	
LL	27			-	-	-	-	21.1	28.1	23.7	25.6	76	
LL	28			235				16	18.6	11.9	13.3	42	
LL	29			-	-	-	-	23.4	31.7	24.6	25.6	77	
LL	30			-	-	-	-	-	-	26.9	23.4	78	
LL	31			-	-	-	40.6	22.8	31.9	-	-	-	F?
LL	32			-	-	-	46.8	-	-	-	-	-	M
LL	33			141	-	-	-	-	-	9.2	9.1	27	
LL	34			-	-	-	-	10.3	9.9	7.4	9.4	34	
LL	35												
LL	36			-	-	-	-	20	30.5	24.2	23.1	72	
LL	37			-	-	-	-	21.9	29.1	22.9	23.6	70	
LL	38			-	-	-	-	22.2	27.1	-	-	-	
LL	39			-	-	-	-	25.5	32.2	28.2	27	80	
KM	40												
KM	41												
KM	42												
KM	43												

Length max. = maximum length of the femur
Length nat. = natural length of the femur
Epicond. br. = maximum breadth of the distal femur on the epicondylar level (F=64.5-67.6 M=69-73.2)
Head max. = maximum breadth of the proximal articular surface (F=38-39.6, F?=40-41.4, ?=41.5-42.5, M?=42.8, M=43.2-46.8)
AP subtrochanter = antero-posterior diameter under the trochanters of femur
ML subtrochanter = medio-lateral diameter under the trochanters of femur
AP mid-shaft = antero-posterior diameter of the mid shaft of femur
ML mid-shaft = medio-lateral diameter of the mid shaft of femur
mid-shaft circumf. = circumference of the midshaft measured with measuring tape

Table D. Comparison of reference populations within the Baltic Sea basin to Levänluhta and Käldamäki populations. Differences of standard deviations (σ's) between Levänluhta and the site populations are given as $\sigma_{LL}/\sigma_{site}$, C and $\sigma_{LL}/\sigma_{site}$, N for carbon and nitrogen isotopic ratios, respectively.

Site population	N	$\delta^{13}C$(‰)	σ	$\delta^{15}N$(‰)	σ	Source	$\sigma_{LL}/\sigma_{site}$, C	$\sigma_{LL}/\sigma_{site}$, N
Levänluhta all	30	-20.3	1.7	11.9	1.5	this work		
Levänluhta LL1	22	-21.2	0.7	11.6	1.1	this work	2.4	1.4
Levänluhta LL2	4	-18.2	0.9	11.0	0.9	this work	2.0	1.7
Levänluhta LL3	4	-17.5	1.2	14.6	1.1	this work	1.4	1.3
Käldamäki KM	4	-20.1	0.8	11.9	0.8	this work	2.1	2.0
Dragby, Uppland	10	-19.7	0.2	10.3	0.3	[8]	2.9	2.4
Köpingsvik, Öland	26	-14.4	0.6	16.7	0.6	[9]	1.4	1.5
Resmo BA, Öland	10	-19.9	0.4	10.1	0.5	[9]	2.4	1.1
Ridanäs, Gotland	10	-17.2	1.2	11.1	1.0	[11]	7.2	4.8
Sigtuna, Uppland	72	-21.0	0.7	12.4	1.4	[10]	4.1	3.4
Zvejnieki, Latvia	6	-22.3	1.9	11.8	0.7	[8]	0.9	2.1

Table E. Quantified differences among the isotopic data of the observed population clusters and reference populations defined as squared euclidean distance (d^2) of the bivariant data ($\delta^{13}C$, $\delta^{15}N$). The squared Euclidean distance is a metrics used in the adopted clustering analyses and thus is consistent to clusters shown in Fig 2 and in Table 1.

a) $d^2(\delta^{13}C,\delta^{15}N)$	LL1	LL2	LL3	KM	Dragby	Köpingsvik	Resmo	Ridanäs	Sigtuna	Zvejnieki
LL1	NA	9.820	23.883	1.474	4.055	74.463	3.796	16.633	0.775	1.022
LL2		NA	14.073	4.741	2.498	47.698	3.659	0.964	10.088	17.172
LL3			NA	14.179	23.105	14.098	26.188	12.756	17.575	30.861
KM				NA	2.808	55.888	3.339	9.226	0.998	4.547
Dragby					NA	68.401	0.117	6.453	6.063	8.830
Köpingsvik						NA	74.044	39.746	63.153	86.647
Resmo							NA	8.193	6.290	8.139
Ridanäs								NA	16.216	25.840
Sigtuna									NA	1.874
Zvejnieki										NA

Table F. Quantified differences among the isotopic data based on two-sample T test with unequal variance assumed. Identified children (#33,34) were omitted. N = number of individuals, mean = mean value of a distribution, σ= standard deviation of a distribution, d.o.f. = degrees of freedom, t = t value provided by the T test, p = probability of the given distributions having the same mean value. Hypothesis of statistically similar distributions is rejected if p<0.05.

Comparison	Variable	N_1	$mean_1$	σ_1	N_2	$mean_2$	σ_2	d.o.f.	t	p
1) others vs 2) females	$\delta^{13}C$	24	-20.3	1.8	4	-20.0	1.9	4	-0.319	0.766
1) others vs 2) females	$\delta^{15}N$	24	11.6	1.3	4	13.0	1.9	4	-1.484	0.212
1) LL1 vs 2) LL2	$\delta^{13}C$	20	-21.3	0.7	4	-18.2	0.9	4	-6.675	0.003
1) LL1 vs 2) LL2	$\delta^{15}N$	20	11.4	0.9	4	11.0	0.9	4	0.921	0.409
1) LL1 vs 2) LL3	$\delta^{13}C$	20	-21.3	0.7	4	-17.5	1.2	3	-6.077	0.009
1) LL1 vs 2) LL3	$\delta^{15}N$	20	11.4	0.9	4	14.6	1.1	4	-5.367	0.006
1) LL1 vs 2) KM	$\delta^{13}C$	20	-21.3	0.7	4	-20.1	0.8	4	-2.619	0.059
1) LL1 vs 2) KM	$\delta^{15}N$	20	11.4	0.9	4	11.9	0.8	5	-1.286	0.255
1) LL2 vs 2) LL3	$\delta^{13}C$	4	-18.2	0.9	4	-17.5	1.2	5	-0.937	0.392
1) LL2 vs 2) LL3	$\delta^{15}N$	4	11.0	0.9	4	14.6	1.1	6	-5.111	0.002
1) pre AD536 vs 2) AD536-570	$\delta^{13}C$	10	-19.9	2.2	3	-21.1	0.9	9	-1.415	0.191
1) pre AD536 vs 2) AD536-570	$\delta^{15}N$	10	12.1	2.0	3	10.7	0.3	10	-2.187	0.054
1) pre AD536 vs 2) AD536-710	$\delta^{13}C$	10	-19.9	2.2	13	-20.6	1.4	14	-0.833	0.419
1) pre AD536 vs 2) AD536-710	$\delta^{15}N$	10	12.1	2.0	13	11.6	1.2	14	-0.751	0.465
1) pre AD536 vs 2) AD600-660	$\delta^{13}C$	10	-19.9	2.2	7	-20.5	1.7	15	-0.663	0.517
1) pre AD536 vs 2) AD600-660	$\delta^{15}N$	10	12.1	2.0	7	11.9	1.4	15	-0.301	0.768
1) LL1, pre AD536 vs 2) LL1, AD536-570	$\delta^{13}C$	6	-21.5	0.5	3	-21.1	0.9	3	0.704	0.532
1) LL1, pre AD536 vs 2) LL1, AD536-570	$\delta^{15}N$	6	11.1	0.3	3	10.7	0.3	4	-1.967	0.121
1) LL1, pre AD536 vs 2) LL1, AD536-710	$\delta^{13}C$	6	-21.5	0.5	10	-21.2	0.7	13	1.074	0.303
1) LL1, pre AD536 vs 2) LL1, AD536-710	$\delta^{15}N$	6	11.1	0.3	10	11.4	1.1	11	0.805	0.438
1) LL1, pre AD536 vs 2) LL1, AD600-660	$\delta^{13}C$	6	-21.5	0.5	5	-21.5	0.6	8	0.140	0.892
1) LL1, pre AD536 vs 2) LL1, AD600-660	$\delta^{15}N$	6	11.1	0.3	5	11.4	1.3	4	0.575	0.596
1) LL1, pre AD536 vs 2) LL1, AD620-680	$\delta^{13}C$	6	-21.5	0.5	6	-21.1	0.6	10	1.410	0.189
1) LL1, pre AD536 vs 2) LL1, AD620-680	$\delta^{15}N$	6	11.1	0.3	6	12.0	1.0	6	2.044	0.087
1) LL1, pre AD536 vs 2) LL1, AD640-700	$\delta^{13}C$	6	-21.5	0.5	5	-21.1	0.6	8	1.151	0.283
1) LL1, pre AD536 vs 2) LL1, AD640-700	$\delta^{15}N$	6	11.1	0.3	5	11.8	1.0	5	1.496	0.195

Comparison	Variable	N_1	$mean_1$	σ_1	N_2	$mean_2$	σ_2	d.o.f.	t	p
1) LL1, others 2) LL1, AD600-660	$\delta^{13}C$	15	-21.2	0.7	5	-21.5	0.6	9	0.898	0.392
1) LL1, others 2) LL1, AD600-660	$\delta^{15}N$	15	11.4	0.7	5	11.4	1.3	5	-0.125	0.905

References

1. Formisto T. An osteological analysis of human and animal bones from Levänluhta. University of Stockholm. 1993. Available: https://books.google.fi/books/about/An_Osteological_Analysis_of_Human_and_An.html?id=0WkaNQAACAAJ&redir_esc=y
2. Niskanen M. Stature of the Merovingian-period inhabitants from levänluhta, Finland. Fennoscandia Archaeol. 2006;XXIII: 24–36.
3. Manolagas SC, Jilka RL. Bone Marrow, Cytokines, and Bone Remodeling — Emerging Insights into the Pathophysiology of Osteoporosis. Epstein FH, editor. N Engl J Med. 1995;332: 305–311. doi:10.1056/NEJM199502023320506
4. Calcagnile L, Quarta G, Cattaneo C, D'Elia M. Determining 14C Content in Different Human Tissues: Implications for Application of 14C Bomb-Spike Dating in Forensic Medicine. Radiocarbon. 2013;55: 1845–1849. doi:10.1017/S003382220004875X
5. Matsubayashi J, Tayasu I. Collagen turnover and isotopic records in cortical bone. J Archaeol Sci. 2019;106: 37–44. doi:10.1016/J.JAS.2019.03.010
6. Fuller BT, Molleson TI, Harris DA, Gilmour LT, Hedges REM. Isotopic Evidence for Breastfeeding and Possible Adult Dietary Differences from Late/Sub-Roman Britain. Am J Phys Anthropol. 2006;129: 45–54. doi:10.1002/ajpa.20244
7. Fuller BT, Fuller JL, Harris DA, Hedges REM. Detection of breastfeeding and weaning in modern human infants with carbon and nitrogen stable isotope ratios. Am J Phys Anthropol. 2006;129: 279–293. doi:10.1002/ajpa.20249
8. Eriksson G, Lidén K. Dietary life histories in Stone Age Northern Europe. J Anthropol Archaeol. 2013;32: 288–302. doi:10.1016/j.jaa.2012.01.002
9. Eriksson G, Linderholm A, Fornander E, Kanstrup M, Schoultz P, Olofsson H, et al. Same island, different diet: Cultural evolution of food practice on Öland, Sweden, from the Mesolithic to the Roman Period. J Anthropol Archaeol. 2008;27: 520–543. doi:10.1016/J.JAA.2008.08.004
10. Kjellström A, Storå J, Possnert G, Linderholm A. Dietary patterns and social structures in medieval Sigtuna, Sweden, as reflected in stable isotope values in human skeletal remains. J Archaeol Sci. 2009;36: 2689–2699. doi:10.1016/j.jas.2009.08.007
11. Kosiba SB, Tykot RH, Carlsson D. Stable isotopes as indicators of change in the food procurement and food preference of Viking Age and Early Christian populations on Gotland (Sweden). J Anthropol Archaeol. 2007;26: 394–411. doi:10.1016/j.jaa.2007.02.001
12. Stirland A. Femoral non-metric traits reconsidered. Anthropologie. 1996; 249–252.
13. Salo K. HEALTH IN SOUTHERN FINLAND : Bioarchaeological analysis of 555 skeletons excavated from nine cemeteries (11th -19th century AD). University of Helsinki. 2016. Available: http://urn.fi/URN:ISBN:978-951-51-2176-9
14. Schultz M. Paläopathologische diagnostik. In: Knussmann R, editor. Anthropologie Handbuch der vergleichenden Biologie des Menschen. Stuttgart: Gustav Fischer Verlag; 1988. pp. 480–496.
15. Buikstra JE, Ubelaker DH. Standards for Data Collection from Human Skeletal Remains : Poceedings of a Seminar at the Field Museum of Natural History.

Aftandilian D, editor. Arkansas Archeological Survey research series. Arkansas Archeological Survey Research; 1994.
