# Supplementary material for: Buried in water, burdened by nature—Resilience carried the Iron Age people through Fimbulvinter
Source: PLoS One. 2020 Apr 21;15(4):e0231787. doi: 10.1371/journal.pone.0231787 (PMC7173937; doi:10.1371/journal.pone.0231787)
Supplement: S5 Appendix — (PDF) [file pone.0231787.s005.pdf]

Supporting Information **S5 Appendix. Isotopic baseline** for

Buried in water, burdened by nature – Resilience carried the Iron Age people through Fimbulvinter

Corresponding author: Markku Oinonen

Contributors: Laura Arppe, Heli Etu-Sihvola, Maria Lahtinen, Markku Oinonen

S5 Appendix contains: Text, Table G-J

Text

The data for the isotopic baseline consist of food and food-group (terrestrial resources: TR, freshwater animals: FA and marine animals: MA) specific isotopic data (Table G, H) and isotopic offsets (Table I). The isotopic data has been mostly obtained by collecting the existing isotopic data into the public δIANA database[1] established for paleodietary research in the Nordic areas and by new measurements. Marine baseline data were separated according to geographical area of the Baltic basin to mimick the different carbon origin: Bothnian Bay i.e. > 63° latitude (later on 63-66°N or *Bothnia*), Baltic Sea proper (56-63°N or *Baltic*) or from the whole region (56-63°N or *total*). This selection affects both the isotopic baseline and the estimated maximal marine reservoir effect (MRE). TR data includes literature values on sites above 50° latitude to allow for taking into account the multitude of food types included (plants, animals, dairy products), as such information is still limited at higher latitudes. Freshwater signal was assumed to be local and thus available eastern Fennoscandian fish flesh values were used. The flesh values were not lipid corrected due to small effect estimated[2]. All $\delta^{13}C$ values of modern samples were corrected for the anthropogenic carbon dioxide i.e. the Suess effect[3] by +1.5‰, defined as the year ~AD 2010 estimate[4]. $\delta^{15}N$ values of all the charred samples were corrected by -1‰[5] either already in literature cited[6,7] or within this work[8]. The average values given include these corrections. Eventually, the food fraction (Protein / Energy) -specific isotopic values for food groups were obtained (Table J) by using offsets (Table I) to convert the values to edible macronutrient values and by simple averaging them within each food group. The simple averaging was adopted to avoid biases within the dietary modellings.

Based on the isotopic baseline data, one can access the issue of high $\delta^{13}C$ values of the LL2 subgroup. The LL2 subgroup differs from the LL1 by carbon isotopic ratios ($t(4)= -6.779$, $p=0.002$) and from the LL3 subgroup by nitrogen isotopic ratios ($t(6)= -5.111$, $p=0.002$) (Table F in S4 Appendix). Our dietary modelling provides a reasonable interpretation for these separations and indicate, in particular, negligible freshwater influence in LL2. The assumed average $\delta^{13}C$ value for edible protein is -25.8 ± 0.7‰ (Table J). We have adopted this as an average of all the available terrestrial food proteins. The relatively low value means that the dietary modelling interprets the high carbon isotopic values of LL2 being largely due to marine influence. However, we are aware that the average isotopic baseline for TR do not necessarily resolve the underlying fine structure on, particularly, the high carbon isotopic ratios observed for LL2.

There could be two alternative possibilities that could yield to high carbon isotopic ratios for LL2 human bones. The first is consumption of reindeer meat. Reindeer (*Rangifer tarandus*) is known to possess slightly higher $\delta^{13}C$ values (-19.7 ± 0.7‰) compared to typical C3-plant eating herbivores due to lichen as a winter forage[9,10] (Table H). The other is consumption of flesh of domesticated animals of Gotland: high $\delta^{13}C$ values (-16.6 ± 0.5‰) have been

observed within the Viking Age context of Ridanäs, Gotland[11]. This may indicate use of aquatic plants as a fodder. In addition to assimilating atmospheric CO_2, aquatic plants may use HCO_3^- from water[12] allowing for dissolved inorganic carbon, with high $\delta^{13}C$, to be fixed into plant structure. The sources for such carbon would be rich limestone reservoirs of Gotland and Baltic countries, resulting also in the high MREs found within the areas[13]. We have integrated both reindeer and Gotland domesticated animals into our isotopic baseline of TR (Table H) by averaging within and between species and thus their weight is small within our standard dietary modelling. To understand whether these scenarios would be realistic, dietary modellings were also performed on the LL2 individuals with assumed 30, 50 and 100% fractions of reindeers or, alternatively, Gotland domesticated animals solely contributing to the TR baseline. In addition, for the reindeer scenario being very local, we assumed potential marine influence coming from the Bothnian Bay (*Bothnia*) and for Gotland scenario, as non-local, we assumed it from the Baltic Sea (*Baltic*). As an example, according to the modelling of individual #38, it seems that this would lead to very low marine (2-11%) and very high terrestrial (86-94%) food group fractions for Gotland scenario. Instead, if just applying the normal *Baltic* scenario with averaged isotopic baseline for LL2, one obtains 19% and 79% for MA and TR, respectively. The latter seems more realistic as the selected Viking Age population in Gotland was previously estimated, although not modelled, to consume nearly equally marine and terrestrial resources[11]. Assuming reindeer scenario one obtains very high protein consumptions of 68-78% for #38. Although this is partly explained by the low fat content of reindeer meat and high-protein fish meat, such protein fractions are still unrealistically high and would possibly lead to fatal health effects[14]. Therefore, we exclude both pure reindeer and pure Gotland scenarios and adopt the averaging approach to obtain the isotopic baseline to eventually model the diets of LL2 individuals.

Table G. Animal bone collagen isotopic data measured within this study. All the samples have been found from the Levänluhta burial except three from Käldamäki, marked with # in the second column. NM = National Museum, UH = University of Helsinki, UT = University of Tübingen. The adopted isotopic data were obtained by averaging of the UH and UT results. Tissues from which the analyses have been performed are provided in the column "Species" as * = bone, † = tooth. The corresponding radiocarbon dates, made by the Laboratory of Chronology at UH (see Methods), have been given in Wessman et al (2018)[15]. The food group in which the data is used within the isotopic baseline are specified in the column "Food group".

Species	NM code	RA (BP)	σ	C-%	N-%	C/N	$\delta^{13}C$(‰) UH	$\delta^{15}N$(‰) UH	$\delta^{13}C$(‰) UT	$\delta^{15}N$(‰) UT	$\delta^{13}C$(‰) adopt.	$\delta^{15}N$(‰) adopt.	Food group	Source
*Anser sp.**	21926:915	1510	26	43.7	15.1	3.4	-17.3	15.0	-17.2	15.1	-17.2	15.1		this work, [15]
*Bos taurus**	21926:1199	418	26	40.3	13.0	3.6	-22.9	6.5	-22.4	6.3	-22.6	6.4	TR	this work, [15]
*Bos taurus**	6373:1447	NA	NA	43.5	15.7	3.2	-22.2	4.6	-22.0	4.6	-22.1	4.6	TR	this work
*Bos taurus**	10202:4 or 10438:14#	1748	28	40.2	14.5	3.2	-20.8	6.1	-20.6	6.3	-20.7	6.2	TR	this work, [15]
*Bos taurus**	21926:1744	74	25	42.6	15.0	3.3	-22.0	4.3	-21.8	4.7	-21.9	4.5	TR	this work, [15]
*Bos taurus**	2996:1862	196	25	40.6	14.6	3.3	-21.7	5.5	-21.5	5.7	-21.6	5.6	TR	this work, [15]
*Canis familiaris**	21814:28	371	26	41.2	14.8	3.3	-19.6	10.7	-19.4	11.2	-19.5	11.0		this work, [15]
Equus caballus†	21814:584	593	29	37.7	13.1	3.4	-22.9	4.3	-22.6	4.3	-22.8	4.3	TR	this work, [15]
Equus caballus†	22395:43	382	25	39.6	14.0	3.3	-23.0	5.6	-22.5	5.5	-22.7	5.5	TR	this work, [15]
*Equus caballus**	21814:23	555	29	40.0	14.2	3.3	-22.5	5.8	-22.3	5.9	-22.4	5.9	TR	this work, [15]
Equus caballus†	21814:525	572	29	40.7	14.7	3.2	-22.9	5.0	-22.6	5.3	-22.8	5.2	TR	this work, [15]
Equus caballus†	2441:5	585	28	40.7	14.4	3.3	-22.9	7.4	-22.5	7.2	-22.7	7.3	TR	this work, [15]
*Equus caballus**	10202:4 or 10438:14#	117	25	38.9	14.0	3.2	-22.2	7.6	-22.0	8.1	-22.1	7.8	TR	this work, [15]

Equus caballus†	6111:7.701	330	27	38.5	13.9	3.2	-22.8	5.6	-22.5	5.8	-22.6	5.7	TR	this work, [15]
*Equus caballus**	6373:15.14 17, 1420	NA	NA	37.0	13.0	3.3	-22.4	7.9	-22.1	7.9	-22.3	7.9	TR	this work
*Equus caballus**	21814:526	595	26	42.3	14.8	3.3	-22.9	5.6	-22.7	5.8	-22.8	5.7	TR	this work, [15]
*Gallus domesticus**	21926:345	1234	27	41.6	14.4	3.4	-22.2	12.1	-21.9	12.3	-22.1	12.2	TR	this work, [15]
*Ovis aries**	10202:4 or 10438:14#	1649	27	40.7	13.8	3.4	-21.6	7.1	-21.5	7.4	-21.6	7.2	TR	this work, [15]
*Ovis aries**	21926: 612	136	25	40.5	14.4	3.3	-22.0	5.9	-21.8	6.0	-21.9	6.0	TR	this work, [15]

Table H. Baseline isotopic data used within this work. The isotopic data has been mostly obtained through the public δIANA database[1] and the original references are listed in the column "Source".

Species/sample	Taxon	Tissue	$\delta^{13}C$(‰)	σ	N	$\delta^{15}N$(‰)	σ	N	Food group	Source
Barley	*Hordeum vulgare*	bulk seed	-24.1	2.0	23	3.3	1.4	22	TR	[6,8]
Blackberries	*Rubus fruticosus*	bulk berries	-23.3	1.2	3	-1.5	2.4	3	TR	[16]
Brown bear	*Ursus arctos*	bone collagen	-20.6	0.7	5	5.5	2.1	5	TR	[1,17]
Cattle	*Bos taurus*	bone collagen	-21.8	0.6	46	4.9	1.2	46	TR	this work, [7,18–25]
Charred grains		charred seeds	-24.4	1.4	20	4.5	1.2	20	TR	[7]
Common wheat	*Triticum aestivum*	bulk seed	-22.2	1.1	21	1.7	1.4	20	TR	[8]
Crowberry	*Empetrum nigrum*	bulk berries	-24.7	NA	1	NA	NA	NA	TR	[16]
Durum wheat	*Triticum durum*	bulk seed	-25.0	0.5	9	4.5	1.2	9	TR	[6]
Einkorn wheat	*Triticum monococcum*	bulk seed	-24.1	0.4	8	4.6	0.4	8	TR	[6]
Elk	*Alces alces*	bone collagen	-21.7	0.5	24	4.4	1.6	24	TR	[1,26]
Emmer wheat	*Triticum dicoccum*	bulk seed	-24.1	0.8	29	3.8	1.6	29	TR	[6]
Grouse	*Tetrao sp.*	bone collagen	-22.2	0.5	9	3.5	1.8	9	TR	[1,24]
Hare	*Lepus sp.*	bone collagen	-23.5	0.8	16	3.7	2.1	16	TR	[1,18,24,27]
Horse	*Equus sp.*	bone collagen	-22.7	0.3	11	5.8	1.3	11	TR	this work, [18,25]
Labrador tea	*Rhododendron groenlandicum*	bulk plant	-25.6	1.0	3	-3.6	2.5	3	TR	[16]
Lentil	*Lens culinaris*	bulk seed	-23.1	NA	1	3.5	NA	1	TR	[6]
Milk (organic)		milk protein	-24.0	0.8	60	4.8	0.3	60	TR	[28]
Pea	*Pisum sativum*	bulk seed	-23.9	0.2	2	2.5	1.9	2	TR	[6]
Pig	*Sus sp.*	bone collagen	-21.7	0.6	21	7.3	2.4	21	TR	[7,18–21,24,25,27,29]
Plants		bulk plant	-25.7	1.5	24				TR	[30]
Pond plants		bulk plant	-29.5	NA	1	-0.6	NA	1	TR	[16]
Reindeer	*Rangifer tarandus*	bone collagen	-19.7	0.7	17	5.2	2.1	17	TR	[1,17,24]
Roots	*Hedysarum alpinum (typically)*	bulk plant	-25.7	0.4	2	-1.6	1.2	2	TR	[16]
Salmonberries	*Rubus spectabilis*	bulk berries	-23.8	0.9	5	1.1	1.0	5	TR	[16]

Species/sample	Taxon	Tissue	$\delta^{13}C$(‰)	σ	N	$\delta^{15}N$(‰)	σ	N	Food group	Source
Sheep/goat	*Ovis/Capridae*	bone collagen	-21.6	0.5	34	5.8	1.7	34	TR	this work, [7,17–19,23–25]
Sour dock	*Rumex arcticus*	bulk plant	-27.4	NA	1	2.8	NA	1	TR	[16]
Stinkweed	*Artemisia tilesii*	bulk plant	-27.8	NA	1	-2.7	NA	1	TR	[16]
Bream	*Abramis brama*	flesh	-26.0	2.1	11	13.3	1.4	11	FA	[2]
Burbot	*Lota lota*	flesh	-29.2	1.6	16	17.6	0.9	16	FA	[2]
Perch	*Perca fluviatilis*	flesh	-28.2	1.9	99	14.5	1.9	99	FA	[2,31]
Pike	*Esox lucius*	flesh	-28.7	0.9	12	16.7	0.7	12	FA	[2]
Pike-perch	*Sander lucioperca*	flesh	-29.4	0.6	20	17.2	0.5	20	FA	[2]
Roach	*Rutilus rutilus*	flesh	-27.2	1.7	113	12.8	1.5	113	FA	[2,31]
Ruffe	*Gymnocephalus cernua*	flesh	-32.8	2.7	3	14.9	1.5	3	FA	[32]
Whitefish	*Coregonus lavaretus*	flesh	-30.6	1.1	17	14.5	0.9	17	FA	[2]
Atlantic cod	*Gadus morhua*	bone collagen	-14.6	1.2	53	13.3	1.9	53	MA_{Baltic}	[33–36]
Baltic herring	*Clupea harengus membras*	bone collagen	-14.9	0.4	2	9.5	0.9	2	MA_{Baltic}	[33,34]
Baltic herring	*Clupea harengus membras*	flesh	-21.3	0.9	9	NA	NA	NA	$MA_{Bothnia}$	[37]
Burbot	*Lota lota*	flesh	-20.9	1.8	5	NA	NA	NA	$MA_{Bothnia}$	[37]
Grey seal	*Halichoerus grypus*	bone collagen	-16.1	0.8	11	13.8	0.6	11	MA_{Baltic}	[1,33,34]
Grey seal	*Halichoerus grypus*	flesh	-18.7	1.1	59	13.8	0.9	59	$MA_{Bothnia}$	[38]
Harbour porpoise	*Phocoena phocoena*	bone collagen	-14.9	0.6	6	12.2	0.5	6	MA_{Baltic}	[33,34]
Harp seal	*Pagophilus groenlandicus*	bone collagen	-16.3	0.6	15	13.6	1.0	15	MA_{Baltic}	[33,34,39]
Perch	*Perca fluviatilis*	bone collagen	-14.1	NA	1	9.6	NA	1	MA_{Baltic}	[33]
Perch	*Perca fluviatilis*	flesh	-20.6	0.8	3	NA	NA	NA	$MA_{Bothnia}$	[37]
Pike	*Esox lucius*	bone collagen	-11.7	0.8	8	11.3	0.6	8	MA_{Baltic}	[33,34]
Ringed seal	*Pusa/Phoca hispida*	bone collagen	-16.4	1.1	11	12.4	1.1	11	MA_{Baltic}	[1,24,33,34,39]
Ringed seal	*Pusa/Phoca hispida*	bone collagen	-17.9	1.4	6	13.1	0.6	6	$MA_{Bothnia}$	[1,24]
Ringed seal	*Pusa/Phoca hispida*	flesh	-19.4	0.8	46	13.2	0.5	46	$MA_{Bothnia}$	[38,40]
Ruffe	*Gymnocephalus cernuus*	flesh	-19.6	0.7	5	NA	NA	NA	$MA_{Bothnia}$	[37]

Species/sample	Taxon	Tissue	$\delta^{13}C$(‰)	σ	N	$\delta^{15}N$(‰)	σ	N	Food group	Source
Salmon	*Salmo salar*	flesh	-18.3	0.5	10	NA	NA	NA	$MA_{Bothnia}$	[37]
Smelt	*Osmerus eprlanus*	flesh	-20.3	0.5	7	NA	NA	NA	$MA_{Bothnia}$	[37]
Vendace	*Coregonus albula*	flesh	-21.5	0.4	5	NA	NA	NA	$MA_{Bothnia}$	[37]
Whitefish	*Coregonus lavaretus*	flesh	-20.4	0.8	15	NA	NA	NA	$MA_{Bothnia}$	[37]

Table I. Offsets of isotopic values used in this work. TR = Terrestrial resources, FA = Freshwater animals, MA = Marine animals. The food group and type-specific offsets were used to convert the literature and the measured species-specific reference values of modern/ancient plant, flesh or bone collagen to edible macronutrient values. Uncertainties of offsets has been typically adopted as 0.5‰. The diet-to-collagen offset values (the two lowermost rows) that are taken into account in FRUITS modellings through the model code with 0.2 and 0.5‰ uncertainties[7,41] for carbon and nitrogen, respectively.

Applied to	Corrects for	Correction	Source
TR, plants	$\delta^{13}C$, Bulk to protein	-2	[42]
TR, plants	$\delta^{13}C$, Bulk to carbohydrates	0.5	[42]
TR, plants	$\delta^{13}C$, Protein to lipids	-7	[43,44]
TR, animals	$\delta^{13}C$, Bone collagen to flesh protein	-2	[41,42]
TR, animals	$\delta^{15}N$, Bone collagen to flesh protein	2	[7,41]
TR, animals	$\delta^{13}C$, Flesh protein to flesh lipids	-8	[45]
TR, dairy products	$\delta^{13}C$, Protein to lipids	-2.5	[28]
TR, dairy products	$\delta^{13}C$, Protein to carbohydrates	0	[46]
FA, MA	$\delta^{13}C$, Bone collagen to flesh protein	-1	[41]
FA, MA	$\delta^{15}N$, Bone collagen to flesh protein	2	[41]
FA, MA	$\delta^{13}C$, Flesh protein to flesh lipids	-7	[43,44]
All modern samples	$\delta^{13}C$, Modern to ancient (Suess effect)	1.5	[4,47] (modern assumed as ca. AD2010)
All charred grains	$\delta^{15}N$, Charred to non-charred	-1	[7]
Assumed adults (in FRUITS)	$\delta^{13}C$, Diet to collagen offset	4.8	[7,41,48]
Assumed adults (in FRUITS)	$\delta^{15}N$, Diet to collagen offset	5.5	[41]
Identified children (in FRUITS)	$\delta^{13}C$, Diet to collagen offset	+1 i.e. 5.8	[49,50]
Identified children (in FRUITS)	$\delta^{15}N$, Diet to collagen offset	+2.5 i.e. 8.0	[49,50]

Table J. Food-fraction specific isotopic values for food groups obtained by averaging the species-specific (ancient) edible macronutrient isotopic values. Uncertainties of offsets are propagated into the eventual uncertainties for the edible macronutrient isotopic values by quadratic summing with the standard error of the mean isotopic values. Additionally, a conservative estimate for the uncertainty of the TR/Energy value has been obtained as $|\delta^{13}C_{fat} - \delta^{13}C_{carbohydrate}|/2$.

Food group	Food fraction	$\delta^{13}C$(‰)	±	$\delta^{15}N$(‰)	±	Notes
TR	Protein	-25.8	0.7	3.6	0.9	
TR	Energy	-27.7	3.3			average of fat and carbohydrate values
FA	Protein	-29.0	0.9	15.2	0.8	
FA	Energy	-36.0	1.1			
$MA_{Bothnia}$	Protein	-20.0	0.6	14.0	0.8	latitudes 63-66°N
$MA_{Bothnia}$	Energy	-27.0	0.8			latitudes 63-66°N
MA_{Baltic}	Protein	-15.9	0.9	14.0	0.8	latitudes 56-63°N
MA_{Baltic}	Energy	-22.9	1.0			latitudes 56-63°N
MA_{total}	Protein	-18.2	0.8	13.9	0.7	latitudes 56-66°N
MA_{total}	Energy	-25.2	0.9			latitudes 56-66°N

References

1. Etu-Sihvola H, Bocherens H, Drucker DG, Junno A, Mannermaa K, Oinonen M, et al. The dIANA database – Resource for isotopic paleodietary research in the Baltic Sea area. J Archaeol Sci Reports. 2019;24: 1003–1013. doi:10.1016/j.jasrep.2019.03.005
2. Syväranta J. Private communication. 2016.
3. Keeling CD. The Suess effect: 13Carbon-14Carbon interrelations. Environ Int. 1979;2: 229–300. doi:10.1016/0160-4120(79)90005-9
4. Bocherens H, Drucker D. Trophic level isotopic enrichment of carbon and nitrogen in bone collagen: case studies from recent and ancient terrestrial ecosystems. Int J Osteoarchaeol. 2003;13: 46–53. doi:10.1002/oa.662
5. Fraser RA, Bogaard A, Charles M, Styring AK, Wallace M, Jones G, et al. Assessing natural variation and the effects of charring, burial and pre-treatment on the stable carbon and nitrogen isotope values of archaeobotanical cereals and pulses. J Archaeol Sci. 2013;40: 4754–4766. doi:10.1016/J.JAS.2013.01.032
6. Bogaard A, Fraser R, Heaton THE, Wallace M, Vaiglova P, Charles M, et al. Crop manuring and intensive land management by Europe's first farmers. Proc Natl Acad Sci U S A. 2013;110: 12589–94. doi:10.1073/pnas.1305918110
7. Sjögren K-G. Modeling middle Neolithic funnel beaker diet on Falbygden, Sweden. J Archaeol Sci Reports. 2017;12: 295–306. doi:10.1016/J.JASREP.2017.01.044
8. Lightfoot E, Stevens RE. Stable isotope investigations of charred barley (Hordeum vulgare) and wheat (Triticum spelta) grains from Danebury Hillfort: implications for palaeodietary reconstructions. J Archaeol Sci. 2012;39: 656–662. doi:10.1016/J.JAS.2011.10.026
9. Bocherens H. Isotopic tracking of large carnivore palaeoecology in the mammoth steppe. Quaternary Science Reviews. 2015. pp. 42–71. doi:10.1016/j.quascirev.2015.03.018
10. Drucker DG, Hobson K., Münzel SC, Pike-Tay A. Intra-individual variation in stable carbon (δ13C) and nitrogen (δ15N) isotopes in mandibles of modern caribou of Qamanirjuaq (Rangifer tarandus groenlandicus) and Banks Island (Rangifer tarandus pearyi): Implications for tracing seasonal and temporal cha. Int J Osteoarchaeol. 2012;22: 494–504. doi:10.1002/oa.1220
11. Kosiba SB, Tykot RH, Carlsson D. Stable isotopes as indicators of change in the food procurement and food preference of Viking Age and Early Christian populations on Gotland (Sweden). J Anthropol Archaeol. 2007;26: 394–411. doi:10.1016/j.jaa.2007.02.001
12. Osmond CB, Valaane N, Haslam SM, Uotila P, Roksandic Z. Comparisons of d13C values in leaves of aquatic macrophytes from different habitats in Britain and Finland; some implications for photosynthetic processes in aquatic plants. Oecologia. 1981;50: 117–124. doi:10.1007/BF00378804
13. Lougheed BC, Filipsson HL, Snowball I. Large spatial variations in coastal 14C reservoir age – a case study from the Baltic Sea. Clim Past. 2013;9: 1015–1028. doi:10.5194/cp-9-1015-2013
14. Cordain L, Miller JB, Eaton SB, Mann N, Holt SHA, Speth JD. Plant-animal

subsistence ratios and macronutrient energy estimations in worldwide hunter-gatherer diets. Am J Clin Nutr. 2000;71: 682–692.
15. Wessman A, Alenius T, Holmqvist E, Mannermaa K, Perttola W, Sundell T, et al. Hidden and Remote: New Perspectives on the People in the Levänluhta Water Burial, Western Finland (c. ad 300–800). Eur J Archaeol. 2018; 1–24. doi:10.1017/eaa.2017.84
16. Nash SH, Bersamin A, Kristal AR, Hopkins SE, Church RS, Pasker RL, et al. Stable Nitrogen and Carbon Isotope Ratios Indicate Traditional and Market Food Intake in an Indigenous Circumpolar Population. J Nutr. 2012;142: 84–90. doi:10.3945/jn.111.147595
17. Salmi A-K, Äikäs T, Fjellström M, Spangen M. Animal offerings at the Sámi offering site of Unna Saiva – Changing religious practices and human–animal relationships. J Anthropol Archaeol. 2015;40: 10–22. doi:10.1016/J.JAA.2015.05.003
18. Linderholm A, Andersson K, Mörth C-M, Grundberg L, Hårding B, Lindén K. An early Christian cemetery at Björned in northern Sweden: Stable isotope analyses of skeletal material. Fornvännen. 2008.
19. Linderholm A, Jonson CH, Svensk O, Lidén K. Diet and status in Birka: stable isotopes and grave goods compared. Antiquity. 2008;82: 446–461. doi:10.1017/S0003598X00096939
20. Linderholm A, Kjellström A. Stable isotope analysis of a medieval skeletal sample indicative of systemic disease from Sigtuna Sweden. J Archaeol Sci. 2011;38: 925–933. doi:10.1016/j.jas.2010.11.022
21. Kjellström A, Storå J, Possnert G, Linderholm A. Dietary patterns and social structures in medieval Sigtuna, Sweden, as reflected in stable isotope values in human skeletal remains. J Archaeol Sci. 2009;36: 2689–2699. doi:10.1016/j.jas.2009.08.007
22. Fornander E. A shattered tomb of scattered people: The alvastra dolmen in light of stable isotopes. Curr S. 2011;19: 113–141. Available: https://www.scopus.com/record/display.uri?eid=2-s2.0-84862098552&origin=inward&txGid=3ebb407f8e72c72433ea7827502f7874
23. Bläuer A, Arppe L, Niemi M, Oinonen M, Liden K, Taavitsainen J-P, et al. Inferring prehistorical and historical feeding practices from δ15N and δ13C isotope analysis on Finnish archaeological domesticated ruminant bones and teeth. Fennoscandia Archaeol. 2017;XXXIII: 171–188.
24. Lahtinen M, Salmi A-K. Mixed Livelihood Society in Iin Hamina – a Case Study of Medieval Diet in the Northern Ostrobothnia, Finland. Environ Archaeol. 2018; 1–14. doi:10.1080/14614103.2018.1444695
25. Andersson K. Diet & Identitet. Analyser av kol-, kväve, och svalisotoper på individer från det kristna senvikingatida gravfältet i Björned, Torsåkers socken, Ångermanland. University of Stockholm. 2006.
26. Schyman J. Proveniensbestämning av vikingatida hornmaterial. En studie utifrån stabila isotoper. University of Stockholm. 2012.
27. Hinders J. Dödsrikets livshistorier - Benkemiska isotopanalyser på artikulerade och disartikulerade individer i Frälsegårdens gånggrift. University of Stockholm. 2011. Available: https://www.diva-

portal.org/smash/get/diva2:439797/FULLTEXT01.pdf
28. Molkentin J, Giesemann A. Follow-up of stable isotope analysis of organic versus conventional milk. Anal Bioanal Chem. 2010;398: 1493–1500. doi:10.1007/s00216-010-3995-y
29. Linderholm A. Migration in prehistory: DNA and stable isotype analyses of Swedish skeletal material. University of Stockholm. 2008.
30. Kohn MJ. Carbon isotope compositions of terrestrial C3 plants as indicators of (paleo)ecology and (paleo)climate. Proc Natl Acad Sci U S A. 2010;107: 19691–5. doi:10.1073/pnas.1004933107
31. Syväranta J, Jones RI. Changes in feeding niche widths of perch and roach following biomanipulation, revealed by stable isotope analysis. Freshw Biol. 2008;53: 425–434. doi:10.1111/j.1365-2427.2007.01905.x
32. Ravinet M, Syväranta J, Jones RI, Grey J. A trophic pathway from biogenic methane supports fish biomass in a temperate lake ecosystem. Oikos. 2010;119: 409–416. doi:10.1111/j.1600-0706.2009.17859.x
33. Eriksson G. Part-time farmers or hard-core sealers? Västerbjers studied by means of stable isotope analysis. J Antropol Archaeol. 2004;23: 135–162.
34. Eriksson G, Linderholm A, Fornander E, Kanstrup M, Schoultz P, Olofsson H, et al. Same island, different diet: Cultural evolution of food practice on Öland, Sweden, from the Mesolithic to the Roman Period. J Anthropol Archaeol. 2008;27: 520–543. doi:10.1016/J.JAA.2008.08.004
35. Barrett JH, Orton D, Johnstone C, Harland J, Van Neer W, Ervynck A, et al. Interpreting the expansion of sea fishing in medieval Europe using stable isotope analysis of archaeological cod bones. J Archaeol Sci. 2011;38: 1516–1524. doi:10.1016/J.JAS.2011.02.017
36. Orton DC, Makowiecki D, de Roo T, Johnstone C, Harland J, Jonsson L, et al. Stable Isotope Evidence for Late Medieval (14th–15th C) Origins of the Eastern Baltic Cod (Gadus morhua) Fishery. Thrush S, editor. PLoS One. 2011;6: e27568. doi:10.1371/journal.pone.0027568
37. Kiljunen M, Grey J, Sinisalo T, Harrod C, Immonen H, Jones RI. A revised model for lipid-normalizing δ13C values from aquatic organisms, with implications for isotope mixing models. J Appl Ecol. 2006;43: 1213–1222. doi:10.1111/j.1365-2664.2006.01224.x
38. Mänttäri V. Diets of grey seals (Halichoerus grypus) and Baltic ringed seals (Phoca hispida botnica) in the Bothnian Bay. University of Jyväskylä. 2011. Available: https://jyx.jyu.fi/bitstream/handle/123456789/36665/URN:NBN:fi:jyu-2011090911377.pdf?sequence=1
39. Antanaitis-Jacobs I, Richards M, Daugnora L, Jankauskas R, Ogrinc N. Diet in Early Lithuanian Prehistory and the New Stable Isotope Evidence. Archaeol Balt. 2009;12: 12–30.
40. Sinisalo T, Jones RI, Helle E, Valtonen ET. Changes in diets of individual Baltic ringed seals (Phoca hispida botnica) during their breeding season inferred from stable isotope analysis of multiple tissues. Mar Mammal Sci. 2008;24: 159–170. doi:10.1111/j.1748-7692.2007.00170.x
41. Fernandes R. A Simple(R) Model to Predict the Source of Dietary Carbon in Individual Consumers. Archaeometry. 2016;58. doi:10.1111/arcm.12193

42. Fernandes R, Millard AR, Brabec M, Nadeau M-J, Grootes P. Food Reconstruction Using Isotopic Transferred Signals (FRUITS): A Bayesian Model for Diet Reconstruction. PLoS One. 2014;9: e87436. doi:10.1371/JOURNAL.PONE.0087436
43. DeNiro MJ, Epstein S. Mechanism of carbon isotope fractionation associated with lipid synthesis. Science (80-). 1977;197: 261–3. doi:10.1126/SCIENCE.327543
44. Post DM, Layman CA, Arrington DA, Takimoto G, Quattrochi J, Montaña CG. Getting to the fat of the matter: models, methods and assumptions for dealing with lipids in stable isotope analyses. Oecologia. 2007;152: 179–189. doi:10.1007/s00442-006-0630-x
45. Vogel JC. Isotopic assessment of the dietary habits of ungulates. S Afr J Sci. 1978;74: 298–301. Available: https://inis.iaea.org/search/search.aspx?orig_q=RN:9411123
46. Schneider S, Auerswald K, Bellof G, Schnyder H. 13C discrimination between diet, faeces, milk and milk components. Isotopes Environ Health Stud. 2015;51: 33–45. doi:http://dx.doi.org/10.1080/10256016.2014.988151
47. Feng X. Long-term c i / c a response of trees in western North America to atmospheric CO 2 concentration derived from carbon isotope chronologies. Oecologia. 1998;117: 19–25. doi:10.1007/s004420050626
48. Fernandes R, Nadeau M-J, Grootes PM. Macronutrient-based model for dietary carbon routing in bone collagen and bioapatite. Archaeol Anthropol Sci. 2012;4: 291–301. doi:10.1007/s12520-012-0102-7
49. Fuller BT, Fuller JL, Harris DA, Hedges REM. Detection of breastfeeding and weaning in modern human infants with carbon and nitrogen stable isotope ratios. Am J Phys Anthropol. 2006;129: 279–293. doi:10.1002/ajpa.20249
50. Fuller BT, Molleson TI, Harris DA, Gilmour LT, Hedges REM. Isotopic Evidence for Breastfeeding and Possible Adult Dietary Differences from Late/Sub-Roman Britain. Am J Phys Anthropol. 2006;129: 45–54. doi:10.1002/ajpa.20244
