# Supplementary material for: Buried in water, burdened by nature—Resilience carried the Iron Age people through Fimbulvinter
Source: PLoS One. 2020 Apr 21;15(4):e0231787. doi: 10.1371/journal.pone.0231787 (PMC7173937; doi:10.1371/journal.pone.0231787)
Supplement: S6 Appendix — (PDF) [file pone.0231787.s006.pdf]

Supporting Information **S6 Appendix. Dietary modellings** for

Buried in water, burdened by nature – Resilience carried the Iron Age people through Fimbulvinter

Corresponding author: Markku Oinonen

Contributors: Markku Oinonen

S6 Appendix contains: Text, Table K-M

Text

The established isotopic baseline combined with food-specific macronutrient concentrations (Table K) allow for dietary modellings with FRUITS software(1). These modellings provide food group fractions of terrestrial (TR), freshwater (FA) and marine (MA) origin (Table L, M).

Table K. Macronutrient dry-mass and dry-mass carbon concentrations for the food groups and associated foods. TR = Terrestrial resources, FA = Freshwater animals, MA = Marine animals, FI = data from FINELI database (https://fineli.fi/fineli/en/index), US = data from National Nutrient Database, United States Department of Agriculture (http://ndb.nal.usda.gov/ndb/search). The following carbon contents of the macronutrients were assumed to calculate carbon mass fractions: Proteins → 52.4 %. Fats → 76.9 %. Carbohydrates → 44.4 %. Energy fraction has been calculated by summing Fats and Carbohydrates fractions.

Food group → ↓ Food fraction	Terrestrial resources (TR)	Standard Error of the Mean, TR	Freshwater animals (FA)	Standard Error of the Mean, FA	Marine animals (MA)	Standard Error of the Mean, MA
Proteins (wt%)	41.3	7.8	95.2	1.8	75.4	11.5
Fats (wt%)	18.5	4.3	4.8	1.8	24.6	11.5
Carbohydrates (wt%)	40.2	9.9	0.0	0.0	0.0	0.0
Energy(wt%)	58.7	10.8	4.8	1.8	24.6	11.5
Proteins (wtC%)	21.6	4.1	49.9	0.9	39.5	6.0
Fats (wtC%)	14.2	3.3	3.7	1.4	18.9	8.8

Food group → ↓ Food fraction	Terrestrial resources (TR)	Standard Error of the Mean, TR	Freshwater animals (FA)	Standard Error of the Mean, FA	Marine animals (MA)	Standard Error of the Mean, MA
Carbohydrates (wtC%)	17.8	4.4	0.0	0.0	0.0	0.0
Energy (wtC%)	32.1	5.9	3.7	1.4	18.9	8.8
N	30		5		8	
	Species, food		Species, food		Species, food	
	Barley, flour(FI)		Burbot, meat(FI)		Baltic herring, meat(FI)	
	Beaver, meat(US)		Perch, meat(FI)		Cod, meat(FI)	
	Blueberry(FI)		Pike, meat(FI)		Perch, meat(FI)	
	Buckwheat, flour(US)		Roach, meat(FI)		Pike, meat(FI)	
	Cattail, flour(US)		Zander, meat(FI)		Ringed seal, meat(US)	
	Cattle, cheese(FI)				Salmon, meat(FI)	
	Cattle, meat(FI)				Seal, blubber(2)	
	Cattle, milk(FI)				Whitefish, meat(FI)	
	Chicken, meat(FI)					
	Cloudberry(FI)					
	Dandelion, leaves(US)					
	Dandelion, roots(US)					
	Elk, meat(FI)					
	Goat, cheese(FI)					
	Goat, meat(US)					
	Goat, milk(FI)					
	Hare, meat(FI)					

Food group → ↓ Food fraction	Terrestrial resources (TR)	Standard Error of the Mean, TR	Freshwater animals (FA)	Standard Error of the Mean, FA	Marine animals (MA)	Standard Error of the Mean, MA
	Horse, meat(US)					
	Lamb, meat(FI)					
	Mushroom (bolete, brittlegill)(FI)					
	Oat, flour(FI)					
	Pea(FI)					
	Pig, meat(FI)					
	Pine bark, flour(3)					
	Raspberry(FI)					

Table L. Data from the FRUITS dietary modellings performed within this study. LL = Levänluhta, KM = Käldamäki, $\Theta_{XX,C}$ = relative contribution of the food group (XX = TR, FA, MA) towards the carbon isotopic signal, α_{XX} = relative contribution of the food group towards the diet, $\beta_{Protein/Energy}$ = relative contribution of the food fraction (Protein or Energy) towards the diet, σ's = standard deviations provided by the FRUITS dietary modelling. The Bothnian Bay (latitudes 63-66°N) was assumed as a source of carbon for all the samples. The following results were rejected from the dietary modelling and subsequent analysis based on quality criteria or radiocarbon dating. #4: radiocarbon age too young (139 ± 30 BP); #5: right femur; #7: high C/N value; #8,9: C-% not measured; #17: too small sample; #18: radiocarbon age too old (4124 ± 34 BP); #28: C-%, N-% not measured; , #32: risk of being duplicate; #33,34: children and thus potentially subject to breastfeeding effect.

Site	Sample #, k	$\Theta_{MA,C}$	σ	$\Theta_{FA,C}$	σ	α_{TR}	σ	α_{FA}	σ	α_{MA}	σ	$\beta_{Protein}$	σ	β_{Energy}	σ
LL	1	0.22	0.09	0.09	0.06	0.75	0.08	0.07	0.05	0.18	0.09	0.50	0.06	0.50	0.06
LL	2	0.17	0.09	0.13	0.07	0.78	0.07	0.09	0.06	0.13	0.08	0.48	0.08	0.52	0.08
LL	3	0.08	0.06	0.08	0.06	0.87	0.05	0.06	0.04	0.07	0.05	0.46	0.06	0.54	0.06
~~LL~~	~~4~~	~~0.18~~	~~0.08~~	~~0.04~~	~~0.04~~	~~0.83~~	~~0.07~~	~~0.03~~	~~0.03~~	~~0.14~~	~~0.07~~	~~0.45~~	~~0.07~~	~~0.55~~	~~0.07~~
~~LL~~	~~5~~	~~0.06~~	~~0.05~~	~~0.06~~	~~0.05~~	~~0.91~~	~~0.04~~	~~0.04~~	~~0.03~~	~~0.05~~	~~0.04~~	~~0.46~~	~~0.06~~	~~0.54~~	~~0.06~~
LL	6	0.13	0.07	0.04	0.03	0.87	0.06	0.03	0.02	0.10	0.06	0.45	0.06	0.55	0.06
~~LL~~	~~7~~	~~0.06~~	~~0.05~~	~~0.08~~	~~0.05~~	~~0.90~~	~~0.05~~	~~0.05~~	~~0.04~~	~~0.05~~	~~0.04~~	~~0.45~~	~~0.06~~	~~0.55~~	~~0.06~~
~~LL~~	~~8~~	~~0.19~~	~~0.09~~	~~0.12~~	~~0.07~~	~~0.76~~	~~0.07~~	~~0.09~~	~~0.05~~	~~0.15~~	~~0.08~~	~~0.49~~	~~0.06~~	~~0.51~~	~~0.06~~
~~LL~~	~~9~~	~~0.17~~	~~0.09~~	~~0.24~~	~~0.09~~	~~0.68~~	~~0.08~~	~~0.18~~	~~0.08~~	~~0.14~~	~~0.08~~	~~0.54~~	~~0.07~~	~~0.46~~	~~0.07~~
LL	10	0.17	0.09	0.13	0.07	0.78	0.07	0.09	0.06	0.13	0.08	0.48	0.08	0.52	0.08
LL	11	0.10	0.07	0.10	0.06	0.85	0.06	0.07	0.05	0.08	0.06	0.48	0.07	0.52	0.07
LL	12	0.10	0.06	0.04	0.03	0.90	0.05	0.02	0.02	0.08	0.05	0.44	0.06	0.56	0.06
LL	13	0.25	0.10	0.03	0.03	0.78	0.08	0.02	0.02	0.20	0.08	0.46	0.06	0.54	0.06
LL	14	0.75	0.12	0.02	0.02	0.28	0.14	0.02	0.02	0.70	0.14	0.66	0.09	0.34	0.09
LL	15	0.64	0.10	0.03	0.03	0.33	0.10	0.03	0.03	0.64	0.10	0.60	0.07	0.40	0.07
LL	16	0.06	0.05	0.07	0.05	0.91	0.04	0.05	0.04	0.05	0.04	0.45	0.06	0.55	0.06
~~LL~~	~~17~~	~~NA~~	~~NA~~	~~NA~~	~~NA~~	~~NA~~	~~NA~~	~~NA~~	~~NA~~	~~NA~~	~~NA~~	~~NA~~	~~NA~~	~~NA~~	~~NA~~
~~LL~~	~~18~~	~~0.19~~	~~0.09~~	~~0.17~~	~~0.08~~	~~0.72~~	~~0.07~~	~~0.13~~	~~0.07~~	~~0.15~~	~~0.08~~	~~0.52~~	~~0.06~~	~~0.48~~	~~0.06~~
LL	19	0.11	0.07	0.09	0.06	0.85	0.06	0.07	0.05	0.09	0.06	0.48	0.06	0.52	0.06
LL	20	0.15	0.08	0.07	0.05	0.83	0.06	0.05	0.04	0.12	0.07	0.47	0.06	0.53	0.06
LL	21	0.36	0.10	0.07	0.05	0.65	0.09	0.05	0.04	0.30	0.10	0.50	0.07	0.50	0.07

Site	Sample #, k	$\Theta_{MA,C}$	σ	$\Theta_{FA,C}$	σ	α_{TR}	σ	α_{FA}	σ	α_{MA}	σ	$\beta_{Protein}$	σ	β_{Energy}	σ
LL	22	0.08	0.06	0.09	0.06	0.88	0.05	0.06	0.04	0.06	0.05	0.45	0.07	0.55	0.07
LL	23	0.07	0.06	0.10	0.06	0.87	0.05	0.07	0.05	0.06	0.05	0.46	0.06	0.54	0.06
LL	24	0.06	0.05	0.06	0.05	0.91	0.04	0.04	0.03	0.05	0.04	0.45	0.06	0.55	0.06
LL	25	0.17	0.09	0.13	0.08	0.76	0.07	0.10	0.06	0.14	0.07	0.50	0.06	0.50	0.06
LL	26	0.08	0.06	0.08	0.05	0.88	0.05	0.05	0.04	0.06	0.05	0.46	0.06	0.54	0.06
LL	27	0.38	0.11	0.04	0.03	0.66	0.10	0.03	0.02	0.32	0.10	0.51	0.07	0.49	0.07
~~LL~~	~~28~~	~~0.65~~	~~0.09~~	~~0.04~~	~~0.04~~	~~0.38~~	~~0.11~~	~~0.04~~	~~0.03~~	~~0.58~~	~~0.11~~	~~0.59~~	~~0.07~~	~~0.41~~	~~0.07~~
LL	29	0.10	0.07	0.07	0.05	0.87	0.05	0.05	0.04	0.08	0.06	0.47	0.06	0.53	0.06
LL	30	0.08	0.06	0.08	0.05	0.88	0.05	0.06	0.04	0.06	0.05	0.45	0.06	0.55	0.06
LL	31	0.12	0.07	0.06	0.05	0.86	0.06	0.04	0.03	0.10	0.06	0.46	0.07	0.54	0.07
~~LL~~	~~32~~	~~0.07~~	~~0.05~~	~~0.09~~	~~0.06~~	~~0.89~~	~~0.05~~	~~0.06~~	~~0.04~~	~~0.05~~	~~0.04~~	~~0.46~~	~~0.06~~	~~0.54~~	~~0.06~~
LL	33	0.07	0.06	0.10	0.06	0.87	0.05	0.07	0.05	0.06	0.05	0.46	0.06	0.54	0.06
LL	34	0.12	0.07	0.10	0.06	0.83	0.06	0.07	0.05	0.10	0.06	0.46	0.07	0.54	0.07
LL	35	0.10	0.07	0.07	0.05	0.87	0.05	0.05	0.04	0.08	0.05	0.48	0.06	0.52	0.06
LL	36	0.10	0.07	0.11	0.07	0.84	0.06	0.08	0.05	0.08	0.06	0.47	0.06	0.53	0.06
LL	37	0.08	0.06	0.08	0.06	0.87	0.05	0.06	0.04	0.07	0.05	0.46	0.06	0.54	0.06
LL	38	0.16	0.08	0.03	0.03	0.85	0.07	0.02	0.02	0.13	0.07	0.41	0.06	0.59	0.06
LL	39	0.16	0.08	0.08	0.06	0.81	0.07	0.06	0.04	0.13	0.07	0.48	0.06	0.52	0.06
KM	40	0.11	0.07	0.08	0.06	0.86	0.06	0.06	0.04	0.09	0.06	0.48	0.07	0.52	0.07
KM	41	0.28	0.09	0.06	0.05	0.73	0.08	0.04	0.04	0.22	0.09	0.48	0.07	0.52	0.07
KM	42	0.14	0.08	0.08	0.06	0.83	0.06	0.06	0.04	0.11	0.07	0.48	0.06	0.52	0.06
KM	43	0.22	0.09	0.06	0.05	0.78	0.07	0.05	0.04	0.18	0.08	0.50	0.06	0.50	0.06

Table M. Alternative data from the FRUITS dietary modellings performed within this study. Compared to data of Table L and discussed in the text, the data in Table M assumes the whole area from Öland to the end of the Bothnian Bay (latitudes 56-66°N, *total* scenario) as the source of marine carbon for the samples.

Site	Sample #, k	$\Theta_{MA,C}$	σ	$\Theta_{FA,C}$	σ	α_{TR}	σ	α_{FA}	σ	α_{MA}	σ	$\beta_{Protein}$	σ	β_{Energy}	σ
LL	1	0.20	0.08	0.11	0.06	0.76	0.07	0.08	0.05	0.16	0.07	0.49	0.06	0.51	0.06
LL	2	0.14	0.08	0.15	0.08	0.77	0.07	0.11	0.06	0.12	0.07	0.49	0.07	0.51	0.07
LL	3	0.07	0.05	0.09	0.06	0.88	0.05	0.06	0.04	0.06	0.04	0.47	0.06	0.54	0.06
~~LL~~	~~4~~	~~0.19~~	~~0.08~~	~~0.04~~	~~0.04~~	~~0.82~~	~~0.07~~	~~0.03~~	~~0.02~~	~~0.15~~	~~0.07~~	~~0.45~~	~~0.07~~	~~0.55~~	~~0.07~~
~~LL~~	~~5~~	~~0.06~~	~~0.05~~	~~0.06~~	~~0.05~~	~~0.91~~	~~0.04~~	~~0.04~~	~~0.03~~	~~0.05~~	~~0.04~~	~~0.46~~	~~0.06~~	~~0.54~~	~~0.06~~
LL	6	0.14	0.07	0.04	0.03	0.87	0.06	0.03	0.02	0.11	0.06	0.45	0.07	0.55	0.07
~~LL~~	~~7~~	~~0.06~~	~~0.05~~	~~0.07~~	~~0.05~~	~~0.90~~	~~0.04~~	~~0.05~~	~~0.04~~	~~0.05~~	~~0.04~~	~~0.45~~	~~0.06~~	~~0.55~~	~~0.06~~
~~LL~~	~~8~~	~~0.18~~	~~0.08~~	~~0.13~~	~~0.07~~	~~0.76~~	~~0.07~~	~~0.10~~	~~0.06~~	~~0.14~~	~~0.07~~	~~0.49~~	~~0.07~~	~~0.51~~	~~0.07~~
~~LL~~	~~9~~	~~0.15~~	~~0.08~~	~~0.26~~	~~0.09~~	~~0.69~~	~~0.08~~	~~0.19~~	~~0.08~~	~~0.12~~	~~0.07~~	~~0.53~~	~~0.07~~	~~0.47~~	~~0.07~~
LL	10	0.14	0.08	0.15	0.08	0.77	0.07	0.11	0.06	0.12	0.07	0.49	0.07	0.51	0.07
LL	11	0.09	0.06	0.11	0.06	0.85	0.05	0.08	0.05	0.07	0.05	0.47	0.06	0.53	0.06
LL	12	0.11	0.07	0.03	0.03	0.89	0.06	0.02	0.02	0.09	0.06	0.44	0.06	0.56	0.06
LL	13	0.29	0.09	0.03	0.03	0.74	0.08	0.02	0.02	0.23	0.09	0.48	0.06	0.52	0.06
LL	14	0.66	0.07	0.03	0.03	0.39	0.09	0.03	0.02	0.59	0.09	0.60	0.07	0.40	0.07
LL	15	0.56	0.08	0.05	0.04	0.47	0.09	0.04	0.03	0.49	0.09	0.56	0.07	0.44	0.07
LL	16	0.06	0.04	0.07	0.05	0.91	0.04	0.05	0.04	0.04	0.04	0.45	0.06	0.55	0.06
~~LL~~	~~17~~	~~NA~~	~~NA~~	~~NA~~	~~NA~~	~~NA~~	~~NA~~	~~NA~~	~~NA~~	~~NA~~	~~NA~~	~~NA~~	~~NA~~	~~NA~~	~~NA~~
~~LL~~	~~18~~	~~0.15~~	~~0.08~~	~~0.19~~	~~0.08~~	~~0.74~~	~~0.07~~	~~0.14~~	~~0.07~~	~~0.13~~	~~0.07~~	~~0.51~~	~~0.07~~	~~0.49~~	~~0.07~~
LL	19	0.10	0.06	0.10	0.06	0.85	0.05	0.07	0.04	0.08	0.05	0.47	0.07	0.53	0.07
LL	20	0.14	0.07	0.08	0.05	0.84	0.06	0.05	0.04	0.11	0.06	0.47	0.06	0.53	0.06
LL	21	0.34	0.08	0.08	0.05	0.66	0.08	0.06	0.04	0.28	0.08	0.51	0.05	0.49	0.05
LL	22	0.07	0.05	0.09	0.06	0.88	0.05	0.06	0.04	0.06	0.04	0.46	0.06	0.54	0.06
LL	23	0.07	0.05	0.10	0.06	0.88	0.05	0.07	0.05	0.05	0.04	0.46	0.06	0.54	0.06
LL	24	0.06	0.05	0.06	0.05	0.91	0.04	0.04	0.03	0.05	0.04	0.45	0.06	0.55	0.06
LL	25	0.16	0.08	0.13	0.07	0.78	0.07	0.09	0.06	0.12	0.07	0.48	0.07	0.52	0.07

Site	Sample #, k	$\Theta_{MA,C}$	σ	$\Theta_{FA,C}$	σ	α_{TR}	σ	α_{FA}	σ	α_{MA}	σ	$\beta_{Protein}$	σ	β_{Energy}	σ
LL	26	0.08	0.05	0.08	0.05	0.89	0.05	0.05	0.04	0.06	0.04	0.46	0.06	0.54	0.06
LL	27	0.40	0.08	0.04	0.03	0.64	0.08	0.03	0.02	0.33	0.08	0.52	0.06	0.48	0.06
~~LL~~	~~28~~	~~0.57~~	~~0.07~~	~~0.07~~	~~0.05~~	~~0.43~~	~~0.09~~	~~0.06~~	~~0.04~~	~~0.51~~	~~0.08~~	~~0.58~~	~~0.06~~	~~0.42~~	~~0.06~~
LL	29	0.10	0.06	0.07	0.05	0.88	0.05	0.05	0.04	0.08	0.05	0.47	0.06	0.53	0.06
LL	30	0.07	0.05	0.09	0.06	0.88	0.05	0.06	0.04	0.06	0.04	0.46	0.06	0.54	0.06
LL	31	0.12	0.07	0.06	0.05	0.86	0.06	0.04	0.03	0.10	0.06	0.47	0.06	0.53	0.06
~~LL~~	~~32~~	~~0.06~~	~~0.05~~	~~0.09~~	~~0.06~~	~~0.89~~	~~0.05~~	~~0.06~~	~~0.04~~	~~0.05~~	~~0.04~~	~~0.46~~	~~0.07~~	~~0.54~~	~~0.07~~
LL	33	0.07	0.05	0.10	0.06	0.88	0.05	0.07	0.05	0.05	0.04	0.46	0.06	0.54	0.06
LL	34	0.11	0.07	0.10	0.06	0.84	0.06	0.07	0.05	0.09	0.06	0.48	0.06	0.52	0.06
LL	35	0.10	0.06	0.06	0.05	0.88	0.05	0.04	0.03	0.08	0.05	0.47	0.06	0.53	0.06
LL	36	0.08	0.06	0.12	0.07	0.84	0.06	0.09	0.05	0.07	0.05	0.48	0.07	0.52	0.07
LL	37	0.07	0.05	0.09	0.06	0.88	0.05	0.06	0.04	0.06	0.04	0.47	0.06	0.54	0.06
LL	38	0.20	0.09	0.03	0.03	0.82	0.07	0.02	0.02	0.16	0.08	0.44	0.07	0.56	0.07
LL	39	0.16	0.08	0.09	0.06	0.81	0.06	0.06	0.04	0.13	0.07	0.49	0.06	0.51	0.06
KM	40	0.10	0.06	0.08	0.06	0.87	0.05	0.06	0.04	0.08	0.05	0.47	0.06	0.53	0.06
KM	41	0.27	0.09	0.07	0.05	0.73	0.07	0.05	0.04	0.22	0.08	0.49	0.06	0.51	0.06
KM	42	0.13	0.07	0.08	0.05	0.83	0.06	0.06	0.04	0.11	0.06	0.47	0.06	0.53	0.06
KM	43	0.20	0.08	0.07	0.05	0.78	0.06	0.05	0.04	0.17	0.07	0.48	0.06	0.52	0.06

References

1. R. Fernandes, A. R. Millard, M. Brabec, M.-J. Nadeau, P. Grootes, Food Reconstruction Using Isotopic Transferred Signals (FRUITS): A Bayesian Model for Diet Reconstruction. *PLoS One* **9**, e87436 (2014).
2. Nutritional Information, Diet Info and Calories in Seal Blubber (June 30, 2017).
3. A.-M. Rautio, G. Norstedt, L. Östlund, Nutritional Content of Scots Pine Inner Bark in Northern Fennoscandia. *Econ. Bot.* **67**, 363–377 (2013).
