# Supplementary material for: Buried in water, burdened by nature—Resilience carried the Iron Age people through Fimbulvinter
Source: PLoS One. 2020 Apr 21;15(4):e0231787. doi: 10.1371/journal.pone.0231787 (PMC7173937; doi:10.1371/journal.pone.0231787)
Supplement: S7 Appendix — (PDF) [file pone.0231787.s007.pdf]

Supporting Information **S7 Appendix. Timeline through radiocarbon dates** for

Buried in water, burdened by nature – Resilience carried the Iron Age people through Fimbulvinter

Corresponding author: Markku Oinonen

Contributors: Markku Oinonen, Anna Wessman

S7 Appendix contains: Text, Figure K-M

Text

Small use of freshwater food causes a limited systematic freshwater reservoir effect (FRE) on the radiocarbon datings of less than 14 ^{14}C years (mean = 8 ± 3 ^{14}C years) within the *Bothnia* scenario (*total* scenario: maximum = 21 ^{14}C years, mean = 9 ± 4 ^{14}C years) (Table 3 in the manuscript). Since the marine dietary influence and the maximal marine reservoir effect are larger, so is the effect. Particularly, the largest MRE corrections are deduced for LL3 for which they are 57 ^{14}C years (*Bothnia* scenario, mean = 13 ± 12 ^{14}C years) and 118 ^{14}C years (*total* scenario, mean = 30 ± 26 ^{14}C years) (Table 3 in the manuscript). These systematic shifts move the corrected ages correspondingly younger (Table 3 in the manuscript). Bayesian chronological models (examples for *Bothnia* given in Figs K, L) based on RE-corrected radiocarbon dates yield to estimates of the start and the end boundaries of the Levänluhta burial usage as well as summed calendar-year probability distributions through kernel density estimations (KDE) (Fig M). The quantitative estimates for these are given in Table 4 in the manuscript.

The larger marine reservoir effect correction applied shifts the start boundary for the *Baltic* and *total* scenarios significantly younger compared to the *Bothnia* scenario, but do not affect the end boundary. This trend is probably strengthened by larger individual dating uncertainties with the *Baltic* and *total* scenarios as they provide the Bayesian models more space to interpret dates to belong to the same grouping (see discussions at https://groups.google.com/forum/#!topic/oxcal/P1y7iUWn6Mk). So, based on a) the vicinity of the Levänluhta site to the Kvarken and Bothnian Bay and b) potential systematic tendency of the model to group the dates closer to each other with larger uncertainties, the *Bothnia* scenario is considered providing the most reliable quantitative estimates for the burial time range.

Recent archaeological investigations have led an estimation for the Levänluhta burial time range of AD 300 - 800[1,2]. Our *Bothnia* scenario yields to median values of AD 405 and AD 750 for boundary start and end, respectively, thus agreeing with the archaeological estimates (Fig Ma, Table 4 in the manuscript). The summed calendar-year probability distribution (scpd) through kernel density estimate (KDE) analyses provide essentially the similar results (Fig Mb). The probability rises at ca. AD 400, falls down at ca. AD 500, rises to its maximum at ca. AD 650 and declines towards ca. AD 800. It should be noted that boundary starts for the *Baltic* and *total* scenarios take place (mean)

at around AD 485-490 and, compared to KDE *scpd*, these are clearly too late. This can be considered as further supporting the selection of Bothnian Bay as the major source of the marine dietary input, as then the boundary and the KDE analyses agree.

```
Options()
 {
 Resolution=5;
 };
 Plot()
 {
 Outlier_Model("General",T(5),U(0,4),"t");
 Sequence("LL Bothnia_ch")
 {
  Boundary("LL Bothnia_ch start");
  Phase("LL Bothnia_ch")
  {
   R_Date("Hela-2128 Bothnia",1312,33){Outlier(0.05);};
   R_Date("Hela-2243 Bothnia",1447,33){Outlier(0.05);};
   R_Date("Hela-2262 Bothnia",1407,33){Outlier(0.05);};
   R_Date("Hela-3276 Bothnia",1406,37){Outlier(0.05);};
   R_Date("Hela-3280 Bothnia",1375,31){Outlier(0.05);};
   R_Date("Hela-3288 Bothnia_ch",1450,29){Outlier(0.05);};
   R_Date("Hela-3289 Bothnia_ch",1419,30){Outlier(0.05);};
   R_Date("Hela-2244 Bothnia",1660,31){Outlier(0.05);};
   R_Date("Hela-2263 Bothnia",1614,32){Outlier(0.05);};
   R_Date("Hela-3271 Bothnia",1527,32){Outlier(0.05);};
   R_Date("Hela-3274&ETH-57297 Bothnia",1580,66){Outlier(0.05);};
   R_Date("Hela-3275 Bothnia",1214,32){Outlier(0.05);};
   R_Date("Hela-3277&ETH-57298 Bothnia",1272,32){Outlier(0.05);};
   R_Date("Hela-3278 Bothnia",1638,31){Outlier(0.05);};
   R_Date("Hela-3279 Bothnia",1470,29){Outlier(0.05);};
   R_Date("ETH-55271 Bothnia",1400,29){Outlier(0.05);};
   R_Date("Hela-3284&ETH-57299 Bothnia",1507,30){Outlier(0.05);};
   R_Date("Hela-3285 Bothnia",1391,30){Outlier(0.05);};
   R_Date("Hela-3286 Bothnia",1515,29){Outlier(0.05);};
   R_Date("Hela-3290 Bothnia",1633,29){Outlier(0.05);};
   R_Date("Hela-3291 Bothnia",1294,29){Outlier(0.05);};
   R_Date("Hela-3292 Bothnia",1641,29){Outlier(0.05);};
   R_Date("Hela-3294 Bothnia",1380,30){Outlier(0.05);};
   R_Date("Hela-2251 Bothnia",1362,31){Outlier(0.05);};
   R_Date("Hela-2264 Bothnia",1536,31){Outlier(0.05);};
   R_Date("Hela-3268 Bothnia",1401,33){Outlier(0.05);};
   R_Date("Hela-3282 Bothnia",1658,34){Outlier(0.05);};
   R_Date("Hela-3293 Bothnia",1288,29){Outlier(0.05);};
   R_Date("Hela-3269 Bothnia",1573,46){Outlier(0.05);};
   R_Date("Hela-3270 Bothnia",1576,43){Outlier(0.05);};
   Sum("LL Bothnia_ch");
   Interval("Duration of LL Bothnia_ch");
  };
  Boundary("LL Bothnia_ch end");
  };
  N("Own age",18,5);
  Shift("Start","LL Bothnia_ch start","Own age");
  Shift("End","LL Bothnia_ch end","Own age");
 };
```

Fig K. An example for a Bayesian chronological model (*Bothnia* scenario with children) to obtain start and end boundaries. This particular model assumes the individuals with numbers #33 (Hela-3288) and #34 (Hela-3289) as being children with corresponding isotopic offsets.

```
Plot()
 {
 KDE_Model("LL Bothnia_ch")
 {
   R_Date("Hela-2128 Bothnia",1312,33);
   R_Date("Hela-2243 Bothnia",1447,33);
   R_Date("Hela-2262 Bothnia",1407,33);
   R_Date("Hela-3276 Bothnia",1406,37);
   R_Date("Hela-3280 Bothnia",1375,31);
   R_Date("Hela-3288 Bothnia",1450,29);
   R_Date("Hela-3289 Bothnia",1419,30);
   R_Date("Hela-2244 Bothnia",1660,31);
   R_Date("Hela-2263 Bothnia",1614,32);
   R_Date("Hela-3271 Bothnia",1527,32);
   R_Date("Hela-3274&ETH-57297 Bothnia",1580,66);
   R_Date("Hela-3275 Bothnia",1214,32);
   R_Date("Hela-3277&ETH-57298 Bothnia",1272,32);
   R_Date("Hela-3278 Bothnia",1638,31);
   R_Date("Hela-3279 Bothnia",1470,29);
   R_Date("ETH-55271 Bothnia",1400,29);
   R_Date("Hela-3284&ETH-57299 Bothnia",1507,30);
   R_Date("Hela-3285 Bothnia",1391,30);
   R_Date("Hela-3286 Bothnia",1515,29);
   R_Date("Hela-3290 Bothnia",1633,29);
   R_Date("Hela-3291 Bothnia",1294,29);
   R_Date("Hela-3292 Bothnia",1641,29);
   R_Date("Hela-3294 Bothnia",1380,30);
   R_Date("Hela-2251 Bothnia",1362,31);
   R_Date("Hela-2264 Bothnia",1536,31);
   R_Date("Hela-3268 Bothnia",1401,33);
   R_Date("Hela-3282 Bothnia",1658,34);
   R_Date("Hela-3293 Bothnia",1288,29);
   R_Date("Hela-3269 Bothnia",1573,46);
   R_Date("Hela-3270 Bothnia",1576,43);
 };
};
```

Fig L. An example for a Bayesian KDE model (*Bothnia* scenario with children) to obtain summed calendar-year probability distribution of Figure 3. This particular model assumes the individuals with numbers #33 (Hela-3288) and #34 (Hela-3289) as being children with corresponding isotopic offsets.

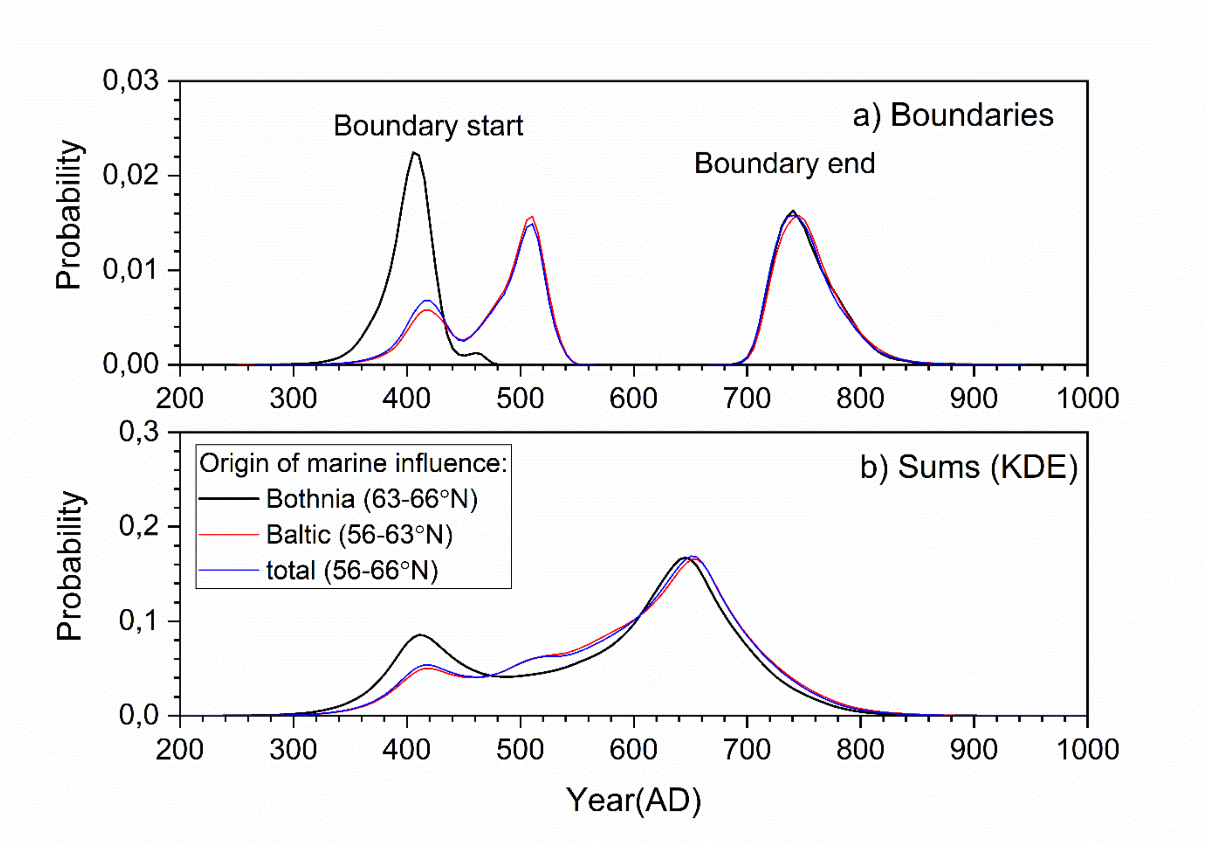

Fig M. Results of the Bayesian chronological modelling assuming different scenarios for the origin of marine diet (*Bothnia, Baltic, total*). a) Start and end boundaries of the Levänluhta burial usage. b) Summed calendar-year probability distributions through kernel density estimations (KDE) for the Levänluhta burial.

References

1. Wessman A. Levänluhta – a place of punishment, sacrifice or just a common cemetery? Fennoscandia Archaeol. 2009;XXVI: 47–71.
2. Wessman A, Alenius T, Holmqvist E, Mannermaa K, Perttola W, Sundell T, et al. Hidden and Remote: New Perspectives on the People in the Levänluhta Water Burial, Western Finland (c. ad 300–800). Eur J Archaeol. 2018; 1–24. doi:10.1017/eaa.2017.84
