# Supplementary material for: Buried in water, burdened by nature—Resilience carried the Iron Age people through Fimbulvinter
Source: PLoS One. 2020 Apr 21;15(4):e0231787. doi: 10.1371/journal.pone.0231787 (PMC7173937; doi:10.1371/journal.pone.0231787)
Supplement: S8 Appendix — (PDF) [file pone.0231787.s008.pdf]

Supporting Information **S8 Appendix. Time series data** for

Buried in water, burdened by nature – Resilience carried the Iron Age people through Fimbulvinter

Corresponding author: Markku Oinonen

Contributors: Markku Oinonen

S8 Appendix contains: Text, Figure N, Table N

Text

Time series data for the Figs 5 and 6 in the manuscript and Fig N in S8 are provided in Table N including moving averages of the number of dates, the isotopic ratios, their standard deviations and the modelled food fractions. The time dependence of the number of buried individuals (Fig Na) show essentially the same behavior as the KDE (Fig 4 in the manuscript) and *scpd* (Fig 7 in the manuscript) plots the essential feature being that the 50-year moving sum peaks at around AD 650. Additionally, the missing LL2 and LL3 subgroups seem to induce a slight post-anomalous decrease in the number of dates around AD 550 – 600, but low number of dates (N) highlighted by the large statistical uncertainties does not allow for definite conclusions.

Standard deviations of the isotopic ratios for the full set of Levänluhta individuals (Table D in S4 Appendix) is from larger (up to 7 times) than of the reference populations around the Baltic Sea. Scattering of the isotopic values along with the time is visualized through moving standard deviations (Fig Nb). If assuming the marine carbon originating from the Bothnian Bay, the scattering of isotopic values have been the largest during pre-anomalic period thus indicating broad spectrum of livelihoods already before the climatic anomaly. Particularly, this is due to strong marine influence of the LL3 subgroup. This is coherent with the summed calendar-year probability distributions (*scpd*; Fig 7 in the manuscript): *scpd*s for every cluster are present before the anomaly. The standard deviation collapses during the climatic anomaly since strongly marine individuals (LL3) do not fall within the 50-year time window used when estimating the moving standard deviations. This reflects the lack of such individuals dated to the temporal vicinity of the climatic anomaly thus supporting our interpretation on negative effects of the anomaly on seal hunting among the people buried in Levänluhta. However, non-negligible marine dietary fraction remains within the population throughout the era through LL1 subgroup. This does not show up within the standard deviation visualized by Fig Nb as it deals only with scattering between the individuals.

If assuming marine carbon sources from the majority of the Baltic basin (*Baltic* scenario), the isotopic baseline and MRE_{max} are affected and so is slightly the timeline. The scattering is similarly large before the anomaly but the largest scattering extends to be contemporaneous with the anomalic times. Within this scenario, the appearance of strongly marine individuals (LL3) possessing an isotopic signature of the Baltic proper around the anomalic times could be coherent with the emerging broader cultural contacts seen later during the Merovingian period. However, the above *Bothnia* scenario is considered as being more likely due to close proximity of the Levänluhta site to marine resources of Bothnian Bay / Kvarken.

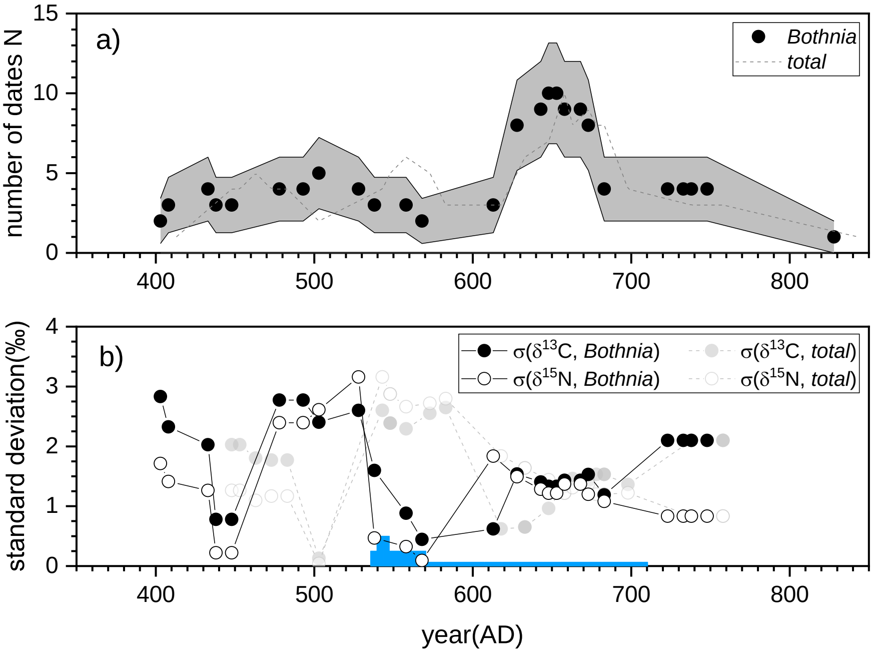

Fig N. Visualization of the trends of a) moving sum of number of dates (N) and b) moving average of standard deviations (σ) of the carbon and nitrogen isotopic ratios as a function of time, obtained by using a 50-year time window. The black lines/symbols correspond to the *Bothnia* scenario (63-66°N) for the source of marine carbon. The uncertainties of N (grey pattern in a) are obtained as √N. As a sensitivity analysis, values are given (grey dotted lines/symbols) also by assuming the *total* scenario (56-66°N) for the source of marine carbon. Chronologically, the symbols correspond to the mean values of the calendar-year probability distributions of individual RE-corrected radiocarbon dates with taking into account 18 ± 5 yr bone own age(1). The blue bars illustrate schematically the influence of the LALIA triggered by the climatic anomaly of AD 536 - 550.

Table N. Calibrations of RE-corrected radiocarbon dates in chronological order and corresponding time series data for the number of burials N, its uncertainty √N, the isotopic ratios $\delta^{13}C$ and $\delta^{15}N$, their standard deviations σ and the modelled food fractions α_{XX} (XX = TR, FA, MA). LL = Levänluhta, KM = Käldamäki, HPD = highest posterior density, early = start of the HPD region, late = end of the HPD region, σ = standard deviation of calendar-year probability distribution provided by the calibration, m.sum = moving sum, m.a. = moving average. The Bothnian Bay scenario as the origin of carbon was assumed for all the samples. All the ages are given as calendar years (calAD). Moving averages have been deduced with 50-year window, 25 years both sides of the mean value of the calendar year probability distribution. All values corresponding to the mean values falling within this time window have been averaged to obtain the moving average. *Mean values include the cortical bone own age correction of 18 ± 5 years(1) and thus the values reflect the moment of death / burial of an individual.

Site	Sample # k	68%HPD start	68%HPD end	95%HPD start	95%HPD end	mean*	σ	N m.sum	√N	$\delta^{13}C$ m.a.	σ m.a.	$\delta^{15}N$ m.a.	σ m.a.	α_{TR} m.a.	α_{FA} m.a.	α_{MA} m.a.
LL	3	345	420	255	530	403	45	2.0	1.4	-19.7	2.8	12.2	1.7	0.76	0.04	0.19
LL	27	345	420	255	535	408	50	3.0	1.7	-20.4	2.3	11.8	1.4	0.80	0.05	0.15
LL	37	350	430	335	535	433	50	4.0	2.0	-20.6	2.0	11.5	1.3	0.82	0.05	0.13
LL	23	355	530	335	535	438	50	3.0	1.7	-21.5	0.8	10.9	0.2	0.87	0.06	0.07
LL	35	380	530	340	535	448	50	3.0	1.7	-21.5	0.8	10.9	0.2	0.87	0.06	0.07
LL	11	395	535	380	540	478	50	4.0	2.0	-19.0	2.8	13.5	2.4	0.58	0.05	0.37
LL	19	410	550	335	615	493	70	4.0	2.0	-19.0	2.8	13.5	2.4	0.58	0.05	0.37
LL	14	425	540	390	585	503	50	5.0	2.2	-19.0	2.4	12.8	2.6	0.64	0.04	0.32
LL	15	425	540	395	575	503	50	5.0	2.2	-19.0	2.4	12.8	2.6	0.64	0.04	0.32
LL	12	430	570	425	595	528	50	4.0	2.0	-18.6	2.6	12.9	3.2	0.60	0.03	0.36
LL	16	430	585	425	605	538	50	3.0	1.7	-20.4	1.6	10.4	0.5	0.89	0.04	0.07
LL	31	470	600	425	615	558	50	3.0	1.7	-21.1	0.9	10.7	0.3	0.88	0.05	0.07
LL	29	535	605	430	635	568	50	2.0	1.4	-20.7	0.4	10.9	0.1	0.87	0.05	0.09
LL	24	565	625	545	645	613	30	3.0	1.7	-21.4	0.6	12.2	1.8	0.85	0.07	0.08
LL	2	590	645	555	655	628	25	8.0	2.8	-20.4	1.5	12.6	1.5	0.81	0.06	0.13
LL	33	590	645	560	655	628	25	8.0	2.8	-20.4	1.5	12.6	1.5	0.81	0.06	0.13
LL	34	610	655	580	665	643	21	9.0	3.0	-20.4	1.4	12.6	1.3	0.81	0.06	0.13
LL	10	615	660	580	670	648	21	10.0	3.2	-20.4	1.3	12.6	1.2	0.80	0.07	0.13
LL	21	610	660	575	675	648	25	10.0	3.2	-20.4	1.3	12.6	1.2	0.80	0.07	0.13
LL	13	615	660	590	675	653	21	10.0	3.2	-20.4	1.3	12.6	1.2	0.80	0.07	0.13
LL	26	620	660	595	670	653	21	10.0	3.2	-20.4	1.3	12.6	1.2	0.80	0.07	0.13
LL	30	625	665	600	675	658	21	9.0	3.0	-20.1	1.4	12.2	1.4	0.81	0.06	0.13
LL	39	635	670	605	680	668	21	9.0	3.0	-20.1	1.4	12.2	1.4	0.80	0.06	0.14
LL	25	635	670	605	690	673	21	8.0	2.8	-20.1	1.5	12.2	1.2	0.80	0.06	0.14
LL	6	645	675	610	765	683	25	4.0	2.0	-20.5	1.2	12.0	1.1	0.83	0.06	0.11

LL	1	660	765	650	770	723	35	4.0	2.0	-20.4	2.1	11.6	0.8	0.83	0.06	0.11
LL	36	670	765	660	770	733	35	4.0	2.0	-20.4	2.1	11.7	0.8	0.83	0.06	0.11
LL	38	675	770	665	770	738	35	4.0	2.0	-20.4	2.1	11.7	0.8	0.83	0.06	0.11
LL	22	685	770	660	865	748	40	4.0	2.0	-20.4	2.1	11.7	0.8	0.83	0.06	0.11
LL	20	765	880	690	895	828	55	1.0	1.0	-20.1		11.7		0.83	0.05	0.12
KM	43	350	510	335	535	438	50									
KM	42	425	535	410	550	498	40									
KM	41	425	550	420	575	513	45									
KM	40	590	645	555	650	623	25									

References

1. L. Calcagnile, G. Quarta, C. Cattaneo, M. D'Elia, Determining 14C Content in Different Human Tissues: Implications for Application of 14C Bomb-Spike Dating in Forensic Medicine. *Radiocarbon* **55**, 1845–1849 (2013).
