# Supplementary material for: Buried in water, burdened by nature—Resilience carried the Iron Age people through Fimbulvinter
Source: PLoS One. 2020 Apr 21;15(4):e0231787. doi: 10.1371/journal.pone.0231787 (PMC7173937; doi:10.1371/journal.pone.0231787)
Supplement: S9 Appendix — (PDF) [file pone.0231787.s009.pdf]

Supporting Information **S9 Appendix. Multidisciplinary evidence** for

Buried in water, burdened by nature – Resilience carried the Iron Age people through Fimbulvinter

Corresponding author: Markku Oinonen

Contributors: Markku Oinonen, Päivi Onkamo, Jukka Palo, Antti Sajantila

S9 Appendix contains: Text, Table O-P

Text

Multidiscliplinary evidence consists of a compilation of repetitions of a theme "Restoration of the Sun and Moon" in the Finnic folklore collection (Table O) and a compilation of the recent genetic studies of Levänluhta individuals (Table P). A cold and dark period with missing Sun and Moon is described frequently within the Finnic folklore and the Table O compiles the repetitions of such a theme.

Table O. Repetitions of a theme "Restoration of the Sun and Moon" in Suomen Kansan Vanhat Runot (Old Poems of Finnish People, www.skvr.fi) collection. In 1849, Elias Lönnrot collected and compiled old runes into the national epic of Kalevala(1) for which the English translation by John Martin Crawford (1888) can be found from http://www.gutenberg.org/ebooks/5185. The first sentences of rune XLIX "The Restoration of the Sun and Moon" describe consequences of a cold and dark period:

But the gold Moon is not shining,
Neither gleams the silver sunlight
In the chambers of Wainola,
On the plains of Kalevala.
On the crops the white-frost settled,
And the cattle died of hunger,
Even birds grew sick and perished.
Men and maidens, faint and famished,
Perished in the cold and darkness,
From the absence of the sunshine,
From the absence of the moonlight.

Ain' on päivä paistamatta,
kuu kulta kumottamatta
noilla Väinölän tuvilla,
Kalevalan kankahilla.
Vilu viljalle tulevi,
karjoille olo kamala,
outo ilman lintusille,
ikävä imehnoisille,
kun ei konsa päivyt paista
eikä kuuhuet kumota.

Part	Number	Location	Collector	Signum	Year	Citation
III1	166.	Soikkola	Länkelä, Jaako	v. III 1 a	1858	Ennen päivättä elettiin, Kuun valotta kuukkaeltiin
III1	167.	Soikkola	Länkelä, Jaako	v. III 1 b.	1858	Elettiin ennen meillä, Ilman kuutta, päivyettä
III1	743.	Narvusi	Porkka, Volmari	II 148.	1881	Enne päivättä elettii, Kuuvalotta kuukkaeltii
III1	747.	Narvusi	Porkka, Volmari	II 152.	1881	Enne päivättä elettii, Kuuvalotta kuukkaeltii
III1	1150.	Soikkola	Porkka, Volmari	III 11.	1881	Elettii enne meillä, … Ilman kuuta, päivyetä
III1	1152.	Soikkola	Porkka, Volmari	III 13.	1881	Elettii ennen meillä, … Ilman kuuta, päivyetä
III1	1155.	Soikkola	Porkka, Volmari	III 16.	1881	Elettii enne meillä, ... Ilman kuuta, päivyetä
III1	1157.	Soikkola	Porkka, Volmari	III 18.	1881	Elettii ennen meillä, … Ilman kuuta, päivyetä
III1	1158.	Soikkola	Porkka, Volmari	III 19.	1881	Elettii ennen meillä, … Ilman k[uuta], p[äivyetä]
III1	1159.	Soikkola	Porkka, Volmari	III 20.	1881	Elettii en[ne] m[eillä], Ilman kuuta, päivyetä
III2	1454.	Soikkola	Porkka, Volmari	III 325.	1881	Elettii enne meillä, ... Ilman kuuta päivyetä
III2	1854.	Narvusi	Alava, Vihtori	VI 466.	1891	Ennen päivättä elettii(n), Kuu-valotta kupaeltii(n)
III2	1936.	Soikkola	Alava, Vihtori	VI 893.	1891	Elettii(n) ennen meill', ... Ilman kuuta, päivyetä.
III2	2212.	Narvusi	Alava, Vihtori	VII 369.	1892	Ennen päivättä elettii, Ilma kuuta, ilma päivää
III3	2632.	Narvusi	Ruotsalainen, J. Fr.	n. 40.	1900	Elettihin enne meillä, Ilman kuuta, päivyetä
III3	2731.	Narvusi	Ruotsalainen, J. Fr.	n. 144.	1900	Elettihi enne meillä, … Ilman kuuta, päivyettä
III3	3767.	Narvusi	Länkelä, Jaako	n. 254.	1858	Paha on päivättä elää, Kuuvalotta kuukkaella
III3	3774.	Narvusi	Länkelä, Jaako	n. 265.	1858	Ennen päivättä elettiin, Kuuvalotta kuukkaeltiin
III3	4075.	Soikkola	Länkelä, Jaako	n. 694.	1858	Elettihin ennen meillä, … Ilman kuuta, päivyetä
III3	4242.	Soikkola	Länkelä, Jaako	n. 990.	1858	Elettihin ennen meillä, … Ilman kuuta, päivyetä
IV2	1700.	Tyrö	Stråhlman, K.	n. 206.	1856	Elättiin ennen meillä, Ilman päivätä elivät, Kupsiit ilman kuutamoita
IV2	1766.	Keski-Inkeri (region)	Stråhlman, K.	n. 286.	1856	Elliit entiset eläjät, Ilman päivättä elliit, Kupsiit ilman kuutamotta
IV2	1840.	Kaprio	Porkka, Volmari	I 35.	1881	Ennen päivöitä eletty, Kupaeltu ilman kuuta
IV3	3168.	Tyrö	Haltsonen, Aatu	n. 119.	1903	Elliit entiset eläjät, Ilman päivöitä elliit, Kupsiit ilman kuutamoo
IV3	3998.	Kaprio	Tynni, Kaapre	n. 16.	1916	Elettii enne meil, Ilmoin päivii elliilt, Kuvasiit ilman kuuta
IV3	4006.	Kaprio	Alava, Vihtori	VI 1167.	1891	Ennen päivöittä eletty, Kupaeltu ilman kuuta

Part	Number	Location	Collector	Signum	Year	Citation
IV3	4057.	Kaprio	Alava, Vihtori	VI 1352 b.	1891	Elettiin ennen meillä, … Kussa teill' kuu pietty,, Kussa päivöi hallikoitu?
V1	797.	Vuole	Saxbäck, Fr. A.	737 a.	1859	Kuin on täällä ennen oltu, Kuin on päivättä eletty, Kupajattu kuutamatta?
XV	684.	Kaprio	Porkka, Volmari	IV. a 1:1.	1883	Enne päivöittä elettiin, kupaeltiin ilman kuuta
XV	1118.	Soikkola	Porkka, Volmari	III. d 88:15.	1883	Elettiin ennen meillä, ilman kuutta, päivyettä
XV	1119.	Soikkola	Porkka, Volmari	III. e 105:2a.	1883	Elettiin ennen meillä, ilman kuutta, päivyettä

Table P. Compilation of the existing aDNA analyses by Lamnidis et al (2018)(2) and Sikora et al (2019)(3) on Levänluhta human remains (teeth). *Based on formal statistical tests on genome-wide data. **JK2065 rejects a cladal position with modern Sámi to the exclusion of most modern Eurasian populations. This individual also rejects a cladal position with Finns.

Sample ID, Lamnidis	Sample ID, Sikora	Mitochondrial haplogroup	Population affiliation*	Verbal comments
JK1968	DA234	U5a1a1/U5aiaia'b'n	Sámi	Sikora: shares the most genetic drift with present-day Sámi. Lamnidis: a clade with modern-day Sámi but not modern-day Finns
JK2065	DA236	K1a4a1b	not Sámi, not Finn**, closest to Scandinavian/Balts	Lamnidis: lacks the Siberian component, closer to modern Lithuanian, Norwegian and Icelandic populations. Sikora: Lower Siberian ancestry than modern Finns --> Scandinavian component
JK1963	DA238	U5b1b1a1	Sámi	Sikora: shares the most genetic drift with present-day Sámi
JK2067	DA237	H1	Sámi	Sikora: shares the most genetic drift with present-day Sámi
JK1970	NA	U5a1a1	Sámi	Lamnidis: slightly lower affinity to central Europe than modern-day Sámi do, still rejects a cladal position with modern-day Finns

References

1. E. Lönnrot, *Kalevala* (1849).
2. T. C. Lamnidis, *et al.*, Ancient Fennoscandian genomes reveal origin and spread of Siberian ancestry in Europe. *Nat. Commun.* **9**, 5018 (2018).
3. M. Sikora, *et al.*, The population history of northeastern Siberia since the Pleistocene. *Nature* **570**, 182–188 (2019).
